# Carbonylation of Polyfluorinated 1-Arylalkan-1-ols and Diols in Superacids

**DOI:** 10.3390/molecules27248757

**Published:** 2022-12-10

**Authors:** Siqi Wang, Yaroslav V. Zonov, Victor M. Karpov, Olga A. Luzina, Tatyana V. Mezhenkova

**Affiliations:** 1 N.N. Vorozhtsov Novosibirsk Institute of Organic Chemistry, Pr. Akademika Lavrent’eva 9, Novosibirsk 630090, Russia; 2 Department of Natural Sciences, Novosibirsk State University, Pirogova Str. 1, Novosibirsk 630090, Russia

**Keywords:** carbonylation of alcohol, superacid, polyfluorinated compound, fluorosulfonic acid, trifluoromethanesulfonic acid, antimony pentafluoride

## Abstract

We describe the carbonylation of a series of mono and dihydroxy derivatives of polyfluorinated alkylbenzenes and benzocycloalkenes with OH groups at benzylic positions using carbon monoxide in the presence of a superacid (TfOH, a TfOH–SbF_5_ mixture, or a FSO_3_H–SbF_5_ mixture). It was shown that the superacid-catalyzed addition of CO to various primary and secondary polyfluorinated alcohols and diols gives the corresponding mono- and dicarboxylic acids or lactones. The efficiency of various superacids depending on alcohol structure was evaluated, and FSO_3_H–SbF_5_ yielded the best results in most transformations. The addition of CO to secondary 1-arylalkan-1-ols containing vicinal fluorine atoms was found to be accompanied by elimination of HF with the formation of *α,β*-unsaturated aryl-carboxylic acids. In contrast to primary and secondary alcohols, conversion of tertiary perfluoro-1,1-diarylalkan-1-ols into carbonylation products is not complete, and the resulting carboxylic acids are easily decarboxylated after water treatment of the reaction mixture.

## 1. Introduction

Organofluorine compounds are important for basic research in organic chemistry and for its applications, particularly to materials science, biomedicine, and agriculture [1,2,3,4,5,6,7,8,9,10]. Therefore, the development of new approaches to their synthesis is of obvious interest. Currently in hydrocarbon chemistry, there are many known acid-catalyzed carbonylation reactions of alcohols, alkyl halides, and other compounds proceeding via CO addition to carbocations generated from these compounds in acidic systems and leading to the formation of carboxylic-acid derivatives [11,12,13]. In spite of the variety of reactions of fluorinated cations, acid-catalyzed carbonylation of polyfluorinated compounds had not been known before our recent reports [14,15,16,17], and a reverse reaction—decarbonylation of fluorinated acyl halides under the action of Lewis acids—is commonly observed [18,19,20]. Known examples of acid-catalyzed carbonylation of organofluorine compounds (structures where a fluorine atom or polyfluoroalkyl group is separated from the reaction center by no more than 4–5 bonds and can influence it) are limited to reactions of alcohols Ar_2_RCOH (R = H, CH_3_) containing no more than two fluorine atoms [21] as well as mono-(polyfluoroalkyl)-substituted adamantyl halides [22,23] with a mixture of formic and sulfuric acids resulting in carboxylic acids.

Transition metal complex–catalyzed carbonylation at a C(*sp*^3^)–X bond (X = Hal, OR) or hydrocarboxylation and hydroesterification of alkenes, as an alternative to acid-catalyzed carbonylation, has more examples for some fluorinated compounds, but the range of substrates is quite small. For instance, there is evidence of Pd- or Co-catalyzed carbonylation of primary benzyl derivatives ArCH_2_X (X = Cl, Br, I, OH, OCOOR) [24,25,26,27,28,29,30,31,32,33] or esters of allylic alcohols [34,35] with only one fluorine atom or CF_3_ group in the aryl group or at the double bond. Pd-, Ru-, or Co-catalyzed carbonylation of polyfluorinated alkyl iodides [36,37,38,39,40,41,42] or Pd-catalyzed hydrocarboxylation and hydroesterification of perfluoroalkyl-substituted ethylenes using CO [43,44,45,46,47] can produce aliphatic carboxylic acids and their derivatives with a saturated perfluorinated moiety attached to a *β*-carbon atom or to carbon atoms more distant from the COOH group. For the synthesis of esters of *α*-CF_2_H-substituted arylacetic acids, Pd-catalyzed hydroesterification of 1,1-difluoro-2-arylethylenes has been proposed [48].

In our previous research, we have found that perfluorinated alkyl- and phenyl-benzocyclobutenes [14,15] and their carbonyl derivatives [17] undergo carbonylation/four-membered–ring opening tandem reactions under the action of carbon monoxide at atmospheric pressure in the presence of SbF_5_. In these reactions, irreversible four-membered–ring transformations promote the conversion of polyfluorobenzocyclobutenes into the desired products. In the CO–SbF_5_ system, the possibility of carbonylation of per- and polyfluorinated indanes and tetralins giving rise to fluorocarbonyl derivatives and corresponding acids after hydrolysis has been demonstrated too [16], and the transformation proceeds as an equilibrium process without skeletal rearrangements, while the degree of conversion substantially depends on the substrate structure. The discovered transformations are first examples of the functionalization of perfluorinated compounds via a carbonylation reaction.

In this research field, we have studied the possibility of H^+^-catalyzed carbonylation of polyfluorinated alcohols. The current work describes the carbonylation of a series of mono and dihydroxy derivatives of polyfluorinated alkylbenzenes and benzocycloalkenes with OH groups at benzylic positions by means of carbon monoxide in the presence of such a superacid as TfOH, a TfOH–SbF_5_ mixture, or an FSO_3_H–SbF_5_ mixture as a way to transform the above compounds into carboxy derivatives. Polyfluorinated α-aryl-substituted aliphatic carboxylic acids are of interest because this class of compounds with fluorine atoms in both aliphatic and aromatic moieties has been investigated as bioactive compounds or used for synthesis thereof [49,50,51,52,53,54], as is the case for halogenated derivatives of benzocycloalkene-1-carboxylic acids [55,56,57,58,59]. The presence of a polyfluorinated aromatic ring in the molecule gives an opportunity for further synthetic modification via S_n_Ar substitution. Additionally, the perfluorinated aryl group has unique physicochemical properties that can be used in the design of materials. For instance, derivatives of pentafluorophenyl acetic acid have been utilized to create semiconductor and photoluminescent materials [60,61], gas sensors [62], and supramolecular associates for biological applications [63,64].

## 2. Results and Discussion

### 2.1. Carbonylation of Primary and Secondary Alcohols C_6_F_5_CH(OH)R (R = H, C_6_F_5_, CF_3_), and Selection of a Superacid

It was shown that 1-phenylalkan-1-ols **1a**–**c** (Table 1) can be converted to corresponding acids **2** by carbonylation in superacids using carbon monoxide under atmospheric pressure, followed by water treatment of the reaction mixture. In TfOH, pentafluorobenzyl alcohol **1a** adds CO, and heating to 50 °C is required for a fast and complete reaction, whereas diphenylmethanol **1b** easily reacts at room temperature (r.t.). In contrast, alcohol **1c** failed to be carbonylated in TfOH, and its acidity is apparently insufficient to generate the respective CF_3_-substituted intermediate carbocation. The use of a mixture of TfOH and SbF_5_ (1:1), which has higher acidity, made it possible to obtain the carbonylation product, acid **2c**, at r.t. Two equiv. of this mixture allowed to achieve 95% conversion of the starting alcohol into acid **2c** in 2 h. As the reaction time was extended, the conversion approached 100%. Reducing the amount of the acid mixture to 1.5 equiv. considerably reduced the content of acid **2c**. An FSO_3_H–SbF_5_ mixture (1:1) compared to TfOH–SbF_5_ (1:1) in the amount of 2 equiv. worked more efficiently, yielding complete conversion of alcohol **1c** to acid **2c** in 2 h, probably due to its higher acidity [65].

### 2.2. Carbonylation of Tertiary Perfluorinated 1,1-Diarylalkan-1-ols

**Scheme 1:**
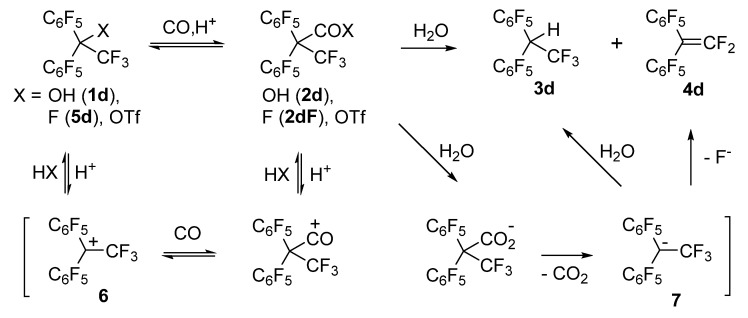
Transformations of compound **1d** in the reaction with CO in TfOH–SbF_5_ followed by water treatment.

Alcohols **1d**–**f** (Table 2) did not react with CO in TfOH at r.t. despite the presence of two aryl moieties that can stabilize the intermediate cation. With an increase in the reaction temperature, the substitution of fluorine atoms in the aryl moiety began to occur instead of carbonylation. It was noted that TfOH–SbF_5_ (1:1) enables us to carry out the carbonylation of alcohol **1d**; however, after hydrolysis of the reaction mixture, the resulting acid **2d** was easily decarboxylated, giving hydro derivative **3d** and alkene **4d** (see Scheme 1). Replacing the hydrolysis with treatment of the reaction mixture with methanol led to the formation of methyl ester **2dMe**; however, decarboxylation products **3d** and **4d** also formed. Another difference from the carbonylation of alcohols **1a**–**c** was the incomplete conversion of tertiary alcohol **1d** into carbonylation products: the reaction mixtures also contained perfluorodiphenylethane **5d** and the starting alcohol. The highest conversion of compound **1d** to carbonylation products in the TfOH–SbF_5_ medium was achieved with the help of 2 equiv. of the superacid (total content of products **3d** and **4d**: ~30%). Extension of the reaction time from 5 to 24 h barely changed the reaction outcome, whereas an increase in the amount of the superacid to 4 equiv. or its decrease to 1.2 equiv. substantially diminished the concentration of products **3d** and **4d** in the reaction mixture. Using 2 equiv. of the mixture of FSO_3_H and SbF_5_ (1:1) instead of TfOH–SbF_5_ afforded a slightly higher conversion of alcohol **1d** to carbonylation products (~45%); in this case, the introduction of a solvent (C_6_F_6_) was required to reduce the viscosity of the reaction mixture for efficient mixing.

The results on carbonylation of compound **1d** can be explained (Scheme 1) by the presence (in the TfOH–SbF_5_ medium) of an equilibrium between noncarbonylated forms (alcohol **1d** or/and its triflate: fluoro derivative **5d**) and carbonylation products (acid **2d**, the corresponding acyl fluoride **2dF**, or/and acyl triflate). The feasibility of carbonylation of perfluorodiphenylethane **5d** in this medium was confirmed by a separate experiment. The equilibrium state depended on the amount of the superacid: its excess shifted the equilibrium toward decarbonylation, whereas its deficit hindered the generation of cation **6**, which interacts with CO. After water treatment of the reaction mixture, all carbonylation products were hydrolyzed into acid **2d**. In an aqueous medium, its deprotonation was possible, and the resulting carboxylate anion—owing to steric hindrance and the electron-withdrawing effect of substituents—easily eliminated CO_2_ with the formation of carbanion **7**. The latter either added a proton or eliminated a fluoride ion, thereby yielding **3d** and **4d**, respectively. The formation of alkene **4d** as a result of the transformation of hydro derivative **3d** during treatment of the reaction mixture is unlikely; it was found that such elimination of HF, even in the presence of a base, is possible only under forcing conditions (NEt_3_, CaH_2_, 130 °C). The emergence of acid **2d** and acyl fluoride **2dF**, along with compounds **3d** and **4d**, could be detected after water treatment during the extraction of products with a mixture of CH_2_Cl_2_ and Et_2_O; the concentration of acid **2d** in the extract gradually declined with time, while the concentration of products **3d** and **4d** went up. Extraction with pure Et_2_O gave only compounds **3d** and **4d**.

The reaction of phenylindanol **1e** with CO in FSO_3_H–SbF_5_ (Table 2) followed by hydrolysis of the reaction mixture also generated products **3e** and **4e**; the conversion of **1e** into carbonylation products was slightly higher than that for compound **1d** (the total content of products **3e** and **4e**: ~60%) but also incomplete. In a similar reaction of phenyltetralinol **1f**, the total level of products **3f** and **4f** was much lower (~20%), which can be explained by large steric hindrances for CO addition. The conversion to carbonylation products **3e** and **4e** for alcohol **1e** was close to that obtained in the carbonylation of perfluorophenylindane **5e** in SbF_5_ (55% conversion). On the other hand, for alcohol **1f**, the conversion to carbonylation products was substantially higher than that in the reaction of perfluorophenyltetralin **5f** with CO-SbF_5_ (5%) [16].

**Scheme 2:**
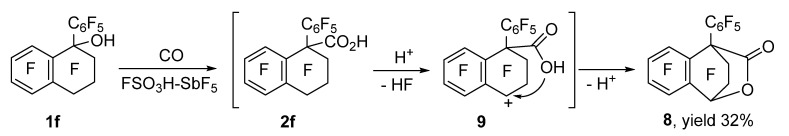
Lactone **8** formation during the carbonylation of compound **1f** in FSO_3_H–SbF_5_.

Aside from compounds **3f** and **4f**, the reaction of alcohol **1f** generated lactone **8** as another carbonylation product (Scheme 2). The content of lactone **8** went up with increasing reaction time, temperature, and amount of the superacid. On the contrary, the concentration of products **3f** and **4f** strongly decreased (Table 2). Lactone **8** emerged via the cyclization of acid **2f**; apparently, cation **9** generated from it in the FSO_3_H–SbF5 medium undergoes an intramolecular attack by the oxygen atom of the carboxyl group. In the carbonylation of phenylindanol **1e**, lactone formation was not observed.

### 2.3. Carbonylation of Secondary Polyfluorinated 1-Arylalkan-1-ols and Concomitant Elimination of HF

It was demonstrated that carbonylation of secondary polyfluorinated 1-arylalkan-1-ols with fluorine atoms at the *β*-position toward the hydroxyl group (in superacid FSO_3_H–SbF_5_ or TfOH–SbF_5_) can be accompanied by partial or complete elimination of HF, thus giving rise to *α,β*-unsaturated carboxylic acids **10** (Table 3). For instance, the reaction of phenylpropanol **1g** with CO in TfOH–SbF_5_ at r.t., followed by hydrolysis of the reaction mixture, produced acid **2g** with a small admixture of unsaturated acid **10g**. A higher reaction temperature promoted more complete elimination of HF; acid **10g** became the main product at 70 °C. In the FSO_3_H–SbF_5_ medium, the proportion of elimination product **10g** was higher than that in TfOH–SbF_5_. An important factor is also the nature of the substituents at the nascent double bond; this nature affects the energy of its formation and accordingly the ease of elimination. Thus, in contrast to phenylpropanol **1g**, carbonylation of phenylethanol **1c** after heating under similar conditions did not give appreciable amounts of the corresponding unsaturated acid **10c** with two fluorine atoms at the double bond.

Some structural features can improve the efficiency of conjugation of the resulting double bond with the aryl moiety, for example, its fixation in the plane of the aryl moiety probably contributed to the elimination; as a result, carbonylation of tetralinol **1h** gave considerable amounts of acid **10h** already at r.t. A greater amount of the superacid caused more complete elimination (Table 3, entries 5 and 7), whereas extension of the time of the process had little effect (Table 3, entries 5 and 6).

A decrease in the size of the aliphatic ring from the six-membered to five-membered hindered the elimination; for example, carbonylation of indanol **1i** in the FSO_3_H–SbF_5_ medium did not give HF elimination products at r.t., in contrast to its homolog **1h**. On the other hand, under these conditions, a partial transformation of the CF_2_ group into a carbonyl group was observed; as a consequence, the reaction mixture also contained—alongside acid **2i**—keto acid **2j** and apparently other 3-R-2,2,4,5,6,7-hexafluorindan-1-ones (R = F, OSO_2_F), which are not carbonylation products. Previously, carbonylation of 1,1,2,2,3,4,5,6,7-nonafluoroindane was conducted in a CO–SbF_5_ system, and acid **2i** was the only product obtained [16]. The transformation of the CF_2_ group of indanol **1i** or of products of its transformation into a carbonyl group is mediated by elimination of the fluoride ion in the superacid and a reaction of the emerging carbocation with O-nucleophiles contained in the reaction medium (for example, RCOOH [66]), thereby leading to the carbonyl derivatives. Lower stability of polyfluorinated tetralin-1-yl cations compared to respective indan-1-yl cations [67] explains the absence of transformations of the benzyl CF_2_ group during the carbonylation of tetralinol **1h** at r.t. When the temperature of the reaction of indanol **1i** with CO in the FSO_3_H–SbF_5_ medium was raised to 70 °C, both the carbonylation and transformation of the CF_2_ group into the carbonyl group proceeded completely; futher, the elimination of HF also occurred, which produced a mixture of acids **2j** and **10j**. A mixture of acids **2j** and **10j** also formed in the reaction of hydroxyketone **1j** with CO in the FSO_3_H–SbF_5_ medium at 70 °C. 

In contrast to compound **1i**, indanols **1k** and **1l** (in which one or both benzyl fluorine atoms are replaced by pentafluoroethyl groups) in the reaction with CO in FSO_3_H–SbF_5_ at r.t. smoothly converted into corresponding acids **2k** and **2l**. Elimination of HF with the formation of unsaturated acid **10l** from alcohol **1l**, as in the case of indanol **1i**, happened only at a higher reaction temperature.

The presence of hydrocarbon groups in the aliphatic part of the molecule in some cases required lower acidity of the medium for successful carbonylation. For instance, for indanol **1m**, carbonylation generating acid **2m** in the FSO_3_H–SbF_5_ medium was accompanied by considerable resinification, which could be avoided in TfOH–SbF_5_ (5:1) at 50 °C; in pure TfOH, almost no carbonylation of indanol **1m** occurred.

The formation of an aromatic system through the elimination of HF accompanying the carbonylation could ensure rapid and complete elimination even at r.t. For example, in the reaction of heterocyclic alcohol **1n** with CO in the FSO_3_H–SbF_5_ medium (in the TfOH–SbF_5_ medium, the outcome was similar), after hydrolysis of the reaction mixture, benzofurancarboxylic acid **10n** arose, and acid **2n** was not detectable. The formation of a cationic species with a benzofuran moiety (acid **10n** protonated at carbonyl oxygen or its acyl cation) was registered before the water treatment of the reaction mixture, thus confirming that the elimination of HF took place in the FSO_3_H–SbF_5_ medium.

It should be noted that in most of the carbonylation reactions mentioned above and involving the concomitant elimination of HF, there was no full conversion to elimination product **10**; furthermore, in contrast to some factors such as reaction temperature or the amount of the superacid, the increase in the reaction time had little effect on the ratio of acids **2** and **10**. It can be theorized that the elimination of HF is a reversible process, but when we introduced product **10j** into the reaction mixture obtained after the carbonylation of alcohol **1n** and kept the resulting mixture either at r.t. or at 70 °C, we failed to detect (after water treatment) acid **2j** as an HF addition product.

**Scheme 3:**
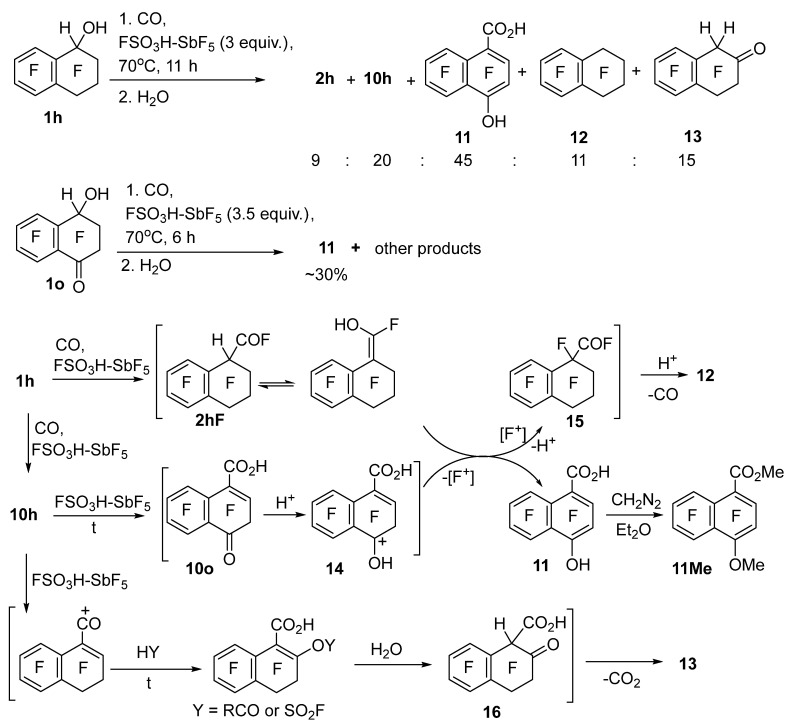
Carbonylation of compounds **1h** and **1o** in FSO_3_H–SbF_5_ at 70 °C.

The interaction of tetralinol **1h** with CO in FSO_3_H–SbF_5_ at 70 °C (Scheme 3), in contrast to the analogous reaction of indanol **1i** (Table 3, entry 9), did not generate the corresponding ketoacids. The main product was naphthalenecarboxylic acid **11**; in addition, the reaction mixture contained acids **2h** and **10h**, perfluorotheralin (**12**), and tetralon **13**. Acid **11** was isolated as dimethylated derivative **11Me** after treatment of the reaction mixture with diazomethane. Carbonylation of hydroxyketone **1o** under similar conditions resulted in a complex mixture of compounds, among which acid **11** was the main one as well (Scheme 3).

To explain the simultaneous formation of naphthalenecarboxylic acid **11** and tetralin **12** in the reaction of alcohol **1h** with CO in FSO_3_H–SbF_5_ at 70 °C, the following sequence of transformations can be proposed (Scheme 3). Acid **10h** under the reaction conditions is partially converted to carbonyl derivative **10o** through the generation of the corresponding cation and its reaction with the O-nucleophiles contained in the reaction medium. Protonation of compound **10o** gives naphthalinonium cation **14**, the reaction of which with the enol form of acyl fluoride **2hF** (or mixed anhydride RCO_2_SO_2_F)—as a consequence of a redox process with the transfer of a fluorine atom—gives acid **11** and perfluorinated acyl fluoride **15**. The latter is decarbonylated under these reaction conditions, thereby yielding tetralin **12**. The possibility of decarbonylation of compound **15** is consistent with the finding that tetralin **12** did not give a carbonylation product in the CO–SbF_5_ system because the equilibrium of this reaction is shifted toward the starting tetralin [16]. The lower concentration of tetralin **12** in the reaction mixture compared to acid **11** can be attributed to its low solubility in the inorganic superacid in combination with its appreciable volatility; accordingly, it can be partially carried away by the CO flow. Tetralone **13** seems to come from another kind of transformation of acid **10h** (Scheme 3, bottom pathway). It is possible that the reaction of the acyl cation deriving from acid **10h** with O-nucleophiles present in the reaction medium (RCO_2_H and FSO_3_H) drives the replacement of the fluorine atom by oxygen at the conjugated double bond, whereas subsequent hydrolysis during water treatment and decarboxylation of resultant ketocarboxylic acid **16** produce ketone **13**.

### 2.4. Carbonylation of Diols

**Scheme 4:**
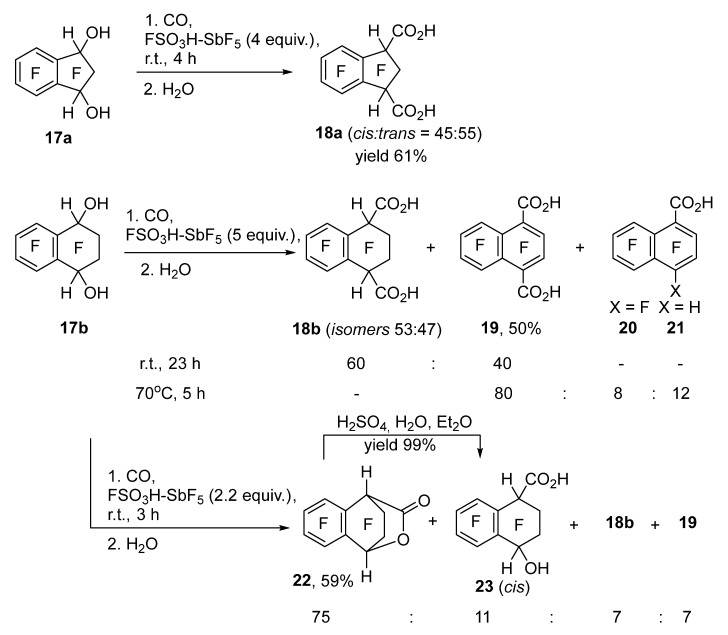
Carbonylation of diols **17a** and **17b** in FSO_3_H–SbF_5_.

Carbonylation of benzylic dihydroxy derivatives of polyfluorinated arylalkanes and benzocycloalkenes, depending on the structure, led to the addition of one or two CO molecules. The reaction of indanediol **17a** with CO in FSO_3_H–SbF_5_ selectively gave diacid **18a** as a mixture of *cis-* and *trans-*isomers (Scheme 4); no HF elimination products formed at r.t., as in the case of other indanols tested above. On the other hand, in the reaction of tetralindiol **17b** with CO in the presence of 5 equiv. of FSO_3_H–SbF_5_, the addition of two CO molecules was accompanied by the elimination of two HF molecules already at r.t., resulting in a mixture of acids **18b** and **19**. Products of the elimination of one HF molecule were absent; apparently, the elimination of the second HF molecule proceeded faster than that of the first one because this led to the aromatic naphthalene system. An increase in the reaction temperature to 70 °C caused complete elimination of HF with the formation of diacid **19**; the reaction mixture also contained admixtures of naphthalenecarboxylic acids **20** and **21** arising in side reactions; they were isolated and characterized after esterification as a mixture of ethyl esters **20Et** and **21Et**. Acid **20** apparently is a consequence of OH group replacement by fluorine in the monocarbonylation product (or in the starting diol) and the elimination of two HF molecules. The pathway to hydro derivative **21** is not clear; it was demonstrated that diacid **19** does not transform into it under the reaction conditions in question.

After a reduction in the amount of FSO_3_H–SbF_5_ to 2.2 equiv. in the reaction of diol **17b** at r.t., it was possible to achieve selective monocarbonylation, which was accompanied by the assembly of a lactone ring involving the second OH group leading to lactone **22**. Hydroxy acid **23** was also present in the reaction mixture, along with dicarbonylation products **18b** and **19**. In contrast to diacid **18b**, hydroxy acid **23** was present in the mixture as a single diastereomer, which apparently has *cis*-configuration and arises via partial hydrolysis of lactone **22** during water treatment of the reaction mixture. Hydrolysis of lactone **22** by dilute sulfuric acid caused selective formation of the same diastereomer of acid **23**. For indandiol **17a**, selective preparation of the monocarbonylation product was not achieved.

Monocarbonylation affording lactones also occurred when diols **17c**–**e** were reacted with CO in the presence of a superacid (Table 4). Carbonylation of diol **17c** in FSO_3_H–SbF_5_ (1:1), as in the case of indanol **1m**, was accompanied by considerable resinification. The use of less acidic system TfOH–SbF_5_ (6:1) helped the carbonylation to generate lactone **24c**. Of note, carbonylation of diols **17c**–**e** required more forcing conditions as compared to respective alcohols **1a**–**c**. For instance, diol **17c**, carbonylation in pure TfOH proceeded with difficulty, even when heated. Pentafluorophenyl-substituted diol **17d** did not react with CO either in TfOH at 50 °C or even in TfOH–SbF_5_ (7:2) at r.t., giving only the corresponding phthalane as a diol cyclization product. By contrast, in the FSO_3_H–SbF_5_ medium at r.t., diol **17d** easily added CO, thereby yielding a mixture of isomeric lactones **24d** and **25d** in a ratio close to 1:1. Carbonylation of diol **17e** in FSO_3_H–SbF_5_ took place upon heating to 50 °C, and the destabilizing effect of the CF_3_ group on the cationic center induced regioselective carbonylation in the CH_2_OH moiety, giving rise to lactone **24e**. For *ortho*-substituted diols **17c**–**e**, products of addition of two CO molecules were not observed, while corresponding diacids **18f** and **18g** formed from *meta*- and *para*-substituted diols **17f** and **17g.** Meta-isomer **17f** readily reacted with CO in TfOH–SbF_5_ (7:1), whereas para-isomer **17g** required the use of FSO_3_H–SbF_5_.

### 2.5. Esterification and Elimination Reactions of the Carbonylation Products

**Scheme 5:**
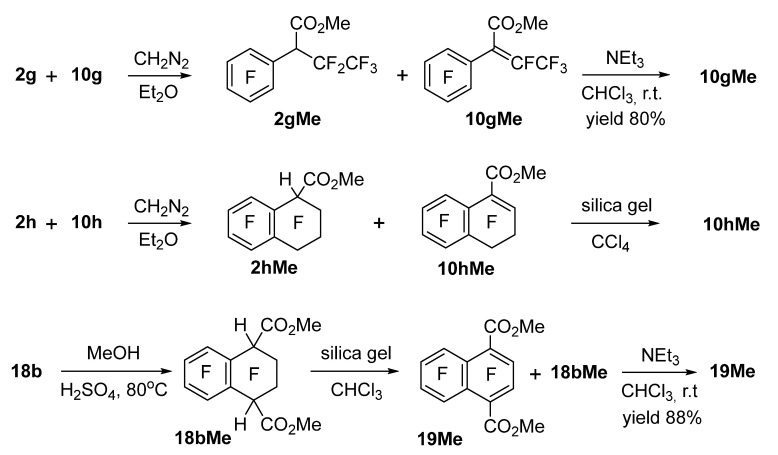
Esterification of acids **2g**, **2h**, **10g**, **10h**, and **18b** in reaction mixtures generated by the carbonylation procedures and HF elimination from esters **2gMe**, **2hMe**, and **18bMe**.

In some cases, esterification was performed to isolate and purify carboxylic acids obtained in carbonylation reactions. Furthermore, the feasibility of conversion of several acids and esters into *α,β*-unsaturated derivatives by elimination of HF was tested. Thus, mixtures of esters **2gMe** + **10gMe** and **2hMe** + **10hMe** were obtained by the reaction of the respective acid mixtures with diazomethane (Scheme 5). Ester **2gMe** could be smoothly converted into product **10gMe** under the influence of NEt_3_ in chloroform; elimination in ester **2hMe** proceeded fully, even during column chromatography on silica gel. Esterification of a mixture of diacids **18b** and **19** with methanol in the presence of H_2_SO_4_ made it possible to selectively obtain ester **18bMe** because acid **19** became esterified much more slowly. Nonetheless, the purification of ester **18bMe** by silica gel chromatography afforded a mixture of esters **18bMe** and **19Me**, owing to partial elimination of HF; treatment of the obtained mixture with NEt_3_ in chloroform led to ester **19Me** as the only product.

**Scheme 6:**
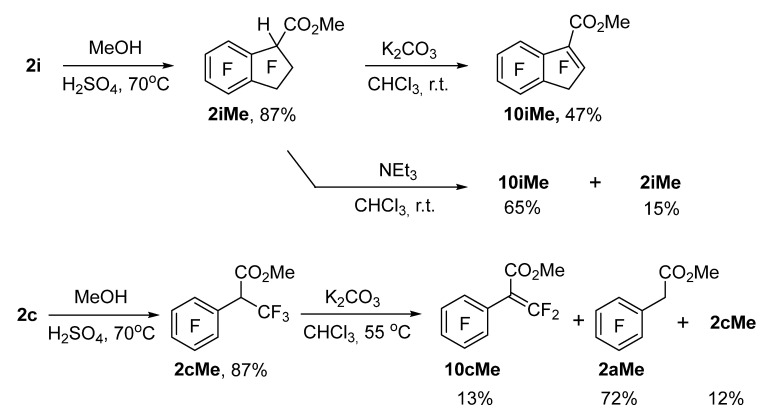
Synthesis of esters **2cMe** and **2iMe** and HF elimination under the action of NEt_3_ and K_2_CO_3_.

Esterification of acids **2c** and **2i** with methanol in the presence of H_2_SO_4_ gave corresponding esters **2cMe** and **2iMe** (Scheme 6). The reaction of ester **2iMe** with NEt_3_ in chloroform generated compound **10iMe** as the main product, but complete conversion of the starting compound with the increasing reaction time was not achieved. This finding is probably due to the reversibility of this process. When K_2_CO_3_ was used as a base, the eliminated HF produced insoluble salts, which helped to achieve full conversion of ester **2iMe**. 

An attempt to carry out the conversion of ester **2cMe** into product **10cMe** in a reaction with NEt_3_ in chloroform was utterly unsuccessful: ester **2cMe** mostly did not change, giving only a small amount of ester **2aMe** without a CF_3_ group (probably owing to a reaction with traces of water). These data are also consistent with the presumed reversibility of the HF elimination process; the equilibrium in the case of ester **2cMe** almost completely shifted toward the starting compound because the formation of compound **10cMe** with two fluorine atoms at the double bond is thermodynamically less favorable. Nonetheless, it can act as an intermediate in nucleophilic reactions involving CF_3_ group transformation. The reaction of **2cMe** with K_2_CO_3_ in chloroform afforded ester **10cMe**, but at r.t., it was too slow. When the temperature was raised to 55 °C, ester **2aMe** became the main product, which is apparently a consequence of further transformations of compound **10cMe** in the reaction with O-nucleophiles.

**Scheme 7:**
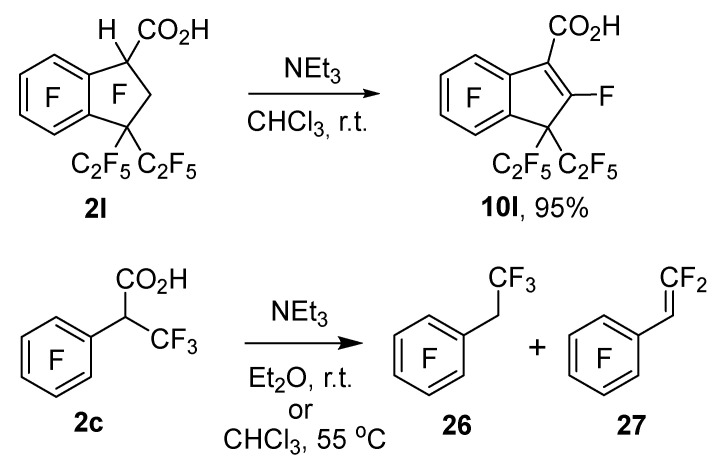
Transformation of acids **2c** and **2l** under the influence of NEt_3_.

For acid **2l**, it was feasible to implement selective elimination of HF with the formation of acid **10l** in the reaction with NEt_3_ without esterification of the carboxyl group beforehand (Scheme 7). This process is apparently facilitated by the steric effect of the two bulky perfluoroethyl groups, which, on one hand, promotes elimination (because this effect reduces their repulsion from other substituents in the five-membered cycle) and, on the other hand, prevents subsequent nucleophilic addition to the nascent double bond. Attempts to carry out similar selective elimination under the action of NEt_3_ with a number of other carboxylic acids obtained in this work were futile. The reactions are complicated by concomitant decarboxylation with or without HF elimination. For example, the interaction of acid **2c** with NEt_3_ in Et_2_O at r.t. or in chloroform at 55 °C predominantly gave ethylbenzene **26** and styrene **27**.

### 2.6. Starting Compounds

**Scheme 8:**
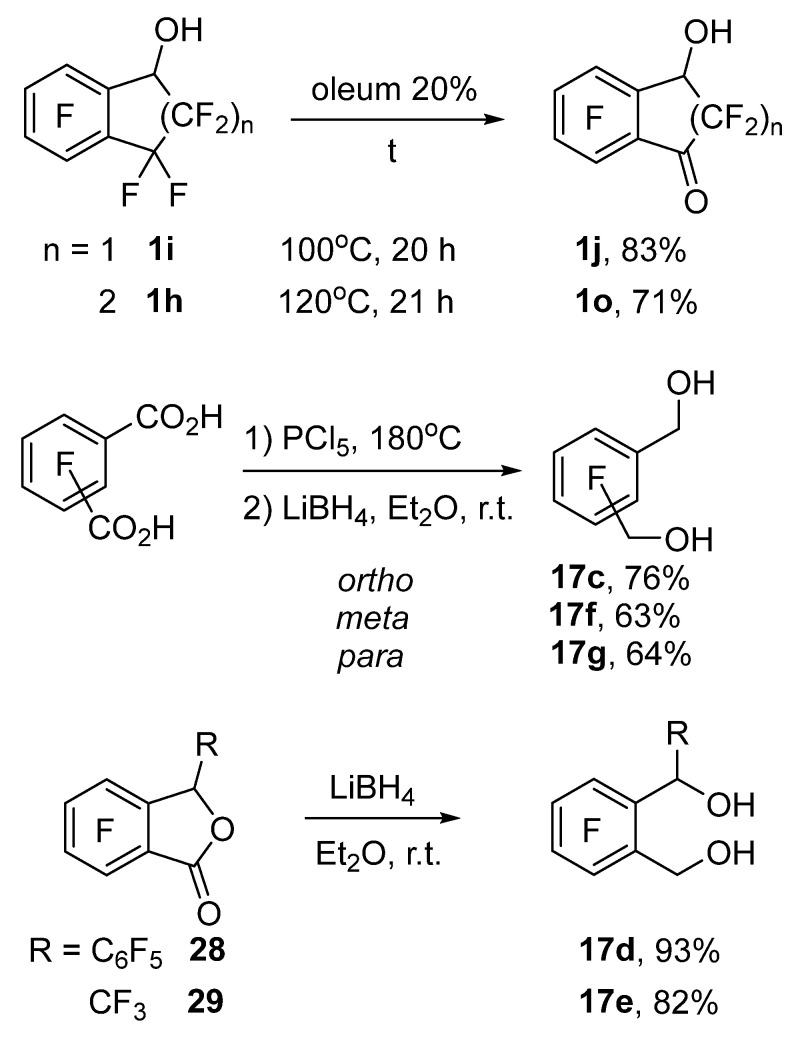
Synthesis of starting alcohols **1j**,**o** and diols **17c**–**g**.

Carbonyl derivatives **1j** and **1o** were synthesized by the heating of alcohols **1i** and **1h** with 20% oleum (Scheme 8). Diols **17c**,**f**,**g** were derived from the corresponding isomers of dicarboxylic acids, which were first converted into diacyl chlorides via a reaction with PCl_5_ and then reduced with LiBH_4_. Diols **17d** and **17e** were obtained by reduction of phthalides **27** and **28** with LiBH_4_. The other starting compounds were prepared according to literature data: alcohols **1a,b** [68], **1c**,**g**,**h**,**i**,**k**,**l**,**m**,**n** and **17a**,**b** [69], **1d** [70], **1e** [71], **1f** [72], and phthalides **28** [73] and **29** [74].

### 2.7. Structural Analysis of Compounds

The structures of the compounds were established by means of high-resolution mass spectrometry (HRMS), elemental analysis, and spectral characteristics. Signals in NMR spectra of compounds were assigned on the basis of chemical shifts of the signals, their fine structure, and integral intensities. Compounds **2a [75]**, **2i**, **3e**, **4e** [16], **4f** [76], **5d** [70], **5e** [71], **5f** [72], **12** [77], **17d** [69], **20** [78], **26** [79], and **27** [80] were identified by comparison of ^19^F NMR findings with the literature data (NMR spectra in Supplementary Materials). Structures of *E*- and *Z*-isomers of compounds **10g** and **10gOMe** were determined by means of through-space *J*_F,F_ values between closely located fluorine atoms. For instance, for the *Z*-isomers, *J*_CF3,F(ortho)_ = 2 Hz, whereas for the *E*-isomers, it is not observed; on the other hand, *J*_3,ortho_ < 2 Hz for the *Z*-isomers and 11–12 Hz for the *E*-isomers. The position of the substituent (C_6_F_5_ or CF_3_) in lactones **24d**, **25d**, and **24e** was identified with the help of ^1^H NMR spectra. Chemical shifts of CH_2_ protons in the Ar_F_CH_2_CO moiety of compounds **24d** and **24e** were found to not exceed 4 ppm, whereas in the Ar_F_CH_2_O moiety of compound **25d**, they are more than 5 ppm. 

## 3. Materials and Methods

Analytical measurements and spectral analyses were carried out at the Multi-Access Chemical Research Center SB RAS. IR spectra were acquired on a Bruker Vector 22 IR spectrophotometer. ^19^F, ^1^H, and ^13^C NMR spectra were recorded on a Bruker AV 300 instrument (282.4 MHz, 300 MHz, and 75.5 MHz, respectively). Chemical shifts are given in *δ* ppm from CCl_3_F (^19^F) and TMS (^1^H, ^13^C). C_6_F_6_ (^19^F, −162.9 ppm), CHCl_3_ (^1^H, 7.24 ppm), acetone-*d*_5_ (^1^H, 2.04 ppm), DMSO-*d*_5_ (^1^H, 2.50 ppm), and CDCl_3_ (^13^C, 76.9 ppm) served as internal standards. Gas chromatography coupled with mass spectrometry (GC-MS) was performed on Hewlett Packard G1081A combined with Hewlett Packard 5890 with mass selective detector HP 5971 (EI 70 eV). Molecular masses of the compounds were determined by HRMS with a Thermo Electron Corporation DFS instrument (EI 70 eV). The progress of reactions and levels of products in reaction mixtures were monitored with the help of ^19^F NMR spectroscopic data. 

FSO_3_H and TfOH were purchased from commercial sources (99% purity); antimony pentafluoride was distilled under atmospheric pressure (bp 142–143 °C); carbon monoxide was prepared by decomposition of formic acid in concentrated sulfuric acid and was additionally dried by passing it through a layer of concentrated sulfuric acid. Et_2_O and CHCl_3_ for HF elimination reactions were dehydrated over 3A molecular sieves.

### 3.1. A Typical Carbonylation Procedure

A mixture of an alcohol and superacid was intensively stirred in a round-bottom glass flask (10 mL) in a slow flow of CO under atmospheric pressure. The mixture was poured into 10–15 mL of 5% hydrochloric acid and extracted three times with 4 mL of a CH_2_Cl_2_–Et_2_O mixture (3:1) (if not specified otherwise). The extract was dried over MgSO_4_ and analyzed by ^19^F NMR spectroscopy. Reagent amounts, reaction conditions, and procedures for separation of mixtures and product isolation are detailed in the experiments below.

### 3.2. Carbonylation of (Pentafluorophenyl)methanol *(**1a**)*

Alcohol **1a** (0.600 g, 3.03 mmol) and TfOH (2.502 g, 16.68 mmol) (molar ratio 1.0:5.5), according to the typical procedure (Section 3.1) (6 h, 50 °C, extraction with CH_2_Cl_2_), after evaporation of the solvent and sublimation (120 °C, 3 Torr), gave 0.613 g of acid **2a** (yield 90%). 

### 3.3. Carbonylation of Bis(pentafluorophenyl)methanol *(**1b**)*

Alcohol **1b** (0.620 g, 1.70 mmol) and TfOH (1.790 g, 11.93 mmol) (molar ratio 1:7), according to the typical procedure (Section 3.1) (3 h, r.t., extraction with CH_2_Cl_2_), after evaporation of the solvent, afforded 0.650 g of acid **2b** (yield 97%). 

*2,2-bis(pentafluorophenyl)acetic acid* (**2b**). White solid, mp 109.5–111.8 °C. IR (KBr) ν, cm^−1^: 1732 (C=O), 1527, 1508 [fluorinated aromatic ring (FAR)]. ^1^H NMR (CDCl_3_): *δ* 11.87 (s, 1H, COOH), 5.60 (s, 1H, CH). ^19^F NMR (CDCl_3_): *δ* −140.8 (m, 4F, F-*ortho*), −153.0 (tt, 2F, *J_para,meta_* = 21.0 Hz, *J_para,ortho_* = 2.5 Hz, F-*para*), −161.7 (m, 4F, F-*meta*). HRMS, *m/z*: 391.9892 (M^+^). Calcd. for C_14_H_2_F_10_O_2_: M = 391.9890.

### 3.4. Carbonylation of 2,2,2-Trifluoro-1-(pentafluorophenyl)ethan-1-ol *(**1c**)*

*a.* Alcohol **1c** (0.493 g, 1.84 mmol) and TfOH (1.350 g, 9.00 mmol) (molar ratio 1:5), according to the typical procedure (Section 3.1) (11 h, 50 °C, extraction with CH_2_Cl_2_), after evaporation of the solvent, gave 0.484 g of starting compound **1c**.

*b.* Alcohol **1c** (0.500 g, 1.88 mmol), TfOH (0.580 g, 3.87 mmol), and SbF_5_ (0.827 g, 3.83 mmol) (molar ratio 1:2:2), according to the typical procedure (Section 3.1) (2 h, r.t.), produced a mixture of compounds containing 95% of acid **2c**.

*c.* Alcohol **1c** (0.356 g, 1.34 mmol), TfOH (0.400 g, 2.67 mmol), and SbF_5_ (0.581 g, 2.67 mmol) (molar ratio 1:2:2), according to the typical procedure (Section 3.1) (20 h, r.t.), after evaporation of the solvent and sublimation (120 °C, 1 Torr), gave 0.358 g of acid **2c** (yield 91%).

*d.* Alcohol **1c** (0.560 g, 2.11 mmol), TfOH (0.482 g, 3.21 mmol), and SbF_5_ (0.684 g, 3.16 mmol) (molar ratio 1.0:1.5:1.5), according to the typical procedure (Section 3.1) (21 h, r.t.), gave a mixture of compounds containing 20% of acid **2c**.

*e.* Alcohol **1c** (0.500 g, 1.88 mmol), FSO_3_H (0.363 g, 3.63 mmol), and SbF_5_ (0.782 g, 3.61 mmol) (molar ratio 1:2:2), according to the typical procedure (Section 3.1) (2 h, r.t.), after evaporation of the solvent and sublimation (120 °C, 1 Torr), afforded 0.490 g of acid **2c** (yield 89%).

*f.* Alcohol **1c** (0.250 g, 0.94 mmol), FSO_3_H (0.295 g, 2.95 mmol), and SbF_5_ (0.655 g, 3.02 mmol) (molar ratio 1:3:3), according to the typical procedure (Section 3.1) (7 h, 70 °C), gave a mixture of compounds containing 93% of acid **2c**; the content of **10c** did not exceed 2%.

*3,3,3-Trifluoro-2-(pentafluorophenyl)propanoic acid* (**2c**). White solid, mp 133.0–136.0 °C (CCl_4_). IR (KBr) ν, cm^−1^: 3427 (OH); 1749 (CO); 1489, 1471 (FAR). ^1^H NMR (CDCl_3_): *δ* 7.5 (br s, 1H, COOH), 4.76 (q, 1H, *J*_H,CF3_ = 8 Hz, CH). ^19^F NMR (CDCl_3_): *δ* −68.0 (dt, 3F, *J*_CF3,H_ = 8 Hz, *J*_CF3,F(ortho)_ = 8 Hz, CF_3_), −140 (m, 2F, F-*ortho*), −151.1 (tt, 1F, *J*_para,meta_ = 21 Hz, *J*_para,ortho_ = 3 Hz, F-*para*), −161.1 (m, 2F, F-*meta*). Anal. Calcd for C_9_H_2_F_8_O_2_: C, 36.76; H, 0.69; F, 51.68%. Found: C, 37.25; H, 0.42; F, 51.42%. HRMS, *m/z*: 293.9922 (M^+^). Calcd. M = 293.9922. 

### 3.5. Carbonylation of Perfluoro-1,1-diphenylethan-1-ol *(**1d**)*

*a.* Alcohol **1d** (0.618 g, 1.42 mmol), TfOH (0.422 g, 2.81 mmol), and SbF_5_ (0.610 g, 2.82 mmol) (molar ratio 1:2:2), according to the typical procedure (Section 3.1) (5 h, r.t. extraction with Et_2_O), gave a mixture of compounds containing 18% of **3d**, 12% of **4d**, and 65% of **5d**. 

*b.* A mixture of alcohol **1d** (0.517 g, 1.20 mmol), TfOH (0.360 g, 2.40 mmol), and SbF_5_ (0.519 g, 2.40 mmol) (molar ratio 1:2:2) was vigorously stirred in a 10 mL round-bottom glass flask (5 h, r.t.) in a slow flow of CO, then poured into 5 mL of MeOH. The resulting solution was kept at r.t. for 1 h, poured into 5% hydrochloric acid (30 mL), extracted with CH_2_Cl_2_ (3 × 4 mL), and the extract was dried over MgSO_4_. The mixture of compounds in the extract contained 8% of ester **2dMe**, 20% of **3d**, 2% of **4d**, and 64% of **5d.**

*c.* Alcohol **1d** (0.506 g, 1.17 mmol), TfOH (0.380 g, 2.53 mmol), and SbF_5_ (0.503 g, 2.32 mmol) (molar ratio 1:2:2), according to the typical procedure (Section 3.1) (24 h, r.t.), gave a mixture of compounds containing 21% of **2d**, 9% of **2dF**, 2% of **3d**, 1% of **4d**, and 60% of **5d** (1 h after extraction) or 17% of **2d**, 8% of **2dF**, 5% of **3d**, 3% of **4d**, and 60% of **5d** (72 h after extraction). After solvent evaporation, the residue was dissolved in Et_2_O (5 mL), then 5% hydrochloric acid (5 mL) was introduced, and the mixture was kept at r.t. for 24 h. The mixture of compounds (0.452 g) in the organic layer contained 22% of **3d**, 11% of **4d**, and 60% of **5d**. Silica gel column chromatography (hexane as the eluent) gave 12 mg of compound **3d**, 14 mg of alkene **4d**, and 0.405 g of mixed fractions containing compounds **3d**, **4d**, and **5d**.

*d.* Alcohol **1d** (0.349 g, 0.81 mmol), TfOH (0.484 g, 3.23 mmol), and SbF_5_ (0.700 g, 3.23 mmol) (molar ratio 1:4:4), according to the typical procedure (Section 3.1) (24 h, r.t. extraction with Et_2_O), afforded a mixture of compounds containing 21% of **1d**, 8% of **3d**, 4% of **4d**, and 63% of **5d**. 

*e.* Alcohol **1d** (0.322 g, 0.75 mmol), TfOH (0.135 g, 0.90 mmol), and SbF_5_ (0.194 g, 0.90 mmol) (molar ratio 1:1.2:1.2), according to the typical procedure (Section 3.1) (24 h, r.t. extraction with Et_2_O), gave a mixture of compounds containing 5% of **1d**, 6% of **3d**, 3% of **4d**, and 75% of **5d**. 

*f.* Alcohol **1d** (0.400 g, 0.93 mmol), FSO_3_H (0.182 g, 1.82 mmol), SbF_5_ (0.404 g, 1.86 mmol) (molar ratio 1:2:2), and C_6_F_6_ (0.5 mL), according to the typical procedure (Section 3.1) (3 h, r.t., extraction with Et_2_O), gave a mixture of compounds containing 1% of **1d**, 36% of **3d**, 8% of **4d**, and 51% of **5d**. After evaporation of Et_2_O, the residue was dissolved in CH_2_Cl_2_ (5 mL) and washed with a saturated aqueous solution of NaHCO_3_ (5 mL) to remove acidic impurities and dried over MgSO_4_. Evaporation of CH_2_Cl_2_ yielded 0.358 g of a mixture of compounds **1d**, **3d**, **4d**, and **5d**, to which NEt_3_ (0.200 g), CaH_2_ (0.360 g), and toluene (4 mL) were added. The resultant mixture was heated with stirring in a sealed glass ampoule at 125–130 °C for 12 h; then, the organic layer was filtered and treated with 5% hydrochloric acid (10 mL). The mixture of compounds (0.350 g) in the organic layer contained 30% of **4d** and 50% of **5d**. Silica gel column chromatography (hexane as eluent) gave 25 mg of alkene **4d** and 0.151 g of a mixed fraction containing compounds **4d** and **5d** at the 30:70 ratio.

*1,1,1-Trifluoro-2,2-bis(pentafluorophenyl)ethane (***3d***).* Colorless liquid. ^1^H NMR (CDCl_3_): *δ* 5.39 (q, 1H, *J*_H,CF3_ = 9.5 Hz, CH). ^19^F NMR (CDCl_3_): *δ* −67.2 (quintet d, 3F, *J*_CF3,F(ortho)_ = 11.5 Hz, *J*_CF3,H_ = 9.5 Hz, CF_3_), −139.9 (m, 4F, F-*ortho*), −151.8 (tt, 2F, *J*_para,meta_ = 21 Hz, *J*_para,ortho_ = 3 Hz, F-*para*), −161.2 (m, 4F, F-*meta*). HRMS, *m/z*: 415.9862 (M^+^). Calcd. for C_14_H_1_F_13_: M = 415.9865.

*Perfluoro-1,1-diphenylethene (***4d***).* White solid, mp 91.5–93.5 °C (hexane). IR (KBr) ν, cm^−1^: 1738 (C=CF_2_), 1525, 1504 (FAR). ^19^F NMR (CO(CD_3_)_2_): *δ* −75.4 (m, 2F, CF_2_), −139.2 (m, 4F, F-*ortho*), −152.3 (tt, 2F, *J*_para,meta_ = 20.5 Hz, *J*_para,ortho_ = 2.5 Hz, F-*para*), −161.7 (m, 4F, F-*meta*). HRMS *m/z*, 395.9808 (M^+^). Calcd. for C_14_F_12_: M = 395.9803.

*Perfluoro-2,2-diphenylpropionic acid (***2d***)* in the mixture with compounds **2dF**, **3d**, **4d**, and **5d**. ^19^F NMR (CH_2_Cl_2_-Et_2_O 3:1): *δ* −63.1 (quintet, 3F, *J*_CF3,F(ortho)_ = 19.5 Hz, CF_3_), −134.1 (m, 4F, F-*ortho*), −152.1 (tt, 2F, *J*_para,meta_ = 21.5 Hz, *J*_para,ortho_ = 5.5 Hz, F-*para*), −161.8 (m, 4F, F-*meta*). 

*Perfluoro-2,2-diphenylpropionyl fluoride (***2dF***)* in the mixture with compounds **2d**, **3d**, **4d**, and **5d**. ^19^F NMR (CH_2_Cl_2_-Et_2_O 3:1): *δ* 34.4 (octet, 1F, *J*_COF,F(ortho)_ = *J*_COF,CF3_ = 14.5 Hz, COF), −64.2 (quintet d, 3F, *J*_CF3,F(ortho)_ = 18.5 Hz, *J*_CF3,COF_ = 14.5 Hz, CF_3_), −134.3 (m, 4F, F-*ortho*), −148.8 (tt, 2F, *J*_para,meta_ = 21.5 Hz, *J*_para,ortho_ = 5.5 Hz, F-*para*), −159.6 (m, 4F, F-*meta*). 

*Methyl perfluoro-2,2-diphenylpropionate (***2dMe***)* in the mixture with compounds **3d**, **4d**, and **5d**. ^1^H NMR (CDCl_3_): *δ* 3.80 (s, 3H, CH_3_). ^19^F NMR (CDCl_3_): *δ* −61.8 (quintet, 3F, *J*_CF3,F(ortho)_ = 19.5 Hz, CF_3_), −134.8 (m, 4F, F-*ortho*), −151.3 (tt, 2F, *J*_para,meta_ = 21.5 Hz, *J*_para,ortho_ = 5 Hz, F-*para*), −161.3 (m, 4F, F-*meta*). GC-MS, *m/z*: 474 (M^+^). 

### 3.6. Carbonylation of Perfluoro-1,1-diphenylethane *(**5d**)*

Alcohol **1d** (0.510 g, 1.18 mmol), TfOH (0.343 g, 2.29 mmol), and SbF_5_ (0.510 g, 2.35 mmol) (molar ratio 1:2:2), according to the typical procedure (Section 3.1) (24 h, r.t.), gave a mixture of compounds containing 9% of **1d**, 9% of **2dF**, and 81% of **5d**; levels of **3d** and **4d** were >1%.

### 3.7. Carbonylation of Perfluoro-1-phenylindan-1-ol *(**1e**)*

Alcohol **1e** (0.350 g, 0.79 mmol), FSO_3_H (0.155 g, 1.55 mmol), SbF_5_ (0.345 g, 1.59 mmol) (molar ratio 1:2:2), and C_6_F_6_ (0.5 mL), according to the typical procedure (Section 3.1) (3 h, r.t., extraction with Et_2_O), after evaporation of the solvent, afforded a mixture of compounds (0.332 g) containing 35% of **1e**, 47% of **3e**, 10% of **4e**, and 4% of **5e**.

### 3.8. Carbonylation of Perfluoro-1-phenyltetralin-1-ol *(**1f**)*

*a.* Alcohol **1f** (0.350 g, 0.71 mmol), FSO_3_H (0.139 g, 1.39 mmol), SbF_5_ (0.310 g, 1.43 mmol) (molar ratio 1:2:2), and C_6_F_6_ (0.5 mL), according to the typical procedure (Section 3.1) (3 h, r.t., extraction with Et_2_O), after evaporation of the solvent, gave a mixture of compounds (0.336 g) containing 13% of **1f**, 8% of **3f**, 14% of **4f**, 41% of **5f**, and 20% of lactone **8**. 2,2,3,3,4,4,5,6,7,8-decafluoro-1-(pentafluorophenyl)tetralin (**3f**) was detected in the reaction mixture by GC-MS (M = 478) and ^1^H NMR (CDCl_3_): *δ* 5.25 (dd, *J*_H,F(2)_ = 17 and 10 Hz, H-1).

*b.* Alcohol **1f** (0.600 g, 1.21 mmol), FSO_3_H (0.477 g, 4.77 mmol), SbF_5_ (1.062 g, 4.90 mmol) (molar ratio 1:4:4), and C_6_F_6_ (1 mL), according to the typical procedure (Section 3.1) (10 h, 50 °C, extraction with CH_2_Cl_2_), after evaporation of the solvent, gave a mixture of compounds (0.580 g) containing 15% of **1f**, 1% of **3f**, 2% of **4f**, 27% of **5f**, and 47% of lactone **8**. Silica gel column chromatography (CCl_4_ as the eluent) and recrystallization from hexane afforded 0.112 g of lactone **8** (yield 32%).

*Perfluoro-4-phenyl-1,4-(epoxymethano)tetralin-9-one (***8***).* White solid, mp 86–87.5 °C (hexane). IR (KBr) ν, cm^−1^: 1821 (C=O), 1537, 1520, 1508, 1493 (FAR). ^19^F NMR (CDCl_3_): *δ* −111.5 (dddd, 1F, *J*_A3,B3_ = 237 Hz, *J*_A3,ortho-2_ = 64.2 Hz, *J*_A3,B2_ = 12.3 Hz, *J*_A3,F1_ = 4.2 Hz, F_A_-3), −115.4 (dddd, 1F, *J*_B3,A3_ = 237 Hz, *J*_B3,ortho-2_ = 69.3 Hz, *J*_B3,A2_ = 9.3 Hz, *J*_B3,1_ = 5.5 Hz, F_B_-3), −122.8 (ddd, 1F, *J*_A2,B2_ = 230 Hz, *J*_A2,1_ = 16.3 Hz, *J*_A2,B3_ = 9.2 Hz, F_A_-2), −130.4 (ddq, 1F, *J*_ortho,meta_ = 22.5 Hz, *J* = 10 Hz, *J* = 5 Hz, F-ortho-1), −133.8 (ddm, 1F, *J*_ortho-2,B3_ = 69.3 Hz, *J*_ortho-2,A3_ = 64.2 Hz, F-ortho-2), −134.9 (ddd, 1F, *J*_B2,A2_ = 230 Hz, *J*_B2,A3_ = 12.3 Hz, *J*_B2,1_ = 12.1 Hz, F_B_-2), −139.6 (ddddd, 1F, *J*_5,6_ = 20 Hz, *J*_5,8_ = 15.2 Hz, *J*_5,7_ = 8.6 Hz, *J*_5,1_ = 5 Hz, *J*_5,ortho_ = 5 Hz, F-5), −140.5 (dddd, 1F, *J*_8,1_ = 43.4 Hz, *J*_8,7_ = 21.6 Hz, *J*_8,5_ = 15.2 Hz, *J*_8,6_ = 8.2 Hz, F-8), −144.6 (ddd, 1F, *J*_6,7_ = 20.2 Hz, *J*_6,5_ = 20 Hz, *J*_6,8_ = 8.2 Hz, F-6), −146.5 (ddd, 1F, *J*_7,8_ = 21.2 Hz, *J*_7,6_ = 20.2 Hz, *J*_7,5_ = 8.6 Hz, F-7), −149.3 (tt, 1F, *J*_para,ortho_ = 21.5 Hz, *J*_para,meta_ = 5.5 Hz, F-para), −159.2 (dddddd, 1F, *J*_1,8_ = 43.4 Hz, *J*_1,A2_ = 16.3 Hz, *J*_1,B2_ = 12.1 Hz, *J*_1,B3_ = 5.5 Hz, *J*_1,5_ = 5 Hz, *J*_1,A3_ = 4.2 Hz, F-1), −159.8 (ddd, 1F, *J*_meta,ortho_ = 21.3 Hz, *J*_meta,para_ = 21.3 Hz, *J* = 5.6 Hz, F-meta-1), −161.3 (ddd, 1F, *J*_meta,ortho_ = 22.1 Hz, *J*_meta,para_ = 22.1 Hz, *J* = 7.6 Hz, F-meta-2). ^13^C NMR (CDCl_3_): *δ* 155.8 (dd, ^3^*J*_CF_ = 10 Hz, ^3^*J*_CF_ = 5.5 Hz, C=O), 141.4–147.8 (components of seven signals, dm, ^1^*J*_CF_ ≈ 250–260 Hz, CF^Ar^), 138.6 (dm, ^1^*J*_CF_ = 252.5, CF^Ar^), 137.5 (dm, ^1^*J*_CF_ = 253.5, CF^Ar^), 111.4 (dd, ^2^*J*_CF_ = 22, ^2^*J*_CF_ = 12, C^Ar^), 110.5 (m, C^Ar^), 110.2 (ttd, ^1^*J*_CF_ = 282, ^2^*J*_CF_ = 22, ^3^*J*_CF_ = 6, C-3), 109.4 (ddtd, ^1^*J*_CF_ = 282, ^1^*J*_CF_ = 272, ^2^*J*_CF_ = 23.5, ^2^*J*_CF_ = 20.5, C-2), 104.9 (dt, ^1^*J*_CF_ = 256, ^2^*J*_CF_ = 26, C-1), 102.4 (t, ^2^*J*_CF_ = 14, C^Ar^), 57.5 (t, ^2^*J*_CF_ = 25, C-4). Anal. Calcd for C_17_F_14_O_2_: C, 40.66; H, 0.00; F, 52.97%. Found: C, 41.28; H, 0.22; F, 52.95%. HRMS *m/z*, 501.9665 (M^+^). Calcd: M = 501.9669.

### 3.9. Carbonylation of 2,2,3,3,3-Pentafluoro-1-(pentafluorophenyl)propan-1-ol *(**1g**)*

*a.* Alcohol **1g** (0.442 g, 1.40 mmol), TfOH (0.460 g, 3.07 mmol), and SbF_5_ (0.606 g, 2.80 mmol) (molar ratio 1:2:2), according to the typical procedure (Section 3.1) (2 h, r.t.), gave a mixture of acids **2g** and **10g** in the 98:2 ratio. Evaporation of the solvent and sublimation (95 °C, 3 Torr) afforded 0.423 g of acid **2g** (yield 88%).

*b.* Alcohol **1g** (0.192 g, 0.61 mmol), TfOH (0.294 g, 1.96 mmol), and SbF_5_ (0.410 g, 1.89 mmol) (molar ratio 1:3:3), according to the typical procedure (Section 3.1) (5.5 h, 70 °C), gave a mixture of compounds containing 42% of **2g** and 52% of **10g** (*E:Z* = 18:82).

*c.* Alcohol **1g** (0.205 g, 0.65 mmol), FSO_3_H (0.214 g, 2.14 mmol), and SbF_5_ (0.473 g, 2.18 mmol) (molar ratio 1:3:3), according to the typical procedure (Section 3.1) (5.5 h, 70 °C) gave a mixture of compounds containing 13% of **2g** and 80% of **10g** (E:Z = 15:85). Evaporation of the solvent and sublimation (95 °C, 3 Torr) resulted in a mixture (0.154 g, yield 73%) of acids **2g** and **10g** (*E:Z* = 20:80) at the 16:84 ratio. The mixture was treated with an Et_2_O solution of CH_2_N_2_. Evaporation of the solvent gave a mixture (0.152 g) of esters **2gMe** and **10gMe** (*E:Z* = 25:75) in the 16:84 ratio.

The mixture of esters **2gMe** and **10gMe** (0.100 g) was dissolved in dry CHCl_3_ (3 mL), then NEt_3_ (0.3 mL) was added. The mixture was kept at r.t. for 1 h and washed with 5% hydrochloric acid (2 × 7 mL). Silica gel column chromatography (CCl_4_–CHCl_3_ [1:1] as the eluent) gave 80 mg (yield 80%) of compound **10gMe** (*E:Z* = 46:54).

*3,3,4,4,4-Pentafluoro-2-(pentafluorophenyl)butanoic acid (***2g***)*. White solid, mp 74.5–75.2 °C (CCl_4_). IR (KBr) ν, cm^−1^: 1751 (C=O), 1531, 1510 (FAR). ^1^H NMR (CDCl_3_): *δ* 10.2 (br.s, 1H, COOH), 4.81 (dd, 1H, *J*_H,F(B3)_ = 16.4 Hz, *J*_H,F(A3)_ = 12.4 Hz, CH). ^19^F NMR (CDCl_3_): *δ* −83.4 (t, 3F, *J*_CF3,F(ortho)_ = 2.3 Hz, CF_3_), −114.4 (ddt, 1F, *J*_A3,B3_ = 276 Hz, *J*_F(A3),H_ = 12.4 Hz, *J*_A3,ortho_ = 7.7 Hz, F_A_-3), −117.5 (dq, 1F, *J*_A3,B3_ = 276 Hz, *J*_F(B3),H_ = *J*_B3,ortho_ = 16.4 Hz, F_B_-3), −139.3 (br.s, 2F, F-ortho), −150.6 (tt, 1F, *J*_para,meta_ = 21 Hz, *J*_para,ortho_ = 3.3 Hz, F-para), −161.0 (m, 2F, F-meta). Anal. Calcd for C_10_H_2_F_10_O_2_: C, 34.90; H, 0.59; F, 55.21%. Found: C, 34.75; H, 0.56; F, 55.04%. HRMS m/z, 343.9895 (M^+^). Calcd. M = 343.9890.

*Methyl 3,3,4,4,4-pentafluoro-2-(pentafluorophenyl)butanoate (***2gMe***)* in the mixture with **10gMe** (**2gMe**:**10gMe** = 16:84). ^1^H NMR (CDCl_3_): *δ* 4.74 (dd, 1H, *J*_H,F(B3)_ = 16 Hz, *J*_H,F(A3)_ = 13 Hz, CH), 3.81 (s, 3H, CH_3_). ^19^F NMR (CDCl_3_): *δ* −83.4 (t, 3F, *J*_CF3,F(ortho)_ = 2.3 Hz, CF_3_), −114.6 (ddt, 1F, *J*_A3,B3_ = 275 Hz, *J*_F(A3),H_ = 13 Hz, *J*_A3,ortho_ = 8.2 Hz, F_A_-3), −117.2 (ddt, 1F, *J*_A3,B3_ = 275 Hz, *J*_F(B3),H_ = 16 Hz, *J*_B3,ortho_ = 16 Hz, F_B_-3), −139.6 (br.s, 2F, F-*ortho*), −151.5 (tt, 1F, *J_para,meta_* = 21.0 Hz, *J_para,ortho_* = 3.1 Hz, F-*para*), −161.4 (m, 2F, F-*meta*). HRMS m/z, 358.0038 (M^+^). Calcd. for C_11_H_4_F_10_O_2_: M = 358.0046.

*Perfluoro-2-phenylbut-2-enoic acid (***10g***)* (*E*:*Z* =20:80) in the mixture with **2g** (**2g**:**10g** = 16:84)*. (E)*-**10g**: ^1^H NMR (CDCl_3_): *δ* 9.94 (s, COOH). ^19^F NMR (CDCl_3_): *δ* −67.6 (d, 3F, *J*_CF3,F(3)_ = 6.3 Hz, CF_3_), −100.1 (tq, 1F, *J*_3,*ortho*_ = 11 Hz, *J*_F(3),CF3_ = 6.3 Hz, F-3), −137.9 (m, 2F, F-*ortho*), −150.3 (tt, 1F, *J_para,meta_* = 21 Hz, *J_para,ortho_* = 2.5 Hz, F-*para*), −161.2 (m, 2F, F-*meta*). *(Z)*-**10g**: ^1^H NMR (CDCl_3_): *δ* 9.94 (s, COOH). ^19^F NMR (CDCl_3_): *δ* −70.3 (dt, 3F, *J*_CF3,F(3)_ = 7.3 Hz, *J*_CF3,F(ortho)_ = 2 Hz, CF_3_), −97.5 (qt, 1F, *J*_F(3),CF3_ = 7.3 Hz, *J*_3,ortho_ = 2 Hz, F-3), −139.2 (m, 2F, F-*ortho*), −150.4 (tt, 1F, *J_para,meta_* = 21 Hz, *J_para,ortho_* = 2.5 Hz, F-*para*), −161.5 (m, 2F, F-*meta*).

*Methyl perfluoro-2-phenylbut-2-enoate (***10gMe***)* (*E:Z* = 46:54). Colorless liquid. IR (film) ν, cm^−1^: 2962 (CH), 1753 (C=O), 1523, 1506 (FAR). *(E)*-**10g**: ^1^H NMR (CDCl_3_): *δ* 3.85 (s, 3H, CH_3_). ^19^F NMR (CDCl_3_): *δ* −68.3 (d, 3F, *J*_CF3,F(3)_ = 7.0 Hz, CF_3_), −106.4 (tqd, 1F, *J*_3,*ortho*_ = 12.3 Hz, *J*_F(3),CF3_ = 7 Hz, *J*_F3,*para*_ = 1.2 Hz, F-3), −138.0 (m, 2F, F-*ortho*), −150.9 (ttd, 1F, *J_para,meta_* = 21 Hz, *J_para,orth_*_o_ = 3.5 Hz, *J_para,_*_F3_ = 1.2 Hz, F-*para*), −161.5 (m, 2F, F-*meta*). *(Z)*-**10g**: ^1^H NMR (CDCl_3_): *δ* 3.85 (s, 3H, CH_3_). ^19^F NMR (CDCl_3_): *δ* −70.3 (dt, 3F, *J*_CF3,F(3)_ = 7.7 Hz, *J*_CF3,F(ortho)_ = 2.1 Hz, CF_3_), −102.6 (qt, 1F, *J*_F(3),CF3_ = 7.7 Hz, *J*_3,ortho_ = 2 Hz, F-3), −139.4 (m, 2F, F-*ortho*), −151.2 (tt, 1F, *J_para,meta_* = 21 Hz *J_para,ortho_* = 2.5 Hz, F-*para*), −161.9 (m, 2F, F-*meta*). HRMS m/z, 337.9986 (M^+^). Calcd. for C_11_F_9_H_3_O_2_: M= 337.9984.

### 3.10. Carbonylation of 2,2,3,3,4,4,5,6,7,8-Decafluorotetralin-1-ol *(**1h**)*

*a.* Alcohol **1h** (0.417 g, 1.28 mmol), FSO_3_H (0.260 g, 2.60 mmol), and SbF_5_ (0.580 g, 2.68 mmol) (molar ratio 1:2:2), according to the typical procedure (Section 3.1) (2 h, r.t.), gave a mixture of compounds containing 69% of **2h** and 23% of **10h**. Evaporation of the solvent, sublimation (110 °C, 1 Torr), and recrystallization from CCl_4_ resulted in 0.150 g of acid **2h** (yield 35%).

*b.* Alcohol **1h** (0.310 g, 0.95 mmol), FSO_3_H (0.201 g, 2.01 mmol), and SbF_5_ (0.439 g, 2.03 mmol) (molar ratio 1:2:2), according to the typical procedure (Section 3.1) (24 h, r.t.), gave a mixture of compounds containing 61% of **2h** and 25% of **10h**. Evaporation of the solvent and sublimation (110 °C, 1 Torr) afforded a mixture (0.268 g, yield 80%) containing >95% of acids **2h** and **10h** in the 75:25 ratio. The mixture was treated with an Et_2_O solution of CH_2_N_2_ to produce a mixture of esters **2hMe** and **10hMe** at the same ratio. Silica gel column chromatography (CCl_4_ as eluents) gave 0.197 mg (yield 59%) of compound **10hMe**.

*c.* Alcohol **1h** (0.560 g, 1.71 mmol), FSO_3_H (0.690 g, 6.90 mmol), and SbF_5_ (1.470 g, 6.79 mmol) (molar ratio 1:4:4), according to the typical procedure (Section 3.1) (20 h, r.t.), after evaporation of the solvent and sublimation (120 °C, 3 Torr), gave a mixture of compounds (0.500 g) containing 48% of **2h** and 45% of **10h**. 

*d.* Alcohol **1h** (0.400 g, 1.22 mmol), FSO_3_H (0.368 g, 3.68 mmol), and SbF_5_ (0.782 g, 3.61 mmol) (molar ratio 1:3:3), according to the typical procedure (Section 3.1) (11 h, 70 °C), gave a mixture of compounds **2h**, **10h**, **11**, **12**, and **13** in the 9:20:45:11:15 ratio. The extract was washed with a saturated aqueous solution of NaHCO_3_ (20 mL). The organic layer was dried over MgSO_4_ and evaporated to obtain 76 mg of a mixture of compounds containing (according to GC-MS) 41% of tetralin **12** and 51% of tetralon **13**. The aqueous solution was acidified with HCl (pH < 1) and extracted with Et_2_O (3 × 4 mL), and the solvent was evaporated to obtain 0.248 g of a mixture of acids **2h**, **10h**, and **11** in the 11:29:60 ratio. The mixture was treated with an Et_2_O solution of CH_2_N_2_. Silica gel column chromatography (first CCl_4_ and then CCl_4_–CHCl_3_ [1:1] as an eluent) resulted in 83 mg of compound **11Me** (yield 21%).

*2,2,3,3,4,4,5,6,7,8-decafluorotetralin-1-carboxylic acid (***2h***).* White solid, mp 99.2–101.5 °C (CCl_4_). IR (KBr) ν, cm^−1^: 2935 (CH), 1740 (C=O), 1533, 1500 (FAR). ^1^H NMR (CDCl_3_): *δ* 7.99 (br.s, 1H, COOH), 4.67 (t, 1H, *J*_H,F(2)_ = 11.0 Hz, CH). ^19^F NMR (CDCl_3_): *δ* −106.3 (dm, 1F, *J*_A4,B4_ = 291 Hz, F_A_-4), −109.7 (dm, 1F, *J*_A4,B4_ = 291 Hz, F_B_-4), −114.0 (dm, 1F, *J*_A2,B2_ = 271 Hz, F_A_-2), −123.0 (dm, 1F, *J*_A2,B2_ = 271 Hz, F_B_-2), −135.1 (dm, 1F, *J*_A3,B3_ = 270 Hz, F_A_-3), −136.7 (dddm, 1F, *J*_8,7_ = 20.8 Hz, *J*_8,5_ = 11.6 Hz, *J*_8,6_ = 5.6 Hz, F-8), −137.2 (dtdd, 1F, *J*_5,6_ = 20.8 Hz, *J*_5,4_ = 18.7 Hz, *J*_5,8_ = 11.6 Hz, *J*_5,7_ = 8.2 Hz, F-5), −140.1 (dm, 1F, 1F, *J*_A3,B3_ = 270 Hz, F_B_-3), −147.3 (dddt, 1F, *J*_7,6_ = 20.8 Hz, *J*_7,8_ = 20.8 Hz, *J*_7,5_ = 8.2 Hz, *J*_7,4_ = 2 Hz, F-7), −150.5 (dddt, 1F, *J*_6,5_ = 20.8 Hz, *J*_6,7_ = 20.8 Hz, *J*_6,8_ = 5.6 Hz, *J* = 1 Hz, F-6). Anal. Calcd for C_11_H_2_F_10_O_2_: C, 37.10; H, 0.57; F, 53.35%. Found: C, 36.70; H, 0.62; F, 53.43%. HRMS *m/z*, 355.9893 (M^+^). Calcd. M = 355.9890.

*Methyl 2,2,3,3,4,4,5,6,7,8-decafluorotetralin-1-carboxylate (***2hMe***)* in the mixture with acid **2hMe** (**2hMe**:**10Me** = 75:25). ^19^F NMR (Et_2_O-CDCl_3_ 4:1): *δ* −106.2 (dm, 1F, *J*_A4,B4_ = 291 Hz, F_A_-4), −108.3 (dm, 1F, *J*_A4,B4_ = 291 Hz, F_B_-4), −113.8 (dm, 1F, *J*_A2,B2_ = 271 Hz, F_A_-2), −122.3 (dm, 1F, *J*_A2,B2_ = 271 Hz, F_B_-2), −135.4 (dm, 1F, *J*_A3,B3_ = 270 Hz, F_A_-3), −136.4 (m, 1F, F-8), −137.8 (m, 1F, F-5), −138.9 (dm, 1F, 1F, *J*_A3,B3_ = 270 Hz, F_B_-3), −148 (m, 1F, F-7), −151.3 (m, 1F, F-6).

*Perfluoro-3,4-dihydronaphthalene-1-carboxylic acid (***10h***)* in the mixture with acid **2h** (**2h**:**10h** = 75:25). ^19^F NMR (CDCl_3_): *δ* −119.8 (dm, 2F, *J*_4,5_ = 32.6 Hz, F-4), −121.0 (m, 1F, F-2), −130.3 (m, 2F, F-3), −133.6 (m, 1F, F-8), −136.6 (tm, 1F, *J*_5,4_ = 32.6 Hz, F-5), −146.9 (m, 1F, F-7), −149.4 (m, 1F, F-6).

*Methyl perfluoro-3,4-dihydronaphthalene-1-carboxylate (***10hMe***).* Colorless liquid. IR (film) ν, cm^−1^: 2962 (CH), 1753 (C=O), 1520, 1497 (FAR). ^1^H NMR (CDCl_3_): *δ* 3.95 (s, 3H, CH_3_). ^19^F NMR (CDCl_3_): *δ* −120.6 (dm, 2F, *J*_4,5_ = 32.6 Hz, F-4), −123.9 (tdm, 1F, *J*_2,3_ = 18.5 Hz, *J*_2,8_ = 7 Hz, *J*_2,6_ = 6.3 Hz, F-2), −131.0 (dm, 2F, *J*_3,2_ = 18.5 Hz, F-3), −136.3 (ddddm, 1F, *J*_8,7_ = 20 Hz, *J*_8,5_ = 11.5 Hz, *J*_8,2_ = 7 Hz, *J*_8,6_ = 6.3 Hz, F-8), −137 (tdddd, 1F, *J*_5,4_ = 32.6 Hz, *J*_5,6_ = 21 Hz, *J*_5,8_ = 11.5 Hz, *J*_5,7_ = 8.9 Hz, *J*_5,2_ = 2 Hz, F-5), −147.8 (dddm, 1F, *J*_7,6_ = 20 Hz, *J*_7,8_ = 20 Hz, *J*_7,5_ = 8.7 Hz, F-7), −150.3 (dddd, 1F, *J*_6,5_ = 21 Hz, *J*_6,7_ = 20 Hz, *J*_6,8_ = 6.3 Hz, *J*_6,2_ = 6.3 Hz, F-6). HRMS *m/z*, 349.9979 (M^+^). Calcd. for C_12_H_3_F_9_O_2_: M = 349.9984.

*2,3,5,6,7,8-hexafluoro-4-hydroxy-1-naphthoic (***11***)* in the mixture with acids **2h** and **10h** (**11**:**2h**:**10h** = 60:11:29). ^19^F NMR (Et_2_O-CDCl_3_ 4:1): *δ* −135.4 (dm, 1F, *J*_2,3_ = 21.6 Hz, F-2), −142.7 (m, 1F, F-5 or F-8), −142.9 (m, 1F, F-5 or F-8), −157.6 (m, 1F, F-6 or F-7), −160.5 (m, 1F, F-6 or F-7), −161.8 (dm, 1F, *J*_3,2_ = 21.6 Hz, F-3).

*Methyl 2,3,5,6,7,8-hexafluoro-4-methoxy-1-naphthoate (***11Me***).* White solid, mp 43.8–45.0 °C. IR (film) ν, cm^−1^: 2960 (CH), 1747 (C=O), 1529, 1506 (FAR). ^1^H NMR (CDCl_3_): *δ* 4.12 (d, 3H, *J*_CH3,F(3)_ = 2 Hz, OCH_3_), 4.00 (s, 3H, COOCH_3_). ^19^F NMR (CDCl_3_): *δ* −133.6 (ddddd, 1F, *J*_2,3_ = 20.3 Hz, *J*_2,6_ = 7.9 Hz, *J*_2,8_ = 5 Hz, *J*_2,5_ = 4 Hz, *J*_2,7_ = 1.4 Hz, F-2), −143.7 (ddddd, *J*_8,7_ = 18.6 Hz, *J*_8,5_ = 14.8 Hz, *J*_8,2_ = 5 Hz, *J*_8,3_ = 4 Hz, *J*_8,6_ = 1.9 Hz, 1F, F-8), −146.1 (ddddd, 1F, *J*_5,6_ = 17.8 Hz, *J*_5,8_ = 14.5 Hz, *J*_5,3_ = 6 Hz, *J*_5,2_ = 4 Hz, *J*_5,7_ = 3.2 Hz, F-5), −152.9 (dddddq, 1F, *J*_3,2_ = 20.3 Hz, *J*_3,7_ = 8.4 Hz, *J*_3,5_ = 6 Hz, *J*_3,8_ = 4 Hz, *J*_3,6_ = 2.9 Hz, *J*_F(3),CH3_ = 2 Hz, F-3), −155.8 (ddddd, 1F, *J*_7,6_ = 20.3 Hz, *J*_7,8_ = 18.6 Hz, *J*_7,3_ = 8.4 Hz, *J*_7,5_ = 3.2 Hz, *J*_7.2_ = 1.4 Hz, F-7), −157.4 (ddddd, 1F, *J*_6,7_ = 20.3 Hz, *J*_6,5_ = 17.8 Hz, *J*_6,2_ = 7.9 Hz, *J*_6,3_ = 2.9 Hz, *J*_6,8_ = 1.9 Hz, F-6). HRMS *m/z*, 324.0214 (M^+^). Calcd. for C_13_H_6_F_6_O_3_: M = 324.0216.

*3,3,4,4,5,6,7,8-Octafluorotetralin-2-one (***13***)* in the mixture with tetralin **12**. ^1^H NMR (CDCl_3_): *δ* 4.00 (s, 2H, H-1). ^19^F NMR (CDCl_3_): *δ* −109.3 (dt, 2F, *J*_4,5_ = 28 Hz, *J*_4,3_ = 12 Hz, F-4), −133.6 (t, 2F, *J*_3,4_ = 12 Hz, F-3), −138.3 (tddd, 1F, *J*_5,4_ = 28 Hz, *J*_5,6_ = 20.5 Hz, *J*_5,8_ = 12.7 Hz, *J*_5,7_ = 8.0 Hz, F-5), −138.8 (dddt, 1F, *J*_8,7_ = 21 Hz, *J*_8,5_ = 12.7 Hz, *J*_8,6_ = 4 Hz, *J* = 2 Hz, F-8), −148.0 (dddt, 1F, *J*_7,8_ = 21 Hz, *J*_7,6_ = 20 Hz, *J*_7,5_ = 8 Hz, *J*_7,4_ = 2.5 Hz, F-7), −153.3 (dddm, 1F, *J*_6,5_ = 20.5 Hz, *J*_6,7_ = 20 Hz, *J*_6,8_ = 4 Hz, F-6). GC-MS, *m/z*: 290 (M^+^).

### 3.11. Carbonylation of 2,2,3,3,4,5,6,7-Octafluoroindan-1-ol *(**1i**)*

*a.* Alcohol **1i** (0.405 g, 1.47 mmol), FSO_3_H (0.287 g, 2.87 mmol), and SbF_5_ (0.580 g, 2.87 mmol) (molar ratio 1:2:2), according to the typical procedure (Section 3.1) (2.5 h, r.t.), gave a mixture of compounds containing (judging by ^19^F NMR and GC-MS) 50% of **2i**, 5% of **10i**, 20% of 2,2,3,4,5,6,7-heptafluoroindan-1-one (M = 258), and 50% of 2,2,4,5,6,7-hexafluoro-3-oxoindan-1-yl sulfurofluoridate (M = 338).

*b.* Alcohol **1i** (0.413 g, 1.49 mmol), FSO_3_H (0.452 g, 4.52 mmol), and SbF_5_ (0.984 g, 4.55 mmol) (molar ratio 1:3:3), according to the typical procedure (Section 3.1) (6 h, 70 °C), gave a mixture of compounds containing 63% of **2j** and 34% of **10j**. The solvent was evaporated, and the residue was dissolved in Et_2_O (10 mL), after which the solution was saturated with gaseous ammonia until precipitation of yellow crystals stopped. Crystals of an ammonium salt of acid **10j** were filtered off and washed with Et_2_O (5 mL). Next, 5% hydrochloric acid (5 mL) and Et_2_O (5 mL) were added to the crystals, the organic layer was separated and dried over MgSO_4_; evaporation and recrystallization from CCl_4_–acetone gave 0.100 g (yield 25%) of acid **10j**. The ether solution obtained after the filtration of crystals of the ammonium salt of acid **10j** was washed with 5% hydrochloric acid (10 mL) and dried over MgSO_4**.**_ Evaporation of the solvent and sublimation (130 °C, 1 Torr) afforded 0.170 g of acid **2j** (yield 40%).

*2,2,4,5,6,7-Hexafluoro-3-oxoindan-1-carboxylic acid (***2j***).* White solid, mp 136.2–137.0 °C (CCl_4_ + CH_2_Cl_2_). IR (KBr) ν, cm^−1^: 2966 (CH), 1794 (C=O), 1525, 1504 (FAR). ^1^H NMR (CDCl_3_): *δ* 7.85 (br.s, 1H, COOH), 4.64 (dd, 1H, *J*_H,F(A2)_ = 15.7 Hz, *J*_H,F(B2)_ = 5 Hz, CH). ^19^F NMR (CDCl_3_): *δ* −107.3 (dd, 1F, *J*_A2,B2_ = 284 Hz, *J*_F(A2),H_ = 15.7 Hz, F_A_-2), −116.4 (dd, 1F, *J*_A2,B2_ = 284 Hz, *J*_F(B2),H_ = 5 Hz, F_B_-2), −134.5 (ddd, 1F, *J*_4,5_ = 20.5 Hz, *J*_4,7_ = 17.9 Hz, *J*_4,6_ = 12 Hz, F-4), −138.1 (ddd, 1F, *J*_6,7_ = 20 Hz, *J*_6,5_ = 19 Hz, *J*_6,4_ = 12 Hz, F-6), −138.6 (ddd, 1F, *J*_7,6_ = 20.5 Hz, *J*_7,4_ = 17.9 Hz, *J*_7,5_ = 4.5 Hz, F-7), −149.8 (dddd, 1F, *J*_5,4_ = 20.5 Hz, *J*_5,6_ = 19 Hz, *J*_5,7_ = 4.5 Hz, *J*_F(5)-H_ = 2.1 Hz, F-5). Anal. Calcd for C_10_H_2_F_6_O_3_: C, 42.28; H, 0.71; F, 40.12%. Found: C, 42.06; H, 0.83; F, 40.24%.). HRMS *m/z*, 283.9900 (M^+^). Calcd. M = 283.9903.

*Perfluoro-1-oxo-1H-indene-3-carboxylic acid (***10j***).* Yellow solid, mp 183.0–185.0 °C (with decomposition) (CCl_4_ + acetone). IR (KBr) ν, cm^−1^: 1751, 1736 (C=O), 1506, 1485 (FAR). ^1^H NMR (CO(CD_3_)_2_): *δ* 3.8 (br.s, 1H, COOH). ^19^F NMR (CO(CD_3_)_2_): *δ* −128.1 (dddd, 1F, *J*_2,6_ = 14.8 Hz, *J*_2,4_ = 8.5 Hz, *J*_2,5_ = 2.6 Hz, *J*_2,7_ = 2.6 Hz, F-2), −134.8 (dddd, 1F, *J*_4,5_ = 19.1 Hz, *J*_4,7_ = 13.2 Hz, *J*_4,2_ = 8.5 Hz, *J*_4,6_ = 4 Hz, F-4), −136.0 (dddd, 1F, *J*_7,6_ = 20.5 Hz, *J*_7,4_ = 13.2 Hz, *J*_7,5_ = 10.2 Hz, *J*_7,2_ = 2.6 Hz, F-7), −143.7 (dddd, 1F, *J*_5,4_ = 19.1 Hz, *J*_5,6_ = 16 Hz, *J*_5,7_ = 10.2 Hz, *J*_5,2_ = 2.6 Hz, F-5), −153.5 (dddd, 1F, *J*_6,7_ = 20.5 Hz, *J*_6,5_ = 16 Hz, *J*_6,2_ = 14.8 Hz, *J*_6,4_ = 2.6 Hz, F-6). Anal. Calcd for C_10_H_1_F_5_O_3_: C, 45.48; H, 0.38; F, 35.97%. Found: C, 44.89; H, 0.57; F, 35.77%. HRMS *m/z*, 263.9845 (M^+^). Calcd. M = 263.9840.

### 3.12. Carbonylation of 2,2,4,5,6,7-Hexafluoro-3-Hydroxyindan-1-one *(**1j**)*

Alcohol **1j** (0.192 g, 1.80 mmol), FSO_3_H (0.618 g, 6.18 mmol), and SbF_5_ (1.345 g, 6.21 mmol) (molar ratio 1.0:3.5:3.5), according to the typical procedure (Section 3.1) (9.5 h, 70 °C), gave a mixture of compounds containing 35% of **2j** and 55% of **10j**.

### 3.13. Carbonylation of 2,2,3,4,5,6,7-Heptafluoro-3-(Pentafluoroethyl)indan-1-ol *(**1k**)*

Alcohol **1k** (0.340 g, 0.90 mmol), FSO_3_H (0.18 g, 1.80 mmol), and SbF_5_ (0.390 g, 1.80 mmol) (molar ratio 1:2:2), according to the typical procedure (Section 3.1) (2.5 h, r.t.), after evaporation of the solvent and sublimation (120 °C, 1 Torr), gave 0.300 g of acid **2k** (yield 82%).

*2,2,3,4,5,6,7-heptafluoro-3-(pentafluoroethyl)indan-1-carboxylic acid (***2k***)* (isomers A:B = 67:33). Colorless liquid. IR (film) ν, cm^−1^: 3033 (OH), 2943 (CH), 1741 (CO), 1516 (FAR). *Isomer A.* ^1^H NMR (CDCl_3_): *δ* 10.48 (br.s, 1H, COOH), 4.61 (tm, 1H, *J*_H,F(2)_ ~ 12 Hz, CH). ^19^F NMR (CDCl_3_): *δ* −80.8 (m, 3F, CF_3_), −110.8 (dm, 1F, *J*_A2,B2_ = 242 Hz, F_A_-2), −113.5 (dm, 1F, *J*_A2,B2_ = 242 Hz, F_B_-2), −119.6 (dm, 1F, *J*_A,B_ = 297 Hz, F_A_-CF_2_CF_3_), −122.1 (dm, 1F, *J*_A,B_ = 297 Hz, F_B_-CF_2_CF_3_), −135.7 (m, 1F, F-4), −136.6 (ddd, 1F, *J*_7,6_ = 20.6 Hz, *J*_7,4_ = 16.1 Hz, *J*_7,5_ = 5.6 Hz, F-7), −145.6 (dddd, 1F, *J*_6,7_ = 20.6 Hz, *J*_6,5_ = 19.4 Hz, *J*_6,4_ = 8.7 Hz, *J* = 3.3 Hz, F-6), −150.1 (dddm, 1F, *J*_5,4_ = 20.6 Hz, *J*_5,6_ = 19.4 Hz, *J*_5,7_ = 5.6 Hz, F-5), −175.2 (br.s, 1F, F-3). *Isomer B.* ^1^H NMR (CDCl_3_): *δ* 10.48 (br.s, 1H, COOH), 4.71 (dd, 1H, *J*_H-F(2)_ = 14.2 Hz, *J*_H-F(2)_ = 6.9 Hz, CH). ^19^F NMR (CDCl_3_): *δ* −80.9 (m, 3F, CF_3_), −104.7 (dm, 1F, *J*_A2,B2_ = 247 Hz, F_A_-2), −115.4 (dm, 1F, *J*_A2,B2_ = 247 Hz, F_B_-2), −117.9 (dm, 1F, *J*_A,B_ = 298 Hz, F_A_-CF_2_CF_3_), −120.6 (dm, 1F, *J*_A,B_ = 298 Hz, F_B_-CF_2_CF_3_), −133.6 (dm, 1F, *J* = 47 Hz, F-4), −137.6 (ddd, 1F, *J*_7,6_ = 20.8 Hz, *J*_7,4_ = 15.9 Hz, *J*_7,5_ = 5.3 Hz, F-7), −145.6 (dddd, 1F, *J*_6,7_ = 20.8 Hz, *J*_6,5_ = 19.8 Hz, *J*_6,4_ = 8.4 Hz, *J* = 5 Hz, F-6), −149.9 (dddm, 1F, *J*_5,4_ = 20.0 Hz, *J*_5,6_ = 19.8 Hz, *J*_5,7_ = 5.3 Hz, F-5). Anal. Calcd for C_12_H_2_F_12_O_2_: C, 35.49; H, 0.50; F, 56.14%. Found: C, 35.30; H, 0.72; F, 56.01%. HRMS *m/z*, 405.9852 (M^+^). Calcd. M = 405.9858.

### 3.14. Carbonylation of 2,2,4,5,6,7-Hexafluoro-3,3-bis(pentafluoroethyl)indan-1-ol *(**1l**)*

*a.* Alcohol **1l** (0.384 g, 0.80 mmol), FSO_3_H (0.162 g, 1.62 mmol), and SbF_5_ (0.351 g, 1.62 mmol) (molar ratio 1:2:2), according to the typical procedure (Section 3.1) (3 h, r.t.), after evaporation of the solvent and sublimation (120 °C, 1 Torr), gave 0.290 g of acid **2l** (yield 71%).

*b.* Alcohol **1l** (0.258 g, 0.54 mmol), FSO_3_H (0.177 g, 1.77 mmol), and SbF_5_ (0.386 g, 1.78 mmol) (molar ratio 1:3:3), according to the typical procedure (Section 3.1) (6.5 h, 70 °C), gave a mixture of compounds containing 71% of **2l** and 27% of **10l**.

*2,2,4,5,6,7-Hexafluoro-3,3-bis(pentafluoroethyl)indan-1-carboxylic acid (***2l***).* White solid, mp 113.6–114.8 °C. IR (film) ν, cm^−1^: 2926 (CH), 1743 (C=O), 1512 (FAR). ^1^H NMR (CDCl_3_): *δ* 7.5 (br.s, 1H, COOH), 4.72 (t, 1H, *J*_H,F(2)_ = 13.8 Hz, CH). ^19^F NMR (CDCl_3_): *δ* −76.8 (dm, 3F, *J* = 35 Hz, CF_3_), −78.4 (dtm, 3F, *J* = 31.3 Hz, *J* = 14.4 Hz, CF_3_), −96.1 (dm, 1F, *J*_A2,B2_ = 258 Hz, F_A_-2), −99.3 (dm, 1F, *J*_A2,B2_ = 258 Hz, F_B_-2), −102.8 (dm, 1F, *J*_A,B_ = 293 Hz, F_A_-CF_2_CF_3_), −105.9 (dm, 1F, *J*_A,B_ = 293 Hz, F_B_-CF_2_CF_3_), −106.1 (dm, 1F, *J*_A,B_ = 297 Hz, F_A_-CF_2_CF_3_), −109.8 (dm, 1F, *J*_A,B_ = 297 Hz, F_B_-CF_2_CF_3_), −130.9 (m, 1F, F-4), −136.5 (dddm, 1F, *J*_7,6_ = 21.2 Hz, *J*_7,4_ = 14.9 Hz, *J*_7,5_ = 5 Hz, F-7), −147.1 (dddm, 1F, *J*_6,7_ = 21.2 Hz, *J*_6,5_ = 19.9 Hz, *J*_6,4_ = 8.8 Hz, F-6), −150.4 (dddd, 1F, *J*_5,4_ = 19.9 Hz, *J*_5,6_ = 19.9 Hz, *J*_5,7_ = 5 Hz, *J*_F(5),H_ = 2.3 Hz F-5). Anal. Calcd for C_14_H_2_F_16_O_2_: C, 33.22; H, 0.4. Found: C, 33.24; H, 0.53. HRMS *m/z*, 505.9796 (M^+^). Calcd. M = 505.9794.

### 3.15. Carbonylation of 2,2,4,5,6,7-Hexafluoroindan-1-ol *(**1m**)*

*a.* Alcohol **1m** (0.324 g, 1.34 mmol), FSO_3_H (0.284 g, 2.84 mmol), and SbF_5_ (0.618 g, 2.85 mmol) (molar ratio 1:2:2), according to the typical procedure (Section 3.1) (1.5 h, r.t.), gave a dark mixture of compounds containing ~70% of acid **2m** along with resinification products.

*b.* Alcohol **1m** (0.298 g, 1.23 mmol), TfOH (0.864 g, 5.76 mmol), and SbF_5_ (0.260 g, 1.20 mmol) (molar ratio 1:5:1), according to the typical procedure (Section 3.1) (1.5 h, 50 °C), after evaporation of the solvent and sublimation (130 °C, 1 Torr), afforded 0.317 g of acid **2m** (yield 95%).

*2,2,4,5,6,7-Hexafluoroindan-1-carboxylic acid (***2m***).* White solid, mp 79.5–80.8 °C (CCl_4_). IR (KBr) ν, cm^−1^: 2939 (CH), 1734 (C=O), 1512, 1502 (FAR). ^1^H NMR (CDCl_3_): *δ* 7.00 (br.s, 1H, COOH), 4.51 (ddm, 1H, *J* = 16.4 Hz, *J* = 3.3 Hz, H-1), 3.78–3.41 (m, 2H, H-3). ^19^F NMR (CDCl_3_): *δ* −90.1 (dm, 1F, *J*_A2,B2_ = 237 Hz, F_A_-2), −101.6 (dm, 1F, *J*_A2,B2_ = 237 Hz, F_B_-2), −140.7 (dddm, 1F, *J*_7,6_ = 20.5 Hz, *J*_7,4_ = 17 Hz, *J*_F(7),H(1)_ = 3.3 Hz, F-7), −141.8 (ddm, 1F, *J*_4,5_ = 20 Hz, *J*_4,7_ = 17 Hz, F-4), −154.1 (ddm, 1F, *J*_6,7_ = 20.5 Hz, *J*_6,5_ = 19.7 Hz, F-6), −155.8 (ddm, 1F, *J*_5,4_ = 20 Hz, *J*_5,6_ = 19.7 Hz, F-5). Anal. Calcd for C_10_H_4_F_6_O_2_: C, 44.46; H, 1.49; F, 42.20%. Found: C, 44.13; H, 1.59; F, 42.36%. HRMS *m/z*, 270.0112 (M^+^). Calcd. M = 270.0110.

### 3.16. Carbonylation of 2,2,4,5,6,7-Hexafluoro-2,3-dihydrobenzofuran-3-ol *(**1n**)*

*a.* Alcohol **1n** (0.410 g, 1.68 mmol), FSO_3_H (0.336 g, 3.36 mmol), and SbF_5_ (0.729 g, 3.37 mmol) (molar ratio 1:2:2), according to the typical procedure (Section 3.1) (2 h, r.t.), gave a mixture of compounds containing ~85% of acid **10n**. Evaporation of the solvent and sublimation (125 °C, 1 Torr) resulted in 0.373 g of a mixture of acid **10n** and another product (apparently, 4,5,6,7-tetrafluoro benzofuran-2(*3H*)-one; GC-MS: *M* 206) in the 92:8 ratio. This solid was dissolved in a mixture of CH_2_Cl_2_ (10 mL) and a saturated aqueous solution of NaHCO_3_ (15 mL). The aqueous layer was separated, acidified with HCl (pH < 1), and extracted with Et_2_O (2 × 5 mL). Evaporation of the solvent gave 0.300 g of acid **10n** (yield 71%).

*b.* A mixture of alcohol **1n** (0.200 g, 0.82 mmol), FSO_3_H (0.164 g, 1.64 mmol), and SbF_5_ (0.355 g, 1.64 mmol) (molar ratio 1:2:2) was vigorously stirred (2 h, r.t.) in a round-bottom glass flask (10 mL) in a slow flow of CO at atmospheric pressure. The reaction mixture was diluted with FSO_3_H (0.295 g, 2.95 mmol) and SbF_5_ (0.640 g, 2.96 mmol) and then placed into an NMR tube and analyzed by ^19^F NMR spectroscopy. The signal of FSO_3_H served as an internal standard (41.1 ppm). The spectrum contained signals of a cationic species with a benzofuran moiety: *δ* −65.1 (d, 1F, *J* = 8 Hz, F-2), −143.0 (m, 1F, F-4), −154.8 (td, 1F, *J* = 18 Hz, *J* = 8 Hz), −156.5 (t, 1F, *J* = 19 Hz), −157.6 (t, 1F, *J* = 16 Hz). The mixture was poured into 5% hydrochloric acid (10 mL), extracted with Et_2_O (3 × 4 mL), and dried over MgSO_4_. The resulting solution contained 0.193 g of acid **10n**.

*c.* A mixture of alcohol **1n** (0.31 g, 1.27 mmol), FSO_3_H (0.253 g, 2.53 mmol), and SbF_5_ (0.551 g, 2.54 mmol) (molar ratio 1:2:2) was intensively stirred (2 h, r.t.) in a round-bottom glass flask (10 mL) in a slow flow of CO under atmospheric pressure. After that, acid **10j** (0.046 g, 0.17 mmol) was added to the reaction mixture followed by incubation at r.t. for 20 h. The mixture was poured into 5% hydrochloric acid (10 mL), extracted with a CH_2_Cl_2_–Et_2_O mixture (3:1) (3 × 4 mL), and dried over MgSO_4_. The resultant solution contained acids **10n** and **10j** in the 87:13 ratio and did not contain acid **2j**. A similar experiment with heating of the reaction mixture (4.5 h, 70 °C) after addition of acid **10j** yielded the same result.

*Perfluorobenzofuran-3-carboxylic acid (***10n***).* White solid, mp 141.5–142.5 °C (CHCl_3_ + Et_2_O). IR (KBr) ν, cm^−1^: 1705 (C=O), 1489, 1471 (FAR). ^1^H NMR (CO(CD_3_)_2_): *δ* 11.2 (br.s, 1H, COOH). ^19^F NMR (CO(CD_3_)_2_): *δ* −92.8 (ddt, 1F, *J*_2,6_ = 12.7 Hz, *J*_2,4_ = 4 Hz, *J*_2,5_ = *J*_2,7_ = 1.7 Hz, F-2), −140.5 (ddd, 1F, *J*_4,5_ = 19.9 Hz, *J*_4,7_ = 15.1 Hz, *J*_4,2_ = 4 Hz, F-4), −160.9 (ddd, 1F, *J*_6,7_ = 20 Hz, *J*_6,5_ = 19.9 Hz, *J*_6,2_ = 12.6 Hz, F-6), −161.3 (ddt, 1F, *J*_5,4_ = 19.9 Hz, *J*_5,6_ = 19.9 Hz, *J*_2,5_ = *J*_5,7_ = 1.7 Hz, F-5), −161.9 (ddt, 1F, *J*_7,6_ = 20 Hz, *J*_7,4_ = 15.1 Hz, *J*_2,7_ = *J*_5,7_ = 1.7 Hz, F-7). HRMS *m/z*, 251.9837 (M^+^). Calcd. for C_9_H_1_F_5_O_3_: M = 251.9840.

### 3.17. Carbonylation of 2,2,3,3,5,6,7,8-Octafluoro-4-hydroxytetralin-1-one *(**1o**)*

Alcohol **1o** (0.34 g, 1.11 mmol), FSO_3_H (0.394 g, 3.94 mmol), and SbF_5_ (0.856 g, 3.95 mmol) (molar ratio 1.0:3.5:3.5), according to the typical procedure (Section 3.1) (6 h, 70 °C), afforded a complex mixture of compounds containing ~30% of acid **11**, ~10% of perfluoronaphthalene-1,4-dione, and ~10% of naphthalen-1(4*H*)-one. 

### 3.18. Carbonylation of 2,2,4,5,6,7-Hexafluoroindan-1,3-diol *(**17a**)*

Diol **17a** (0.271 g, 1.05 mmol), FSO_3_H (0.422 g, 4.22 mmol), and SbF_5_ (0.919 g, 4.24 mmol) (molar ratio 1:4:4), according to the typical procedure (Section 3.1) (4 h, r.t.), gave a mixture of compounds containing ~95% of diacid **18a** (*cis:trans* = 45:50); judging by ^19^F NMR with an internal standard: the yield was 91%. The solvent was evaporated, and the residue was washed with CH_2_Cl_2_ (3 mL), dissolved in Et_2_O (5 mL), and filtered; the obtained solution contained isomers of diacid **18a** (*cis:trans* = 50:50). Evaporation of the solvent and sublimation (170 °C, 1 Torr) afforded 0.200 g (yield 61%) of diacid **18a** (*cis:trans* = 38:62).

*2,2,4,5,6,7-Hexafluoroindan-1,3-dicarboxylic acid (***18a***),* (*cis:trans* = 38:62). White solid, mp 215 °C (with decomposition). IR (KBr) ν, cm^−1^: 1738 (C=O), 1510 (FAR). *Cis*-**18a**. ^1^H NMR (CO(CD_3_)_2_): *δ* 7.3 (br.s, COOH), 4.94 (dd, 2H, *J*_H,F(A2)_ = 15.2 Hz, *J*_H,F(B2)_ = 13.1 Hz, CH). ^19^F NMR (CO(CD_3_)_2_): *δ* −88.4 (dt, 1F, *J*_A2,B2_ = 238 Hz, *J*_F(A2),H_ = 15.2 Hz, F_A_-2), −100.7 (dt, 1F, *J*_A2,B2_ = 238 Hz, *J*_F(B2),H_ = 13.1 Hz, F_B_-2), −139.2 (m, 2F, F-4,7), −155.3 (m, 2F, F-5,6). *Trans*-**18a**. ^1^H NMR (CO(CD_3_)_2_): *δ* 7.3 (br.s, COOH), 4.92 (t, 2H, ^3^*J*_H,F(2)_ = 12.5 Hz, CH). ^19^F NMR (CO(CD_3_)_2_): *δ* −96.5 (t, 2F, ^3^*J*_F(2),H_ = 12.5 Hz, F-2), −139.5 (m, 2F, F-4,7), −154.8 (m, 2F, F-5,6). Anal. Calcd for C_11_H_4_F_6_O_4_: C, 42.06; H, 1.28; F, 36.29%. Found: C, 42.70; H, 1.53; F, 36.13%. HRMS *m/z*, 314.0009 (M^+^). Calcd. M = 314.0008.

### 3.19. Carbonylation of 2,2,3,3,5,6,7,8-Octafluorotetralin-1,4-diol *(**17b**)*

*a.* Diol **17b** (0.203 g, 0.66 mmol), FSO_3_H (0.315 g, 3.15 mmol), and SbF_5_ (0.686 g, 3.17 mmol) (molar ratio 1:5:5), according to the typical procedure (Section 3.1) (23 h, r.t.), gave a mixture of compounds **18b** (isomer A), **18b** (isomer B), and **19** in the 32:28:40 ratio. Evaporation of the solvent and sublimation (180 °C, 1 Torr) resulted in a mixture (0.170 g) of compounds **18b** (isomer A), **18b** (isomer B), and **19** at the 28:32:40 ratio. The mixture was heated (52 h, 75–80 °C) with MeOH (5 mL) and 96% H_2_SO_4_ (1 mL) in a sealed tube and then diluted with water (20 mL) and extracted with CH_2_Cl_2_ (3 × 4 mL). The extract was washed with a saturated aqueous solution of NaHCO_3_ (15 mL). The organic layer was dried over MgSO_4_ and evaporated to obtain 89 mg of a mixture of compounds containing diesters **18bMe** and **19Me** in the 95:5 ratio. Silica gel column chromatography (CHCl_3_ as eluents) gave 61 mg of diesters **18bMe** (isomer A), **18bMe** (isomer B), and **19Me** in the 20:25:55 ratio. 

The resultant mixture of diesters **18bMe** and **19Me** (50 mg) was dissolved in dry CHCl_3_ (0.5 mL), after which NEt_3_ (40 mg) was introduced. The mixture was kept at r.t. for 20 h and washed with 5% hydrochloric acid (2 × 2 mL). Evaporation of the solvent and sublimation (110 °C, 1 Torr) afforded 42 mg of diester **19Me** (yield 88%).

*b.* Diol **17b** (0.739 g, 2.40 mmol), FSO_3_H (1.149 g, 11.49 mmol), and SbF_5_ (2.500 g, 11.54 mmol) (molar ratio 1:5:5), according to the typical procedure (Section 3.1) (5 h, 70 °C), gave a mixture of compounds **19**, **20**, and **21** at the 80:8:12 ratio. Evaporation of the solvent and sublimation (150 °C, 1 Torr) resulted in 0.275 g of compounds **19**, **20**, and **21** in the 40:25:35 ratio; subsequent sublimation (220 °C, 1 Torr) gave 0.390 g (yield 50%) of diacid **19**. The first sublimation fraction was heated (14 h, 70 °C) with SOCl_2_ (0.5 mL) and DMF (one drop). SOCl_2_ was evaporated, and the obtained mixture was heated (23 h, 70 °C) with EtOH (3 mL). Silica gel column chromatography (CCl_4_–CHCl_3_ [1:1] as the eluent) gave 0.153 g of a mixture of esters **20Et** and **21Et** in the 40:60 ratio.

*c.* Diol **17b** (0.413 g, 1.34 mmol), FSO_3_H (0.294 g, 2.94 mmol), SbF_5_ (0.640 g, 2.95 mmol) (molar ratio 1.0:2.2:2.2), and C_6_F_6_ (0.5 mL), according to the typical procedure (Section 3.1) (3 h, r.t.), gave a mixture of compounds **18b**, **19**, **22**, and **23** in the 7:7:75:11 ratio. The extract was washed with a saturated aqueous solution of NaHCO_3_ (15 mL), and the organic layer was dried over MgSO_4_. Evaporation of the solvent and sublimation (95 °C, 1 Torr) afforded 0.253 g (yield 59%) of lactone **22** (*cis:trans* = 38:62).

A mixture of lactone **22** (0.140 g), 5% sulfuric acid (5 mL), and Et_2_O (5 mL) was kept at r.t. for 12 weeks. The organic layer was separated and dried over MgSO_4_; evaporation of the solvent gave 0.147 g (yield 99%) of acid **23** as one isomer.

*2,2,3,3,5,6,7,8-Octafluorotetralin-1,4-dicarboxylic acid (***18b***)* in the mixture with acid **19** [**18b**(isomer A):**18b**(isomer B):**19** = 28:32:40]. *Isomer A:* ^1^H NMR (CO(CD_3_)_2_): *δ* 10.12 (br.s, COOH), 4.96 (tm, 2H, *J*_H,F_ = 13 Hz, CH). ^19^F NMR (CO(CD_3_)_2_): *δ* −114.0 (dm, 2F, *J*_A,B_ ~ 260 Hz, F_A_-CF_2_), −120.6 (dm, 2F, *J*_A,B_ ~ 260 Hz, F_B_-CF_2_), −138.3 (m, 2F, F-5,8), −156.1 (m, 2F, F-6,7). *Isomer B:* ^1^H NMR (CO(CD_3_)_2_): *δ* 10.12 (br.s, COOH), 4.96 (tm, 2H, *J*_H,F_ = 13 Hz, CH). ^19^F NMR (CO(CD_3_)_2_): *δ* −115.1 (dm, 2F, *J*_A,B_ = 259 Hz, F_A_-CF_2_), −122.7 (dm, 2F, *J*_A,B_ = 259 Hz, F_B_-CF_2_), −137.2 (m, 2F, F-5,8), −155.8 (m, 2F, F-6,7).

*Dimethyl 1,2,2,3,3,5,6,7,8-nonafluorotetralin-1,4-dicarboxylate (***18bMe***)* in the mixture with diester **19Me** [**18bMe**(isomer A):**18bMe**(isomer B):**19Me** = 20:25:55]. *Isomer A*: ^1^H NMR (CDCl_3_): *δ* 4.53 (m, 2H, CH), 3.82 (s, 6H, CH_3_). ^19^F NMR (CDCl_3_): *δ* −116.0 (dm, 2F, *J*_A,B_ ~ 262 Hz, F_A_-CF_2_), −122.7 (dm, 2F, *J*_A,B_ ~ 262 Hz, F_B_-CF_2_), −139.6 (m, 2F, F-5,8), −154.5 (m, 2F, F-6,7). *Isomer B*: ^1^H NMR (CDCl_3_): *δ* 4.53 (m, 2H, CH), 3.83 (s, 6H, CH_3_). ^19^F NMR (CDCl_3_): *δ* −117.6 (dm, 2F, *J*_A,B_ = 261 Hz, F_A_-CF_2_), −125.0 (dm, 2F, *J*_A,B_ = 261 Hz, F_B_-CF_2_), −138.4 (m, 2F, F-5,8), −154.3 (m, 2F, F-6,7). HRMS *m/z*, 392.0286 (M^+^). Calcd. for C_14_H_8_F_8_O_4_: M = 392.0289.

*Perfluoronaphthalene-1,4-dicarboxylic acid (***19***).* White solid, mp 265–268 °C (with decomposition). IR (KBr) ν, cm^−1^: 1720 (C=O), 1535, 1508, 1479, 1454 (FAR). ^1^H NMR (CO(CD_3_)_2_): *δ* 6.3 (br.s, 2H, COOH). ^19^F NMR (CO(CD_3_)_2_): *δ* −135.2 (m, 2F, F-2,3), −141.4 (m, 2F, F-5,8), −155.0 (m, 2F, F-6,7). HRMS *m/z*, 323.9854 (M^+^). Calcd. for C_12_H_2_F_6_O_4_: M = 323.9852.

*Dimethyl perfluoronaphthalene-1,4-dicarboxylate (***19Me***)*. White solid, mp 112.5–114 °C (hexane). IR (KBr) ν, cm^−1^: 2970 (CH), 1745 (C=O), 1531, 1512 (FAR). ^1^H NMR (CDCl_3_): *δ* 4.03 (s, 6H, CH_3_). ^19^F NMR (CDCl_3_): *δ* −134.4 (s, 2F, F-2,3), −142.9 (m, 2F, F-5,8), −154.5 (m, 2F, F-6,7). Anal. Calcd for C_14_H_6_F_6_O_4_: C, 47.75; H, 1.72; F, 32.37%. Found: C, 48.11; H, 2.13; F, 31.87%. HRMS *m/z*, 352.0168 (M^+^). Calcd. M = 352.0165.

*Ethyl 2,3,4,5,6,7,8-heptafluoro-1-naphthoate (***20Et***)* in the mixture with ester **21Et** (**20Et**:**21Et** = 40:60). ^1^H NMR (CDCl_3_): *δ* 4.51 (q, 2H, *J*_CH2,CH3_ = 7, CH_2_), 1.40 (t, 3H, *J*_CH2,CH3_ = 7, CH_3_). ^19^F NMR (CDCl_3_): *δ* −132.4 (m, 1F, F-2), −137.3 (dm, 1F, *J*_4,5_ = 60.3 Hz, F-4), −142.3 (m, 1F, F-8), −145.7 (dm, 1F, *J*_5,4_ = 60.3 Hz, F-5), −153.9 (m, 1F, F-7), −155.4 (m, 1F, F-6), −156.9 (m, 1F, F-3). HRMS, *m/z*: 326.0174 (M^+^). Calcd. for C_13_H_5_F_7_O_2_: M = 326.0172.

*Ethyl 2,3,5,6,7,8-hexafluoro-1-naphthoate (***21Et***)* in the mixture with ester **21Et** (**20Et**:**21Et** = 40:60). ^1^H NMR (CDCl_3_): *δ* 7.84 (ddd, 1H, *J*_H,F_ = 9.5 Hz, *J*_H,F_ = 7.7 Hz, *J*_H,F_ = 1 Hz, H-4), 4.51 (q, 2H, *J*_CH2,CH3_ = 7 Hz, CH_2_), 1.42 (t, 3H, *J*_CH2,CH3_ = 7, CH_3_). ^19^F NMR (CDCl_3_): *δ* −132.4 (m, 1F, F-3), −135.6 (m, 1F, F-2), −143.9 (m, 1F, F-8), −148.0 (m, 1F, F-5), −156.3 (m, 1F, F-7), −156.9 (m, 1F, F-7). HRMS, *m/z*: 308.0269 (M^+^). Calcd. for C_13_H_6_F_6_O_2_: M = 308.0267.

*2,2,3,3,5,6,7,8-Octafluoro-1,4-(epoxymethano)tetralin-9-one (**22**).* White solid, mp 100.6–101.6 °C. IR (KBr) ν, cm^−1^: 2926 (CH), 1809 (C=O), 1512 (FAR). ^1^H NMR (CO(CD_3_)_2_): *δ* 6.74 (m, 1H, H-1), 5.42 (m, 1H, H-4). ^19^F NMR (CO(CD_3_)_2_): *δ* −111.2 (dm, 1F, *J*_A,B_ = 241 Hz, F_A_-CF_2_), −112.2 (dm, 1F, *J*_A,B_ = 241 Hz, F_B_-CF_2_), −115.8 (dm, 1F, *J*_A,B_ = 247 Hz, F_A_-CF_2_), −122.8 (dm, 1F, *J*_A,B_ = 247 Hz, F_B_-CF_2_), −141.1 (ddd, 1F, *J*_8,7_ = 20.5 Hz, *J*_8,5_ = 16 Hz, *J*_8.6_ = 6 Hz, F-8), −141.6 (ddd, 1F, *J*_5,6_ = 20 Hz, *J*_8,5_ = 16 Hz, *J*_5,7_ = 5 Hz, F-5), −148.8 (ddd, 1F, *J*_6,5_ = 20 Hz, *J*_6,7_ = 18.5 Hz, *J*_6,8_ = 6 Hz, F-6), −151.1 (ddd, 1F, *J*_7,8_ = 20.5 Hz, *J*_7,6_ = 18.5 Hz, *J*_7,5_ = 5 Hz, F-7). Anal. Calcd for C_11_F_8_H_2_O_2_: C, 41.53; H, 0.63; F, 47.78%. Found: C, 41.95; H, 0.69; F, 48.16%. HRMS *m/z*, 317.9921 (M^+^). Calcd. M = 317.9922.

*2,2,3,3,5,6,7,8-Octafluoro-4-hydroxytetralin-1-carboxylic acid (***23***).* White solid, mp 150.1–151.8 °C. IR (KBr) ν, cm^−1^: 2982 (CH), 1755 (C=O), 1524, 1489 (FAR). ^1^H NMR (CO(CD_3_)_2_): *δ* 5.95 (br.s, 2H, COOH and OH), 5.43 (td, 1H, *J*_H,F_ = 8.5 Hz, *J*_H,F_ = 4.5 Hz, H-4), 4.86 (ddm, 1H, *J*_H,F_ = 16 Hz, *J*_H,F_ = 15 Hz, H-1). ^19^F NMR (CO(CD_3_)_2_): *δ* −114.8 (dm, 1F, *J*_A,B_ = 263 Hz, F_A_-CF_2_), −116.0 (dm, 1F, *J*_A,B_ = 263 Hz, F_B_-CF_2_), −122.2 (dm, 1F, *J*_A,B_ = 264 Hz, F_A_-CF_2_), −133.6 (dm, 1F, *J*_A,B_ = 264 Hz, F_B_-CF_2_), −136.7 (dddm, 1F, *J*_8,7_ = 19.8 Hz, *J*_8,5_ = 12 Hz, *J*_8,6_ = 3.7, *J* = 12 Hz, F-8), −141.6 (dddm, 1F, *J*_5,6_ = 20 Hz, *J*_5,8_ = 12 Hz, *J*_6,7_ = 3.7, F-5), −155.3 (ddd, 1F, *J*_7,6_ = 20 Hz, *J*_8,7_ = 19.8 Hz, *J*_7,5_ = 3.7 Hz, F-7), −155.5 (dddm, 1F, *J*_6,5_ = 20 Hz, *J*_6,7_ = 20 Hz, *J*_6,8_ = 3.9 Hz, F-6). HRMS, *m/z*: 336.0023 (M^+^). Calcd. for C_11_H_4_F_8_O_3_: M = 336.0027.

### 3.20. Carbonylation of (2,3,4,5-Tetrafluoro-6-(hydroxymethyl)phenyl)methanol *(**17c**)*

*a.* Diol **17c** (0.200 g, 0.95 mmol) and TfOH (1.000 g, 6.67 mmol) (molar ratio 1:7), according to the typical procedure (Section 3.1) (4 h, 70 °C), gave a mixture of compounds containing ~25% of lactone **24c**.

*b.* Diol **17c** (0.200 g, 0.95 mmol), TfOH (1.035 g, 6.90 mmol), and SbF_5_ (0.247 g, 1.14 mmol) (molar ratio 1.0:7.2:1.2), according to the typical procedure (Section 3.1) (2 h, r.t.), afforded a mixture of compounds containing ~80% of lactone **24c**. Silica gel column chromatography (CH_2_Cl_2_ as eluents) and sublimation (95 °C, 1 Torr) gave 0.115 g (yield 55%) of lactone **24c**.

*c.* Diol **17c** (0.300 g, 1.43 mmol), FSO_3_H (0.570 g, 5.70 mmol), and SbF_5_ (1.240 g, 5.72 mmol) (molar ratio 1:4:4), according to the typical procedure (Section 3.1) (4 h, r.t.), gave a dark mixture containing ~25% of lactone **24c** along with other compounds and resinification products.

*5,6,7,8-Tetrafluoroisochroman-3-one (***24c***)*. White solid, mp 101.1–102.2 °C. IR (KBr) ν, cm^−1^: 2974 (CH), 1747, 1730 (C=O), 1516, 1498 (FAR). ^1^H NMR (CDCl_3_): *δ* 5.39 (s, 2H, H-1), 3.73 (s, 2H, H-4). ^19^F NMR (CDCl_3_): *δ* −145.4 (m, 2F), −155.1 (m, 1F), −157.0 (m, 1F). Anal. Calcd for C_9_F_4_H_4_O_2_: C, 49.11; H, 1.83; F, 34.52%. Found: C, 49.01; H, 2.03; F, 34.58%. HRMS m/z, 220.0145 (M^+^). Calcd. M = 220.0142.

### 3.21. Carbonylation of (Pentafluorophenyl)(2,3,4,5-tetrafluoro-6-(hydroxymethyl)phenyl)methanol *(**17d**)*

Diol **17d** (0.200 g, 0.53 mmol), FSO_3_H (0.212 g, 2.12 mmol), and SbF_5_ (0.462 g, 2.13 mmol) (molar ratio 1:4:4), according to the typical procedure (Section 3.1) (5 h, r.t.), gave a mixture of compounds containing 50% of **24d** and 45% of **25d**. Silica gel column chromatography (CHCl_3_ as eluents) resulted in 0.150 g (yield 73%) of a mixture of lactones **24d** and **25d** in the 56:44 ratio.

5,6,7,8-Tetrafluoro-1-(pentafluorophenyl)isochroman-3-one (**24d**) and 5,6,7,8-tetrafluoro-4-(pentafluorophenyl)isochroman-3-one (**25d**) (**24d**:**25d** = 56:44). White solid. IR (KBr) ν, cm^−1^: 2926, 2901 (CH), 1753 (C=O), 1512, 1498 (FAR). Compound **24d**: ^1^H NMR (CDCl_3_): δ 7.00 (s, 1H, H-1), 3.92 (dm, 1H, *J*_A4,B4_ = 21.7 Hz, H_A_-4), 3.84 (dm, 1H, *J*_A4,B4_ = 21.7 Hz, H_B_-4). ^19^F NMR ((CDCl_3_): δ −143.3 (m, 1F, F-8), −143.8 (dddt, 1F, *J*_5,6_ = 20.8 Hz, *J*_5,8_ = 13 Hz, *J*_5,7_ = 3.1 Hz, *J*_5,H_ = 1.5 Hz, F-5), −144.4 (m, 2F, F-ortho), −150.8 (tt, 1F, *J*_para,meta_ = 21 Hz, *J*_para,ortho_ = 3.3 Hz, F-para), −153.9 (ddd, 1F, *J*_6,5_ = 20.8 Hz, *J*_6,7_ = 20.6 Hz, *J*_6,8_ = 4 Hz, F-6), −156.5 (dddt, 1F, *J*_7,6_ = 20.6 Hz, *J*_7,8_ = 20.6 Hz, *J*_7,5_ = 3.1 Hz, *J*_F(7),H_ = 2 Hz, F-7), −160.8 (m, 2F, F-meta). Compound (**25d**): ^1^H NMR (CDCl_3_): δ 5.64 (dm, 1H, J_A1,B1_ ~ 17 Hz, H_A_-1), 5.61 (dm, 1H, J_A1,B1_ ~ 17 Hz, H_B_-1), 5.36 (s, 1H, H-4). ^19^F NMR (CDCl_3_): δ −143.2 (m, 1F, F-5), −143.3 (m, 2F, F-ortho), −144.4 (m, 1F, F-8), −153.2 (tt, 1F, *J*_para,meta_ = 20.5 Hz, *J*_para,ortho_ = 3.8 Hz, F-para), −154.5 (m, 1F, F-7 or F-6), −154.8 (m, 1F, F-6 or F-7), −161.4 (m, 2F, F-meta). Anal. Calcd for C_15_H_3_F_9_O_2_: C, 46.65; H, 0.78; F, 44.28%. Found: C, 46.58; H, 0.93; F, 44.32%. HRMS *m/z*, 385.9986 (M^+^). Calcd. M = 385.9984.

### 3.22. Carbonylation of 2,2,2-Trifluoro-1-(2,3,4,5-tetrafluoro-6-(hydroxymethyl)phenyl)ethan-1-ol *(**17e**)*

Diol **17e** (0.200 g, 0.72 mmol), FSO_3_H (0.295 g, 2.95 mmol), and SbF_5_ (0.641 g, 2.96 mmol) (molar ratio 1:4:4), according to the typical procedure (Section 3.1) (7 h, 50 °C), gave a mixture of compounds containing ~65% of lactone **24e**. Silica gel column chromatography (CHCl_3_ as the eluent) and recrystallization from hexane gave 66 mg of lactone **24e** (yield 32%).

*5,6,7,8-Tetrafluoro-1-(trifluoromethyl)isochroman-3-one (***24e***)*. White solid, mp 27.0–29.4 °C. IR (KBr) ν, cm^−1^: 2985 (CH), 1784 (C=O), 1525, 1506 (FAR). ^1^H NMR (CDCl_3_): *δ* 5.93 (q, 1H, *J*_H,CF3_ = 6.5 Hz, H-1), 3.99 (d, 1H, *J*_A4,B4_ = 20.9 Hz, H_A_-4), 3.68 (d, 1H, *J*_A4,B4_ = 20.9 Hz, H_B_-4). ^19^F NMR (CDCl_3_): *δ* −78.3 (dd, 3F, *J*_CF3,F(8)_ = 7.5 Hz, *J*_CF3,H(1)_ = 6.5 Hz, CF_3_), −141.8 (ddqd, 1F, *J*_8,7_ = 20.7 Hz, *J*_8,5_ = 14.1 Hz, *J*_F(8),CF3_ = 7.5 Hz, *J*_8,6_ = 5.4 Hz, F-8), −143.4 (ddd, 1F, *J*_5,6_ = 20.7 Hz, *J*_5,8_ = 14.1 Hz, *J*_5,7_ = 3.3 Hz, F-5), −151.0 (ddd, 1F, *J*_6,5_ = 20.7 Hz, *J*_6,7_ = 20.7 Hz, *J*_6,8_ = 5.4 Hz, F-6), −155.3 (dddd, 1F, *J*_7,6_ = 20.7 Hz, *J*_7,8_ = 20.7 Hz, *J*_7,5_ = 3.3 Hz, *J*_F(7),H_ = 2.5 Hz, F-7). Anal. Calcd for C_10_H_3_F_7_O_2_: C, 41.69; H, 1.05; F, 46.16%. Found: C, 41.72; H, 1.05; F, 45.79%. HRMS m/z, 288.0015 (M^+^). Calcd. M = 288.0016.

### 3.23. Carbonylation of (2,3,4,6-Tetrafluoro-5-(hydroxymethyl)phenyl) *(**17f**)*

Diol **17f** (0.200 g, 0.95 mmol), TfOH (1.000 g, 6.67 mmol), and SbF_5_ (0.217 g, 1.00 mmol) (molar ratio 1:7:1), according to the typical procedure (Section 3.1) (5 h, r.t.), after evaporation of the solvent and sublimation (190 °C, 1 Torr), afforded 0.175 g of acid **18f** (yield 69%).

*[3-(Carboxymethyl)-2,4,5,6-tetrafluorophenyl]acetic acid (***18f***).* White solid, mp 179.1–181.0 °C. IR (KBr) ν, cm^−1^: 2991 (CH), 1709 (C=O), 1502 (FAR). ^1^H NMR (CO(CD_3_)_2_): *δ* 9.21 (br.s, 2H, COOH), 3.78 (br.s, 4H, 2CH_2_). ^19^F NMR (CO(CD_3_)_2_): *δ* −121.9 (d quintet, 1F, *J*_2,5_ = 11.2 Hz, *J*_F(2),CH2_ = 1.6 Hz, F-2), −137.7 (d, 2F, *J*_4,5_ = *J*_6,5_ = 21 Hz, F-4,6), −166.7 (td, 1F, *J*_5,4_ = *J*_5,6_ = 21 Hz, *J*_5,2_ = 11.2, Hz, F-5). Anal. Calcd for C_10_F_4_H_6_O_4_: C, 45.13; H, 2.27; F, 28.55%. Found: C, 45.05; H, 2.34; F, 28.59%. HRMS m/z, 266.0192 (M^+^). Calcd. M = 266.0197.

### 3.24. Carbonylation of (2,3,5,6-Tetrafluoro-4-(hydroxymethyl)phenyl) *(**17g**)*

A mixture of diol **17g** (0.300 g, 1.43 mmol), FSO_3_H (0.700 g, 7.00 mmol), and SbF_5_ (1.520 g, 7.02 mmol) (molar ratio 1:5:5) was intensively stirred (6.5 h, r.t.) in a round-bottom glass flask (10 mL) in a slow flow of CO at atmospheric pressure. The mixture was poured into 5% hydrochloric acid (10 mL), the crystals were filtered off, the aqueous phase was extracted with Et_2_O (3 × 5 mL), and the extract was combined with the filtered-off crystals. Evaporation of the solvent and sublimation (230 °C, 1 Torr) gave 0.210 g of acid **18g** (yield 55%).

*[4-(Carboxymethyl)-2,3,5,6-tetrafluorophenyl]acetic acid (***18g***)*. White solid, mp 310 °C (with decomposition). IR (KBr) ν, cm^−1^: 2970 (CH), 1714 (C=O), 1491 (FAR). ^1^H NMR (DMSO-d_6_): *δ* 12.97 (br.s, 2H, 2COOH), 3.78 (s, 4H, 2CH_2_). ^19^F NMR (DMSO-d_6_): *δ* −144.5 (s, 4F). Anal. Calcd for C_10_F_4_H_6_O_4_: C, 45.13; H, 2.27; F, 28.55%. Found: C, 45.10; H, 2.27; F, 28.17%. HRMS m/z, 266.0195 (M^+^). Calcd. M = 266.0197.

### 3.25. Synthesis of Ester ***2iMe*** and Its Reactions with NEt_3_ and K_2_CO_3_

*a.* A mixture of acid **2i** (1.258 g, 4.11 mmol) with MeOH (15 mL) and 96% H_2_SO_4_ (3 mL) was heated (11.5 h, 70 °C) in a sealed tube and then diluted with water (100 mL), extracted with CH_2_Cl_2_ (3 × 10 mL), and the extract was dried over MgSO_4_. Evaporation of the solvent and distillation in vacuum (120–130 °C, 6 Torr) gave 1.144 g of ester **2iMe** (yield 87%).

*b.* Ester **2iMe** (0.492 g, 1.55 mmol) was dissolved in a mixture of dry CHCl_3_ (10 mL) and NEt_3_ (1 mL). The solution was incubated at r.t. for 15 min, washed with 5% hydrochloric acid (30 mL), and dried over MgSO_4_. Solvent evaporation yielded a mixture of compounds containing 65% of **10iMe** and 15% of **2iMe** (chromatographically inseparable). 

*c.* Ester **2iMe** (72 mg, 0.225 mmol) was dissolved in a mixture of dry CHCl_3_ (1.5 mL) and NEt_3_ (0.15 mL). The solution was kept at r.t. for 1 h, washed with 5% hydrochloric acid (10 mL), and dried over MgSO_4_. The mixture of compounds in the solution contained 65% of **10iMe** and 15% of **2iMe**.

*d.* Ester **2iMe** (0.292 g, 0.91 mmol) was dissolved in dry CHCl_3_ (5 mL) and stirred with K_2_CO_3_ (0.750 g) at r.t. for 26 h. The organic layer was filtered, and the mixture of compounds in it contained >90% of ester **10iMe** and was free of the starting compound. Silica gel column chromatography (CHCl_3_ as the eluent) gave 0.129 g (yield 47%) of ester **10iMe.**

*Methyl 2,2,3,3,4,5,6,7-octafluoroindan-1-carboxylate* (**2iMe**). Colorless liquid. IR (film) ν, cm^−1^: 2964 (CH), 1759 (C=O), 1520 (FAR). ^1^H NMR (CDCl_3_): *δ* 4.55 (dd, 1H, *J*_H,F(A2)_ = 14 Hz, *J*_H,F(B2)_ = 6.6 Hz, H-1), 3.86 (s, 3H, CH_3_). ^19^F NMR (CDCl_3_): *δ* −106.7 (dm, 1F, *J*_A3,B3_ = 259 Hz, F_A_-3), −109.7 (dm, 1F, *J*_A3,B3_ = 259 Hz, F_B_-3), −112.5 (dddd, 1F, *J*_A2,B2_ = 241 Hz, *J*_F(A2),H_ = 14 Hz, *J* = 3.6 Hz, *J* = 2.2 Hz, F_A_-2), −120.4 (dddd, 1F, *J*_A2,B2_ = 241 Hz, *J*_F(B2),H_ = 6.6 Hz, *J* = 4.5 Hz, *J* = 2.9 Hz, F_B_-2), −139.0 (ddd, 1F, *J*_7,6_ = 20.4 Hz, *J*_7,4_ = 16.8 Hz, *J*_7,5_ = 5.4 Hz, F-7), −140.8 (dddt, 1F, *J*_4,5_ = 20.4 Hz, *J*_4,7_ = 16.8 Hz, *J*_4.6_ = 7.2 Hz, *J* = 6.8 Hz, F-4), −146.3 (dddm, 1F, *J*_6,7_ = 20.4 Hz, *J*_6,5_ = 18.8 Hz, *J*_6,4_ = 7.2 Hz, F-6), −150.5 (dddm, 1F, *J*_5,4_ = 20.4 Hz, *J*_5,6_ = 18.8 Hz, *J*_4.6_ = 7.2 Hz, F-5). HRMS *m/z*, 320.0087 (M^+^). Calcd. for C_11_H_4_F_8_O_2_: M = 320.0078.

*Methyl perfluoro-1H-indene-3-carboxylate* (**10iMe**). Colorless liquid. IR (film) ν, cm^−1^: 2964 (CH), 1749 (C=O), 1516, 1497 (FAR). ^1^H NMR (CDCl_3_): *δ* 3.93 (s, 3H, CH_3_). ^19^F NMR (CDCl_3_): *δ* −120.6 (tdddd, 1F, *J*_2,1_ = 14.4 Hz, *J*_2,6_ = 14.4 Hz, *J*_2,4_ = 7.2 Hz, *J*_2,5_ = 2.6 Hz, *J*_2,7_ = 2.6 Hz, F-2), −125.2 (ddm, 2F, *J*_1,2_ = 14.4 Hz, *J*_1,7_ = 4.4 Hz, F-1), −136.4 (ddddt, 1F, *J*_4,5_ = 19.4 Hz, *J*_4,7_ = 14.8 Hz, *J*_4,2_ = 7.2 Hz, *J*_4,6_ = 4.4 Hz, *J*_4,1_ = 2 Hz, F-4), −140.0 (dddtd, 1F, *J*_76_ = 21.3 Hz, *J*_7,4_ = 14.4 Hz, *J*_7,5_ = 7.3 Hz, *J*_7,1_ = 4.3 Hz, *J*_7,2_ = 2.6 Hz, F-7), −146.9 (dddd, 1F, *J*_5,4_ = 19.4 Hz, *J*_5,6_ = 17.9 Hz, *J*_5,7_ = 7.3 Hz, *J*_5,2_ = 2.6 Hz, F-5), −153.7 (dddd, 1F, *J*_6,7_ = 21.1 Hz, *J*_6,5_ = 17.4 Hz, *J*_6,2_ = 14.5 Hz, *J*_6,4_ = 4.7 Hz, F-6). HRMS *m/z*, 300.0014 (M^+^). Calcd. for C_11_H_3_F_7_O_2_: M = 300.0016.

### 3.26. Synthesis of Ester ***2cMe*** and Its Reactions with NEt_3_ and K_2_CO_3_

*a.* A mixture of acid **2c** (1.170 g, 3.98 mmol) with MeOH (14 mL) and 96% H_2_SO_4_ (2.9 mL) was heated (16 h, 70 °C) in a sealed tube and then diluted with water (150 mL), extracted with CH_2_Cl_2_ (3 × 10 mL), and the extract was then washed with a saturated aqueous solution of NaHCO_3_ (20 mL), and finally dried over MgSO_4_. Evaporation of the solvent gave 1.100 g of ester **2cMe** (yield 90%).

*b.* Ester **2cMe** (55 mg, 0.18 mmol) was dissolved in a mixture of dry CHCl_3_ (0.5 mL) and NEt_3_ (36 mg, 0.36 mmol). The solution was kept at r.t. for 5 days, washed with 5% hydrochloric acid (1.5 mL), and dried over MgSO_4_. The mixture of compounds in the solution contained (as evidenced by GC-MS) 87% of starting ester **2cMe** and 6% of ester **2aMe** along with a number of unidentified compounds (M = 340−604). 

*c.* Ester **2cMe** (27 mg, 0.088 mmol) was dissolved in dry CHCl_3_ (0.6 mL) and stirred with K_2_CO_3_ (90 mg) at r.t. for 20 h. A mixture of compounds in the solution contained 97% of the starting compound and 2% of ester **10cMe**. The reaction mixture was heated (7 h, 55 °C), treated with 5% hydrochloric acid (5 mL), and dried over MgSO_4_. The mixture of compounds in the solution contained (judging by GC-MS) 12% of the starting ester **2cMe**, 13% of ester **10cMe**, and 72% of ester **2aMe** along with two unidentified compounds (M = 564).

*Methyl 3,3,3-trifluoro-2-(pentafluorophenyl)propanoate* (**2cMe**)

White solid, mp 42.5–43.5 °C. IR (film) ν, cm^−1^: 2962 (CH); 1770 (C=O); 1527, 1510 (FAR). ^1^H NMR (CDCl_3_): *δ* 4.71 (q, 1H, *J*_H,CF3_ = 8 Hz, CH), 3.84 (s, 3H, CH_3_). ^19^F NMR (CDCl_3_): *δ* −68.0 (dt, 3F, *J*_CF3,H_ = 8 Hz, *J*_CF3-F(ortho)_ = 8 Hz, CF_3_), −140.5 (m, 2F, F-*ortho*), −151.8 (tt, 1F, *J*_para,meta_ = 21 Hz, *J*_para,ortho_ = 3 Hz, F-*para*), −161.5 (m, 2F, F-*meta*). HRMS, *m/z*: 308.0064 (M^+^). Calcd. for C_10_H_4_F_8_O_2_: M = 308.0078.

*Methyl perfluoro-2-phenylacrylate (***10cMe***)* in the mixture with compounds **2cMe** and **2aMe**. ^19^F NMR (CDCl_3_): *δ* −62.8 (dt, 1F, *J*_3A,3B_ = 29.7 Hz, *J*_3A, *ortho*_ = 1.7 Hz, F-3A), −63.8 (dt, 1F, *J*_3A,3B_ = 29.7 Hz, *J*_3B,*ortho*_ = 6.2 Hz, F-3B), −139.1 (m, 2F, F-*ortho*), −152.8 (tt, 1F, *J_para,meta_* = 21 Hz, *J_para,orth_*_o_ = 2.5 Hz, F-*para*), −162.5 (m, 2F, F-*meta*). GC-MS, *m/z*: 288 (M^+^).

### 3.27. The Reaction of Acid ***2l*** with NEt_3_

Acid **2l** (185 mg, 0.37 mmol) was dissolved in a mixture of dry CHCl_3_ (3 mL) and NEt_3_ (0.186 g). The solution was kept at r.t. for 5 h, washed with 5% hydrochloric acid (2 × 10 mL), and dried over MgSO_4_. Evaporation of the solvent resulted in 0.170 g of acid **10l** (yield 95%).

*Perfluoro-1,1-diethyl-1H-indene-3-carboxylic acid (***10l***).* White solid, mp 120.2–121.3 °C (CHCl_3_). IR (KBr) ν, cm^−1^: 2929(CH), 1730(C=O), 1514, 1502(FAR). ^1^H NMR (CDCl_3_): *δ* 7.1 (br.s, 1H, COOH). ^19^F NMR (CDCl_3_): *δ* −81.8 (d, 6F, *J* = 3.6 Hz, CF_3_), −101.3 (m, 1F, F-2), −109.3 (dm, 4F, *J*_A,B_ ~ 285 Hz, F_A_-CF_2_), −112.0 (dm, 4F, *J*_A,B_ ~ 285 Hz, F_B_-CF_2_), −132.5 (tdddd, 1F, *J* = 49 Hz, *J*_7,6_ = 20.6 Hz, *J*_7,4_ = 14.6 Hz, *J*_7,5_ = 8.7 Hz, *J*_7,2_ = 2 Hz, F-7), −136.8 (dddd, 1F, *J*_4,5_ = 20.8 Hz, *J*_4,7_ = 14.6 Hz, *J*_4,2_ = 5.8 Hz, *J*_4,6_ = 4 Hz, F-4), −147.2 (dddd, 1F, *J*_5,4_ = 20.8 Hz, *J*_5,6_ = 18.9 Hz, *J*_5,7_ = 8.7 Hz, *J*_5,2_ = 1.7 Hz, F-5), −153.0 (dddd, 1F, *J*_6,7_ = 20.6 Hz, *J*_6,5_ = 18.9 Hz, *J*_6,2_ = 14.9 Hz, *J*_6,4_ = 4 Hz, F-6). Anal. Calcd for C_14_H_1_F_15_O_2_: C, 34.59%; H, 0.21%; F, 58.62%. Found: C, 35.12%; H, 0.43%; F, 58.48%. HRMS *m/z*, 485.9729 (M^+^). Calcd. M = 485.9732.

### 3.28. The Reaction of Acid ***2c*** with NEt_3_

*a.* Acid **2c** (50 mg, 0.17 mmol) was dissolved in a mixture of dry Et_2_O (0.5 mL) and NEt_3_ (55 mg). The solution was kept at r.t. for 14 days, washed with 5% hydrochloric acid (3 mL), and dried over MgSO_4_. The mixture of compounds in the extract contained 20% of starting acid **2c**, 75% of ethylbenzene **26**, and 3% of styrene **27**. 

*b.* Acid **2c** (30 mg, 0.10 mmol) was dissolved in a mixture of dry CDCl_3_ (0.5 mL) and NEt_3_ (50 mg). The solution was heated (27 h, 55 °C), treated with 5% hydrochloric acid (5 mL), and dried over MgSO_4_. The mixture of compounds in the extract contained 17% of starting acid **2c**, 34% of ethylbenzene **26**, and 35% of styrene **27**. 

### 3.29. Synthesis of Alcohol ***1j***

A mixture of alcohol **1i** (3.000 g, 10.79 mmol) with 20% oleum (10 mL) was heated (20 h, 100 °C) in a sealed tube and poured into ice water (100 mL). The resulting mixture was heated (5 h, 80 °C), extracted with Et_2_O (4 × 20 mL), and dried over MgSO_4_. Evaporation of the solvent and distillation in vacuum (150−160 °C, 1 Torr) gave 2.283 g of compound **1j** (yield 83%).

*2,2,4,5,6,7-Hexafluoro-3-hydroxyindan-1-one (***1j***).* Colorless liquid. IR (film) ν, cm^−1^: 2945 (CH), 1761 (C=O), 1506 (FAR). ^1^H NMR (CDCl_3_): *δ* 5.42 (d, 1H, *J*_H,F(A2)_ = 12.5 Hz, CH), 3.20 (s, 1H, OH). ^19^F NMR (CDCl_3_): *δ* −114.7 (dd, 1F, *J*_A2,B2_ = 285 Hz, *J*_F(A2),H_ = 12.5 Hz, F_A_-2), −126.8 (d, 1F, *J*_A2,B2_ = 285 Hz, F_B_-2), −135.0 (ddd, 1F, *J*_7,6_ = 19.8 Hz, *J*_7,4_ = 19 Hz, *J*_7,5_ = 11.7 Hz, F-7), −138.2 (ddd, 1F, *J*_5,4_ = 19.5 Hz, *J*_5,6_ = 19.5 Hz, *J*_5,7_ = 11.7 Hz, F-5), −140.7 (ddd, 1F, *J*_4,5_ = 19.5 Hz, *J*_4,7_ = 19 Hz, *J*_4,6_ = 5.4 Hz, F-4), −149.4 (ddd, 1F, *J*_6,7_ = 19.8 Hz, *J*_6,5_ = 19.5 Hz, *J*_6,4_ = 5.4 Hz, F-6). HRMS *m/z*, 255.9950 (M^+^). Calcd. for C_9_H_2_F_6_O_2_: M = 255.9954.

### 3.30. Synthesis of Alcohol ***1o***

A mixture of alcohol **1h** (3.000 g, 9.15 mmol) with 20% oleum (10 mL) was heated (21 h, 120 °C) in a sealed tube and poured into ice water (100 mL). The resultant mixture was heated (2.5 h, 80 °C), extracted with Et_2_O (4 × 20 mL), and the extract was dried over MgSO_4_. Evaporation of the solvent and distillation in vacuum (160−180 °C, 1 Torr) afforded 2.000 g of compound **1j** (yield 71%).

*2,2,3,3,5,6,7,8-Octafluoro-4-hydroxy-3,4-tetralin-1-one (***1o***).* Colorless liquid. IR (film) ν, cm^−1^: 2933 (CH), 1735 (C=O), 1520 (FAR). ^1^H NMR (CDCl_3_): *δ* 5.50 (q, 1H, *J*_H,F_ ~ 6.7 Hz, CH), 3.00 (br.s, 1H, OH). ^19^F NMR (CDCl_3_): *δ* −118.1 (dd, 1F, *J*_A2,B2_ = 290 Hz, *J*_F-F_ = 16 Hz, F_A_-2), −134.5 (dm, 1F, *J*_A2,B2_ = 290 Hz, F_B_-2), −120.9 (dm, 1F, *J*_A3,B3_ = 273 Hz, F_A_-3), −131.6 (dm, 1F, *J*_A3,B3_ = 273 Hz, F_B_-3), −134.8 (ddd, 1F, *J*_8,7_ = 19.8 Hz, *J*_8,5_ = 13.3 Hz, *J*_8,6_ = 13.3 Hz, F-8), −139.4 (ddd, 1F, *J*_5,6_ = 21 Hz, *J*_5,8_ = 13.3 Hz, *J*_5,7_ = 5.9 Hz, F-5), −140.7 (ddd, 1F, *J*_6,5_ = 21 Hz, *J*_6,7_ = 20 Hz, *J*_6,8_ = 13.3 Hz, F-6), −150.1 (ddd, 1F, *J*_7,6_ = 20 Hz, *J*_7,8_ = 19.8 Hz, *J*_7,5_ = 5.9 Hz, F-7). Anal. Calcd for C_10_H_2_F_8_O_2_: C, 39.24%; H, 0.66%; F, 49.65%. Found: C, 39.17%; H, 0.67%; F, 49.63%. HRMS *m/z*, 305.9920 (M^+^). Calcd. M = 305.9922. 

### 3.31. Synthesis of Diols ***17c***, ***17f***, and ***17g***

*a.* A mixture of tetrafluorophthalic acid (3.000 g, 12.61 mmol) with PCl_5_ (6.600 g, 31.65 mmol) was heated (2 h, 180 °C) with distillation of volatile products. Organic products were dissolved in hexane (2 × 10 mL) and filtered. The solvent was evaporated; the residue was dissolved in Et_2_O (10 mL) and added dropwise to a mixture of LiBH_4_ (1.200 g, 55.05 mmol) and Et_2_O (20 mL) cooled on ice. The obtained mixture was stirred at r.t. for 2 h, poured into 5% hydrochloric acid (80 mL), and extracted with Et_2_O (4 × 20 mL). The extract was washed with a 10% aqueous solution of K_2_CO_3_ (80 mL) and dried over MgSO_4_. Evaporation of the solvent and sublimation (130−140 °C, 1 Torr) gave 2.000 g of compound **17c** (yield 76%).

*b.* An analogous procedure for tetrafluoroisophthalic acid afforded compound **17f** (yield 63%).

*c.* An analogous procedure for tetrafluoroterephthalic acid produced compound **17g** (yield 64%).

### 3.32. Synthesis of Diols ***17d*** and ***17e***

*a.* A solution of 4,5,6,7-tetrafluoro-3-(pentafluorophenyl)phthalide (**28**) (1.430 g, 3.84 mmol) in Et_2_O (15 mL) was added to a mixture of LiBH_4_ (0.344 g, 15.78 mmol) and Et_2_O (10 mL). The resulting mixture was stirred at r.t. for 8.5 h, poured into 5% hydrochloric acid (20 mL), extracted with Et_2_O (3 × 10 mL), and the extract was dried over MgSO_4_. Evaporation of the solvent and sublimation (170 °C, 1 Torr) gave 1.342 g of compound **17d** (yield 93%).

*b.* A solution of 4,5,6,7-tetrafluoro-3-(trifluoromethyl)phthalide (**29**) (0.818 g, 2.96 mmol) in Et_2_O (8 mL) was introduced into a mixture of LiBH_4_ (0.300 g, 13.76 mmol) and Et_2_O (4 mL). The obtained mixture was stirred at r.t. for 6 h, poured into 5% hydrochloric acid (10 mL), extracted with Et_2_O (3 × 5 mL), and the extract was dried over MgSO_4_. Evaporation of the solvent and sublimation (100 °C, 1 Torr) resulted in 0.682 g of compound **17e** (yield 82%).

*2,2,2-Trifluoro-1-(2,3,4,5-tetrafluoro-6-(hydroxymethyl)phenyl)ethan-1-ol (***17e***).* White solid, mp 94.9–96.9 °C. IR (KBr) ν, cm^−1^: 3201 (OH), 2928 (CH), 1520, 1485 (FAR). ^1^H NMR (CO(CD_3_)_2_): *δ* 6.56 (d, 1H, *J*_OH,CH_ = 6.7 Hz, CHOH), 5.84 (qd, 1H, *J*_CH,CF3_ = 7.8 Hz, *J*_CH,OH_ = 6.7 Hz, CH), 4.91 (m, 1H, *J*_AB_ = 12.5 Hz, H_A_-CH_2_), 4.76 (m, 1H, *J*_AB_ = 12.5 Hz, H_B_-CH_2_), 4.49 (t, 1H, *J*_OH,CH2_ = 6 Hz, CH_2_OH). ^19^F NMR (CO(CD_3_)_2_): *δ* −75.7 (dd, 3F, *J*_CF3,F(2)_ = 12.3 Hz, *J*_CF3,CH_ = 7.8 Hz, CF_3_), −137.7 (dqdd, 1F, *J*_2,3_ = 20.3 Hz, *J*_F(2),CF3_ = 12.3 Hz, *J*_2,5_ =12.1 Hz, *J*_2,4_ = 4.3 Hz, F-2), −142.2 (ddd, 1F, *J*_5,4_ = 21.3 Hz, *J*_5,2_ = 12.1 Hz, *J*_5,3_ = 3 Hz, F-5), −154.8 (ddd, 1F, *J*_4,5_ = 21.3 Hz, *J*_4,3_ = 20.3 Hz, *J*_4,2_ = 4.3 Hz, F-4), −156.8 (ddd, 1F, *J*_3,2_ = 20.3 Hz, *J*_3,4_ = 20.3 Hz, *J*_3,5_ = 3 Hz, F-3). Anal. Calcd for C_9_H_5_F_7_O_2_: C, 39.26; H, 1.88; F, 47.91%. Found: C, 38.87; H, 1.81; F, 47.82%. HRMS m/z, 278.0176 (M^+^). Calcd. M = 278.0172.

## Figures and Tables

**Table 1:** Carbonylation of primary and secondary alcohols C_6_F_5_CH(OH)R **1a**–**c** in superacids.

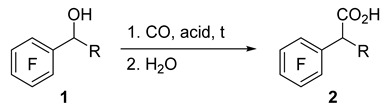								
Entry	Substrate, R		Acid	Quantity of Acid, Equiv.		t, °C	Time, h	Content (Yield) of Acid 2, %
				RSO_3_H	SbF_5_			
1	**1a**	H	TfOH	5.5	-	50	6	99 (90)
2	**1b**	C_6_F_5_	TfOH	7	-	r.t.	3	99 (97)
3	**1c**	CF_3_	TfOH	5	-	50	11	-
4	“	“	TfOH-SbF_5_	2	2	r.t.	2	95
5	“	“	TfOH-SbF_5_	2	2	r.t.	20	99 (91)
6	“	“	TfOH-SbF_5_	1.5	1.5	r.t.	21	20
7	“	“	FSO_3_H-SbF_5_	2	2	r.t.	2	99

**Table 2:** Carbonylation of tertiary perfluorinated 1,1-diarylalkan-1-ols **1d**–**f** in superacids.

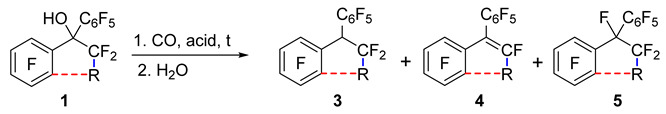											
Entry	Substrate	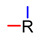	Acid	Quantity of Acid, Equiv.		t, °C	Time, h	Content of Products, %			
				RSO_3_H	SbF_5_			3	4	5	1
1	**1d**	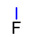	TfOH-SbF_5_	2	2	r.t.	5	18	12	65	-
2	“	“	TfOH-SbF_5_	2	2	r.t.	24	22	11	60	-
3	“	“	TfOH-SbF_5_	4	4	r.t.	24	8	4	63	21
4	“	“	TfOH-SbF_5_	1.2	1.2	r.t.	24	6	3	75	5
5	“	“	FSO_3_H-SbF_5_	2	2	r.t.	3	36	8	50	1
6	**1e**	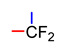	FSO_3_H-SbF_5_	2	2	r.t.	3	47	10	4	35
7	**1f**	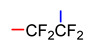	FSO_3_H-SbF_5_	2	2	r.t.	3	8	14; 20 ^a^	41	13
8	“	“	FSO_3_H-SbF_5_	4	4	50	10	1	2; 47 ^a^	27	15

^a^ Content of lactone **8**, see Scheme 2.

**Table 3:** Carbonylation of secondary polyfluorinated 1-arylalkan-1-ols **1g**–**n** in superacids.

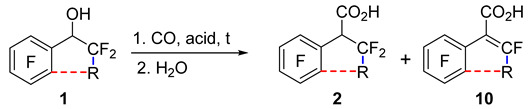									
Entry	Substrate	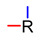	Acid	Quantity of Acid, Equiv.		t, °C	Time, h	Content (Yield) of Products, %	
				RSO_3_H	SbF_5_			2	10
1	**1g**	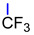	TfOH-SbF_5_	2	2	r.t.	2	98 (88)	2
2	“	“	TfOH-SbF_5_	3	3	70	5.5	42	52
3	“	“	FSO_3_H-SbF_5_	3	3	70	5.5	13	80 (73) ^a^ E:Z = 15:85
4	**1c**	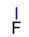	FSO_3_H-SbF_5_	3	3	70	7	93	<2
5	**1h**	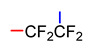	FSO_3_H-SbF_5_	2	2	r.t.	2	69 (35)	23
6	“	“	FSO_3_H-SbF_5_	2	2	r.t.	24	61	25 (80) ^a^
7	“	“	FSO_3_H-SbF_5_	4	4	r.t.	20	48	45
8	**1i**	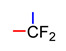	FSO_3_H-SbF_5_	2	2	r.t.	2.5	50 + 5 of 2j	-
9	“	“	FSO_3_H-SbF_5_	3	3	70	6	63 (40) of 2j	34 (25) of 10j
10	**1j**	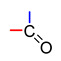	FSO_3_H-SbF_5_	3.5	3.5	70	9.5	35	55
11	**1k**	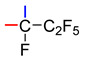	FSO_3_H-SbF_5_	2	2	r.t.	2.5	99 (82) isomers 67:33	-
12	**1l**	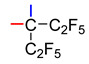	FSO_3_H-SbF_5_	2	2	r.t.	3	99 (71)	-
13	“	“	FSO_3_H-SbF_5_	3	3	70	6.5	71	27
14	**1m**	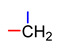	FSO_3_H-SbF_5_	2	2	r.t.	1.5	~70 ^b^	-
15	“	“	TfOH-SbF_5_	5	1	50	1.5	97 (95)	-
16	**1n**	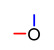	FSO_3_H-SbF_5_	2	2	r.t.	2	-	90 (71)

^a^ The yield of the mixture of acids **2** and **10**. ^b^ The reaction is accompanied by substantial resinification.

**Table 4:** Carbonylation of polyfluorinated diols **17c**–**g** in superacids.

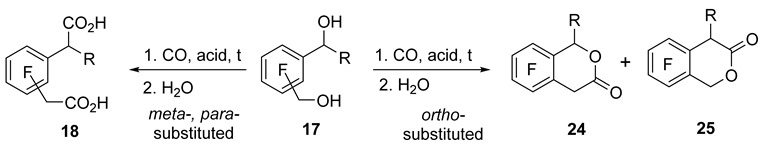											
Entry	Substrate	Substitution Pattern	R	Acid	Quantity of Acid, Equiv.		t, °C	Time, h	Content (Yield) of Products, %		
					RSO_3_H	SbF_5_			18	24	25
1	**17c**	*ortho*	H	TfOH	7	-	70	4	-	25	
2	“	“	“	TfOH-SbF_5_	7.2	1.2	r.t	2	-	78 (55)	
3	“	“	“	FSO_3_H-SbF_5_	4	4	r.t	4	-	~25 ^a^	
4	**17d**	*ortho*	C_6_F_5_	FSO_3_H-SbF_5_	4	4	r.t	4	-	50	45 (73) ^b^
5	**17e**	*ortho*	CF_3_	FSO_3_H-SbF_5_	4	4	50	7	-	65 (32)	-
6	**17f**	*meta*	H	TfOH-SbF_5_	7	1	r.t	5	95 (69)	-	-
7	**17g**	*para*	H	FSO_3_H-SbF_5_	5	5	r.t	6.5	80 (55)	-	-

^a^ The reaction entails substantial resinification. ^b^ The yield of a mixture of lactones **24d** and **25d**.

## Data Availability

All raw data of this study are available within this article.

## Supplementary Materials

The following supporting information can be downloaded at: https://www.mdpi.com/article/10.3390/molecules27248757/s1, Figures S1−S73: NMR spectra of the obtained compounds.
