# Supplementary material for: Carbonylation of Polyfluorinated 1-Arylalkan-1-ols and Diols in Superacids
Source: Molecules. 2022 Dec 10;27(24):8757. doi: 10.3390/molecules27248757 (PMC9787639; doi:10.3390/molecules27248757)
Supplement: Supplementary file 1 [file molecules-27-08757-s001.zip › molecules-2075978-supplementary.pdf]

Supplementary information

Carbonylation of polyfluorinated 1-arylalkan-1-ols and diols in superacids

Siqi Wang [1,2], Yaroslav V. Zonov [1,2], Victor M. Karpov [1], Olga A. Luzina [1,*], Tatyana V. Mezhenkova [1]

[1] N.N. Vorozhtsov Novosibirsk Institute of Organic Chemistry, pr. Akademika Lavrent'eva 9, 630090, Novosibirsk, Russia
[2] Novosibirsk State University, Pirogova str. 1, 630090, Novosibirsk, Russia
* Correspondence: luzina@nioch.nsc.ru

Content page

- ^{19}F and ^{1}H NMR spectra of compound **1j** S2
- ^{19}F and ^{1}H NMR spectra of compound **1o** S3
- ^{19}F and ^{1}H NMR spectra of compound **2b** S4
- ^{19}F and ^{1}H NMR spectra of compound **2c** S5
- ^{19}F and ^{1}H NMR spectra of compound **2cMe** S6
- ^{19}F and ^{1}H NMR spectra of compound **2g** S7
- ^{19}F and ^{1}H NMR spectra of compound **2gMe** S8
- ^{19}F and ^{1}H NMR spectra of compound **2h** S9
- ^{19}F NMR spectrum of compound **2hMe** S10
- ^{19}F and ^{1}H NMR spectra of compound **2iMe** S11
- ^{19}F and ^{1}H NMR spectra of compound **2j** S12
- ^{19}F and ^{1}H NMR spectra of compound **2k** S13
- ^{19}F and ^{1}H NMR spectra of compound **2l** S14
- ^{19}F and ^{1}H NMR spectra of compound **2m** S15
- ^{19}F and ^{1}H NMR spectra of compound **3d** S16
- ^{19}F NMR spectrum of compound **4d** S17
- ^{19}F and ^{13}C NMR spectra of compound **8** S18
- ^{19}F and ^{1}H NMR spectra of compound **10g** S19
- ^{19}F and ^{1}H NMR spectra of compound **10gMe** S20
- ^{19}F and ^{1}H NMR spectra of compound **10hMe** S21
- ^{19}F NMR spectrum of compound **10 h** S22
- ^{19}F and ^{1}H NMR spectra of compound **10iMe** S23
- ^{19}F NMR spectrum of compound **10j** S24
- ^{19}F NMR spectrum of compound **10l** S25
- ^{19}F NMR spectrum of compound **10n** S26
- ^{19}F and ^{1}H NMR spectra of compound **11Me** S27
- ^{19}F and ^{1}H NMR spectra of compound **17e** S28
- ^{19}F and ^{1}H NMR spectra of compound **18a** S29
- ^{19}F and ^{1}H NMR spectra of compound **18b** S30
- ^{19}F and ^{1}H NMR spectra of compound **18bMe** S31
- ^{19}F and ^{1}H NMR spectra of compound **18f** S32
- ^{19}F and ^{1}H NMR spectra of compound **18g** S33
- ^{19}F NMR spectrum of compound **19** S34
- ^{19}F and ^{1}H NMR spectra of compound **19Me** S35
- ^{19}F and ^{1}H NMR spectra of compounds **20Et** and **21Et** S36
- ^{19}F and ^{1}H NMR spectra of compound **22** S37
- ^{19}F and ^{1}H NMR spectra of compound **23** S38
- ^{19}F and ^{1}H NMR spectra of compound **24c** S39
- ^{19}F and ^{1}H NMR spectra of compounds **24d** and **25d** S40
- ^{19}F and ^{1}H NMR spectra of compound **24e** S41

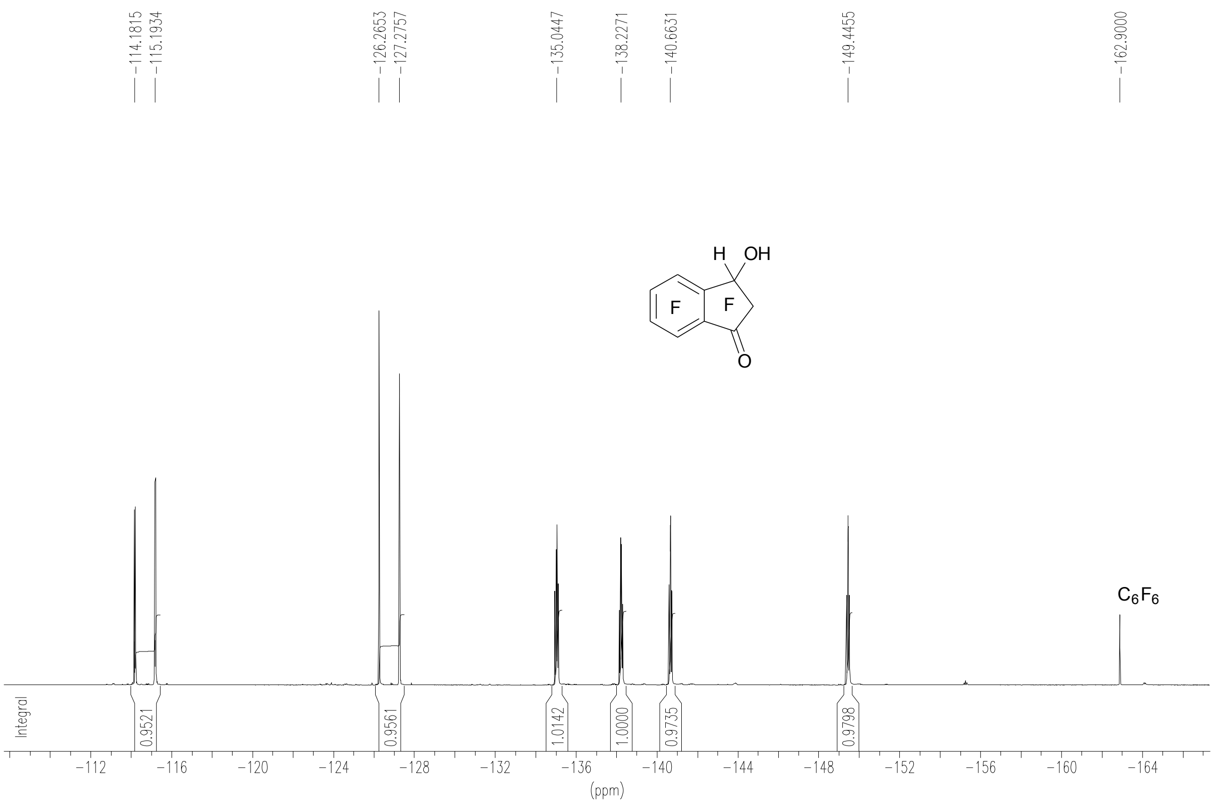

Figure S1: The ^{19}F NMR spectrum of compound **1j** ($CDCl_3$).

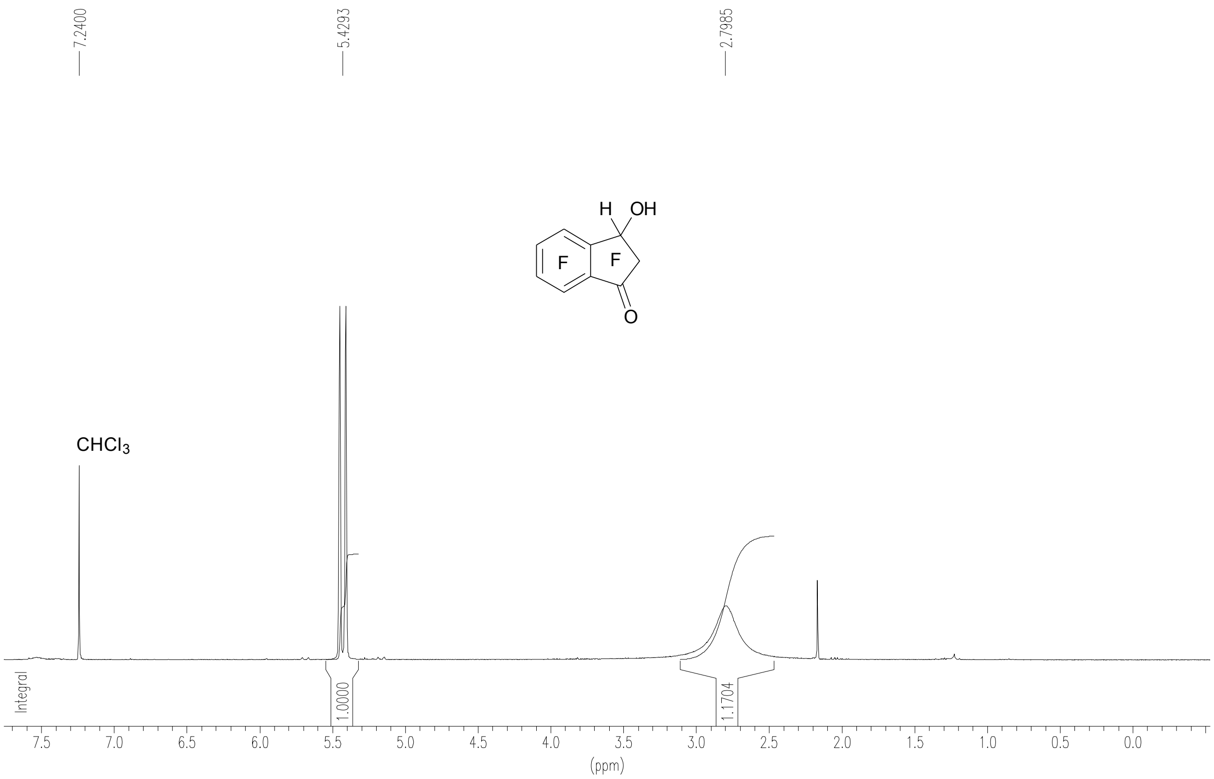

Figure S2: The ^{1}H NMR spectrum of compound **1j** ($CDCl_3$).

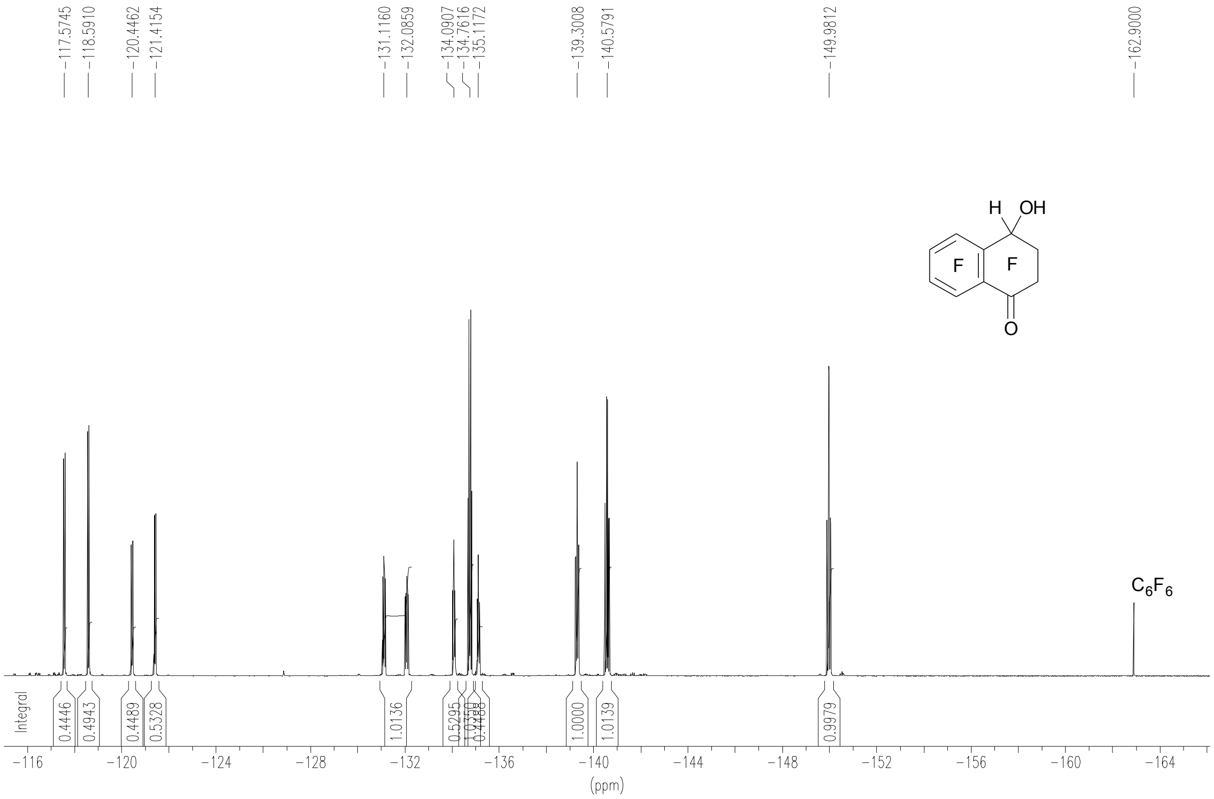

Figure S3: The ^{19}F NMR spectrum of compound **1o** ($CDCl_3$).

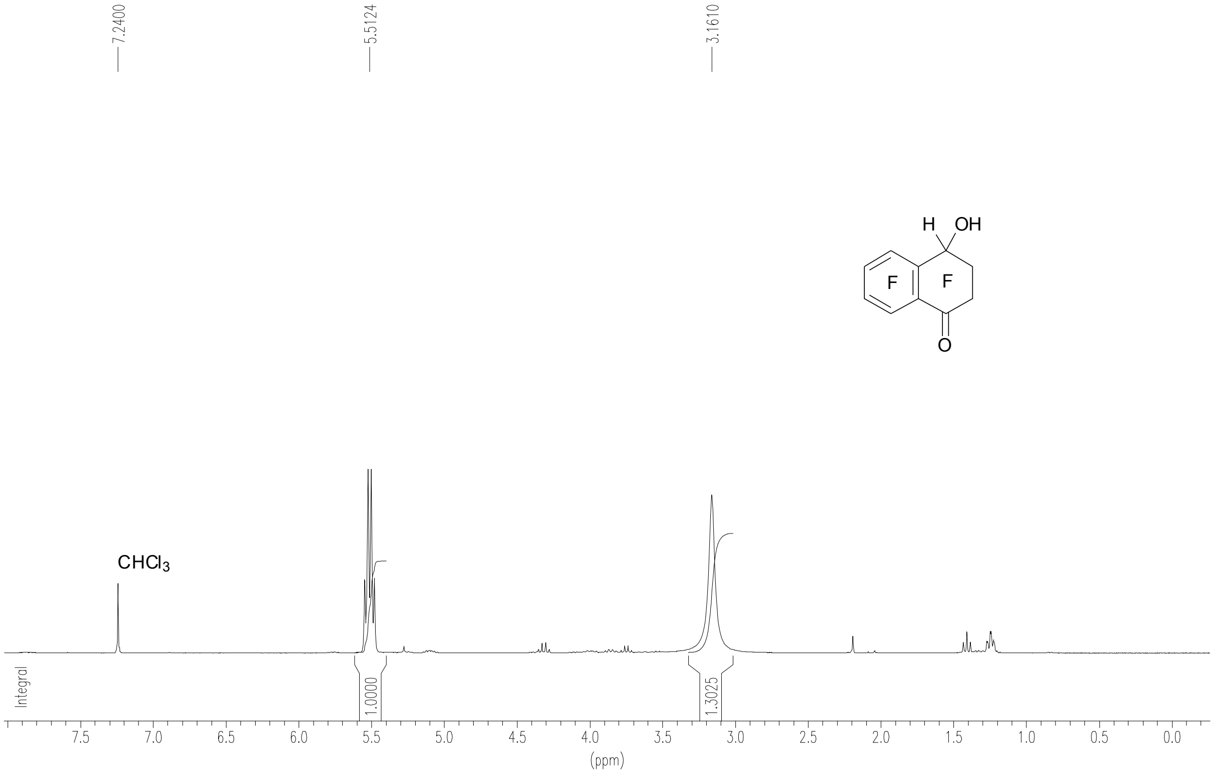

Figure S4: The ^{1}H NMR spectrum of compound **1o** ($CDCl_3$).

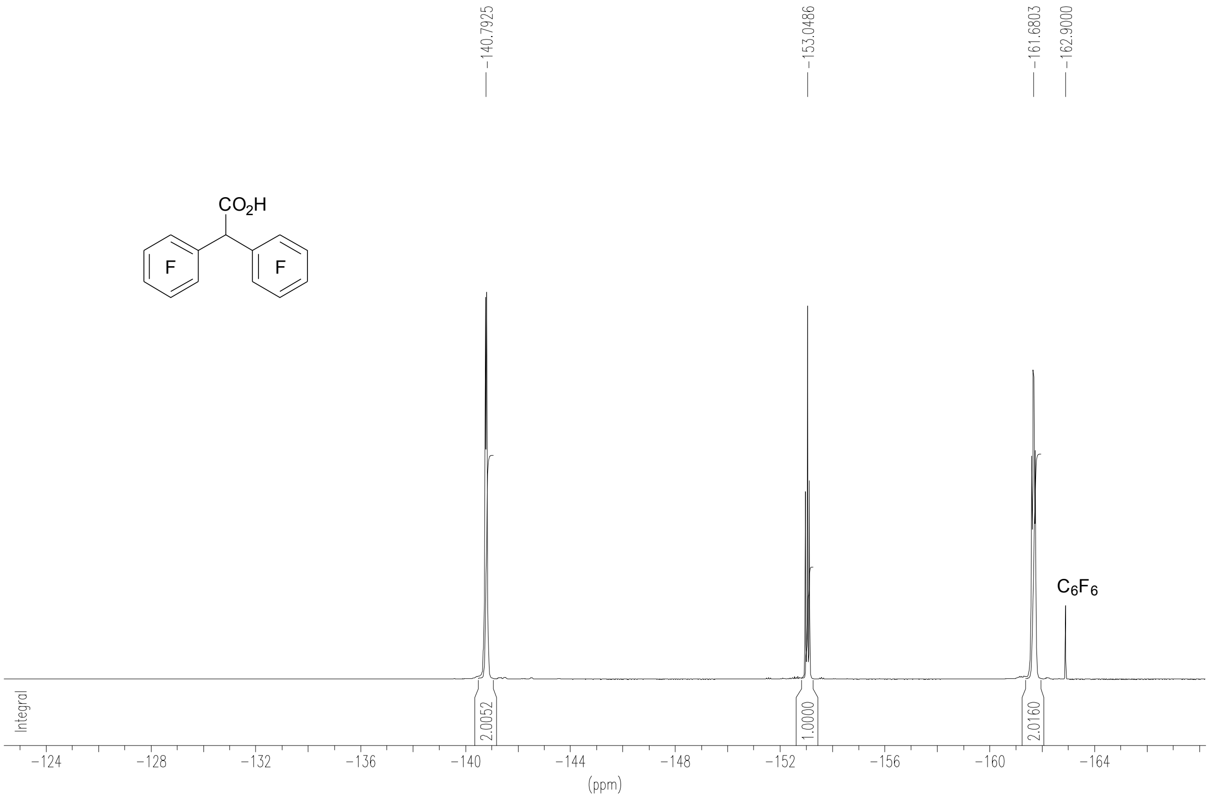

Figure S5: The ^{19}F NMR spectrum of compound **2b** ($CDCl_3$).

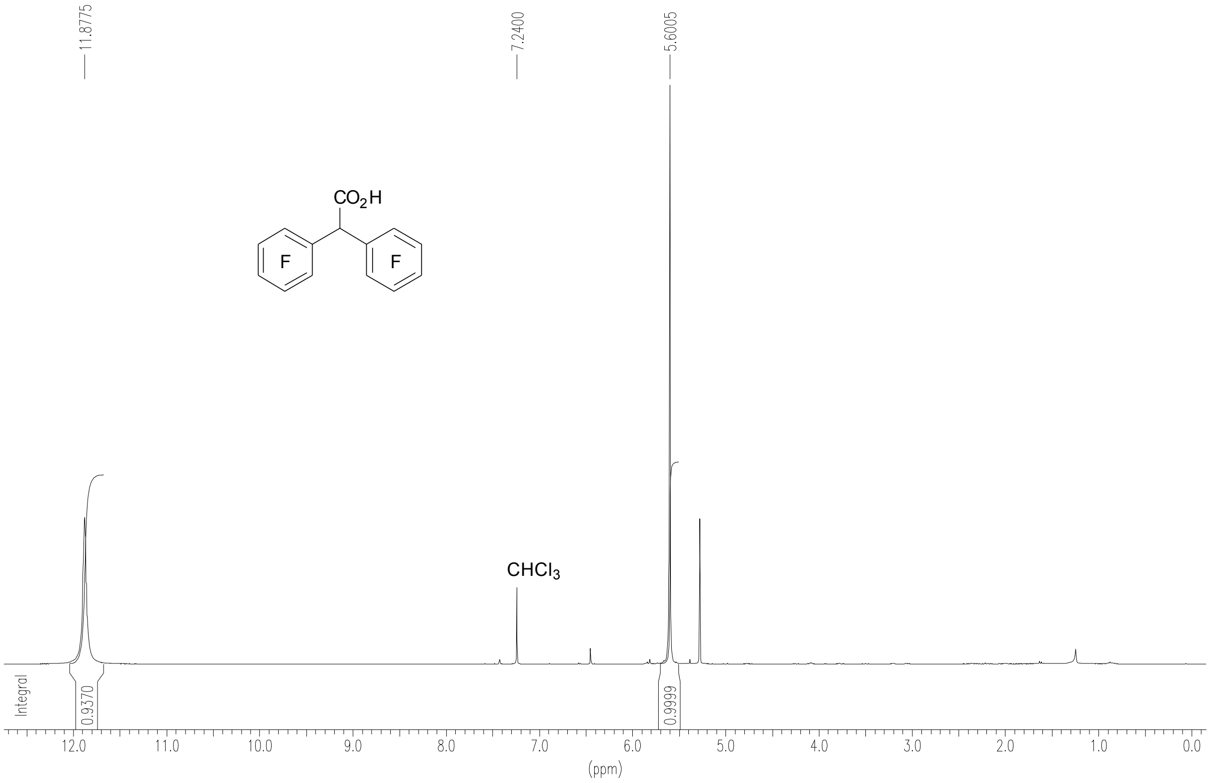

Figure S6: The ^{1}H NMR spectrum of compound **2b** ($CDCl_3$).

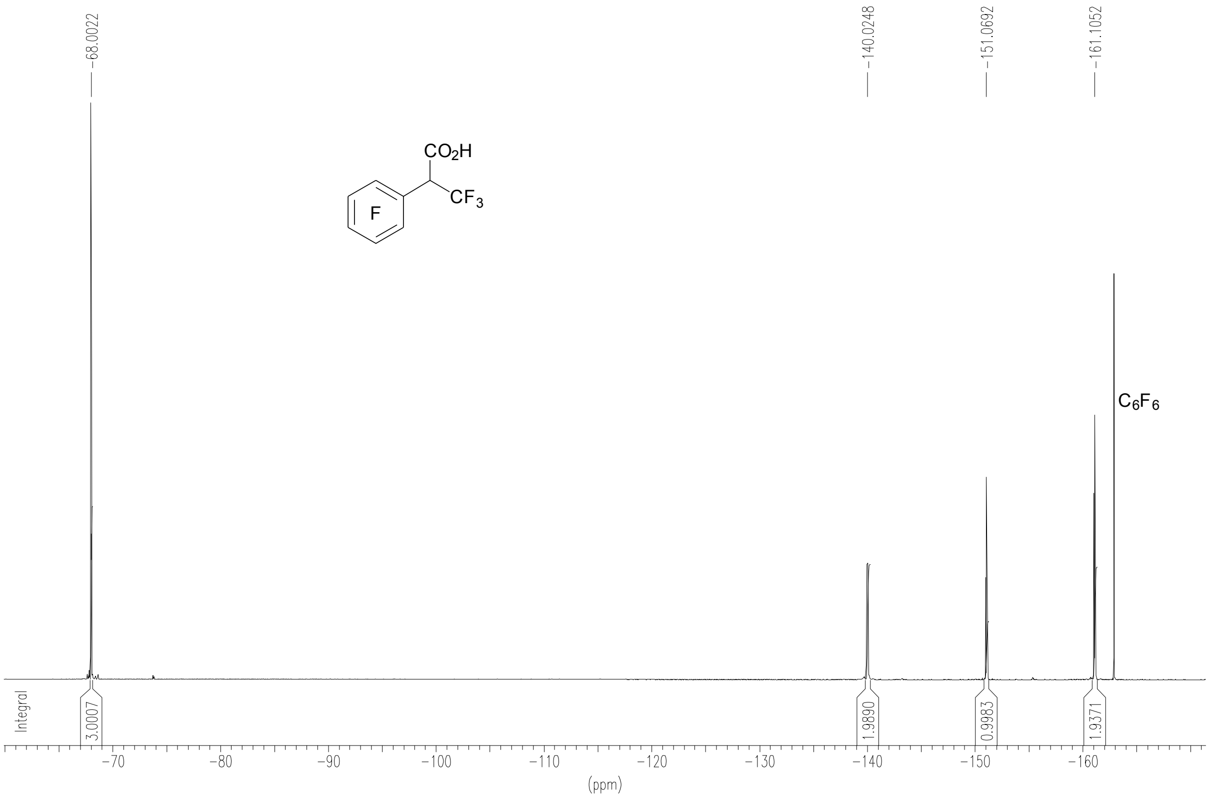

Figure S7: The ^{19}F NMR spectrum of compound **2c** ($CDCl_3$).

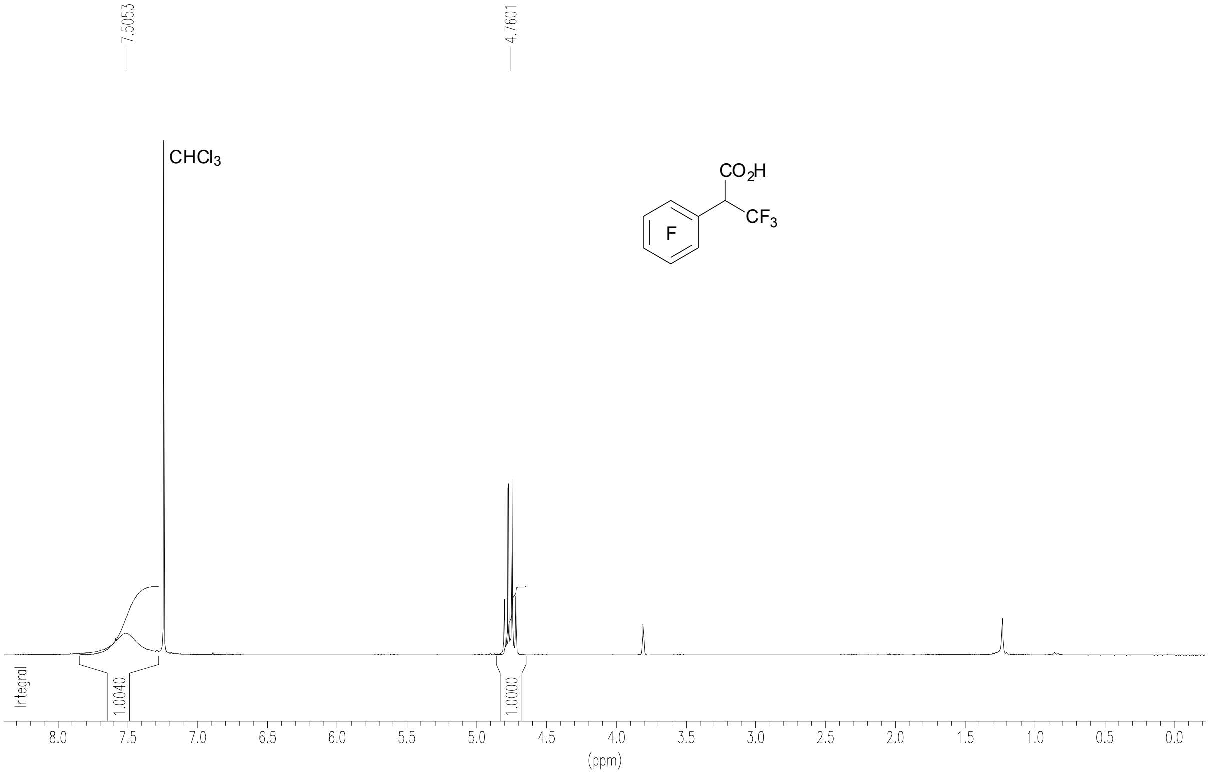

Figure S8: The ^{1}H NMR spectrum of compound **2c** ($CDCl_3$).

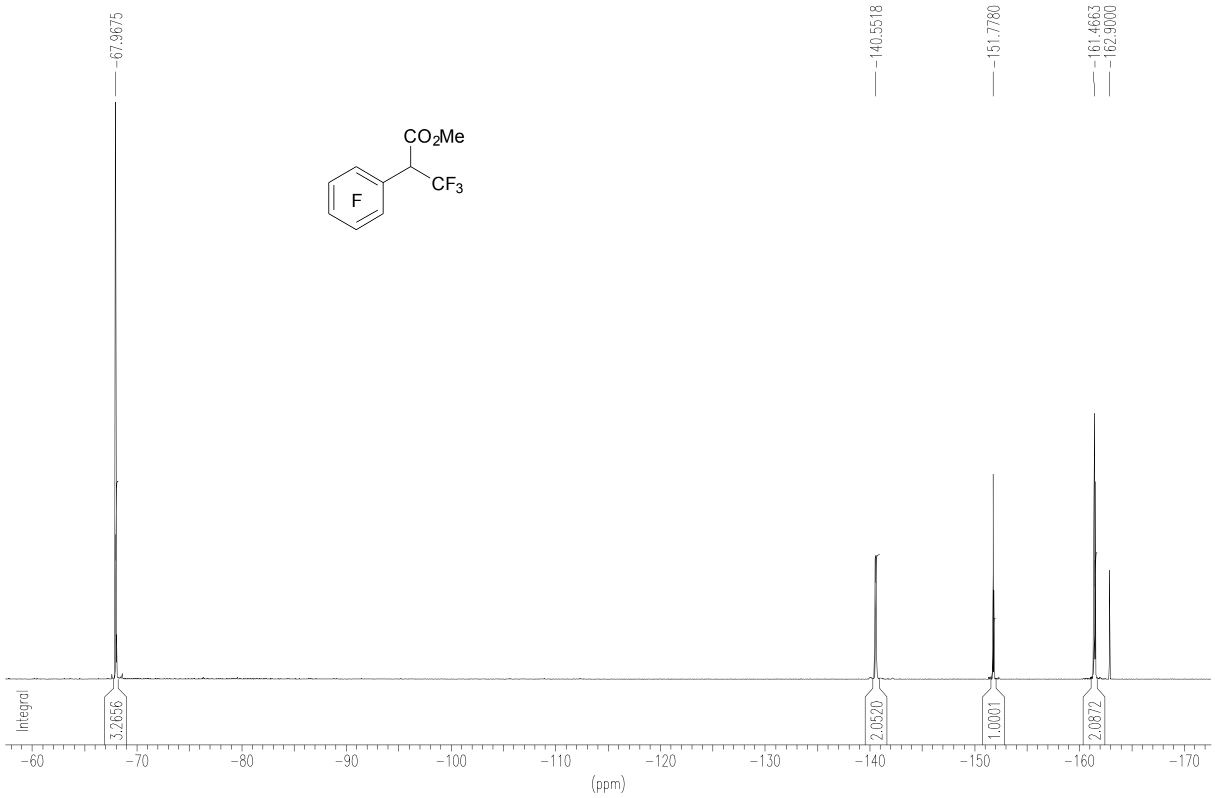

Figure S9: The ^{19}F NMR spectrum of compound **2cMe** ($CDCl_3$).

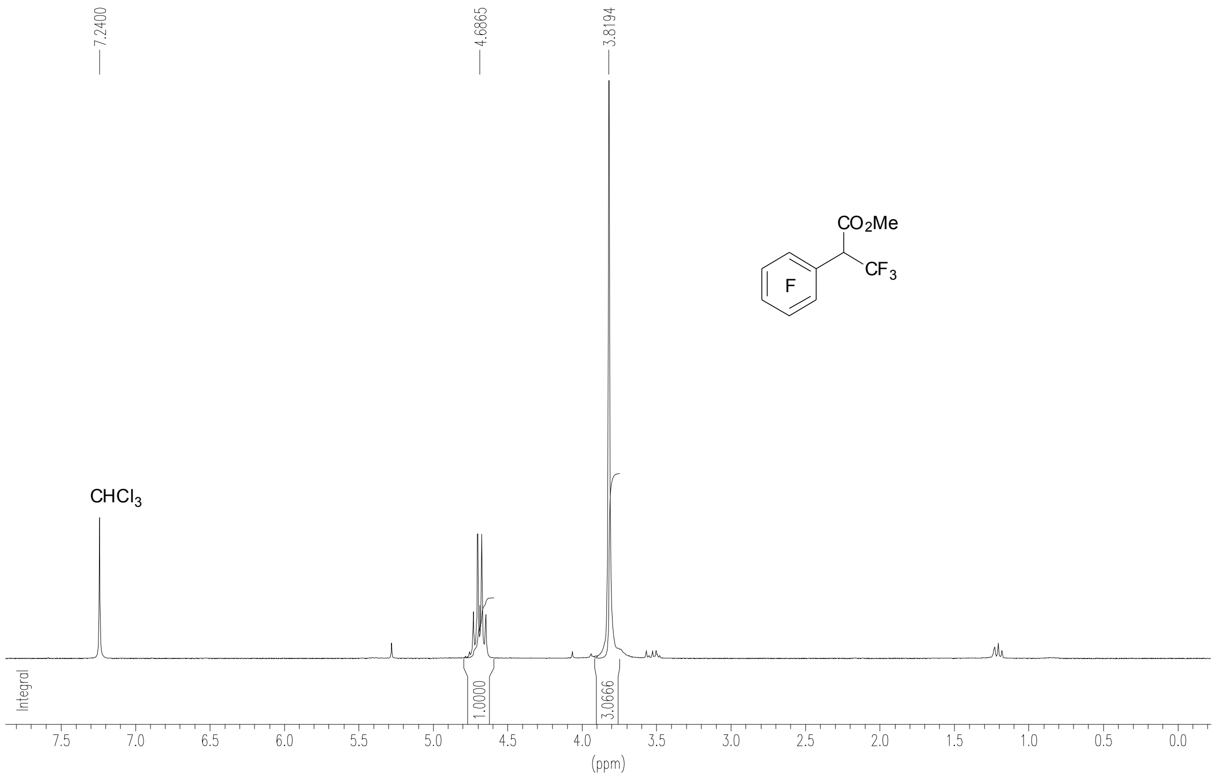

Figure S10: The ^{1}H NMR spectrum of compound **2cMe** ($CDCl_3$).

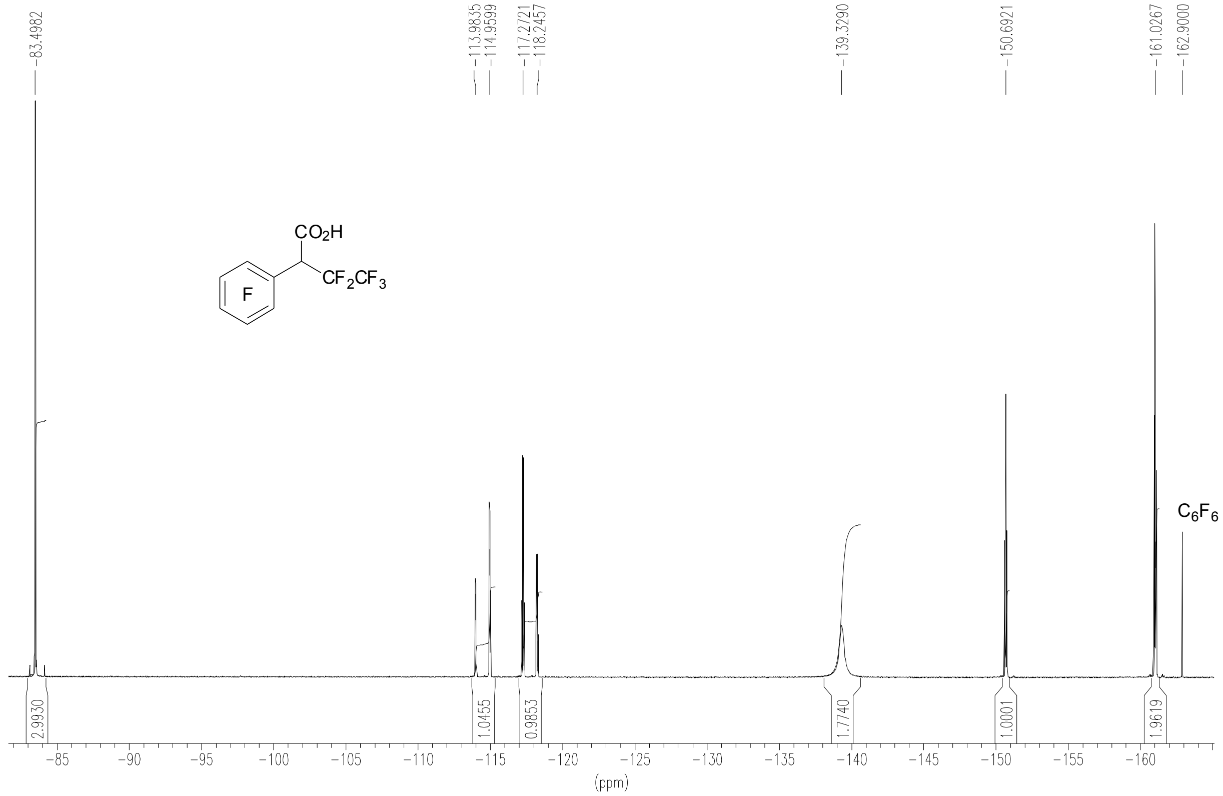

Figure S11: The ^{19}F NMR spectrum of compound **2g** ($CDCl_3$).

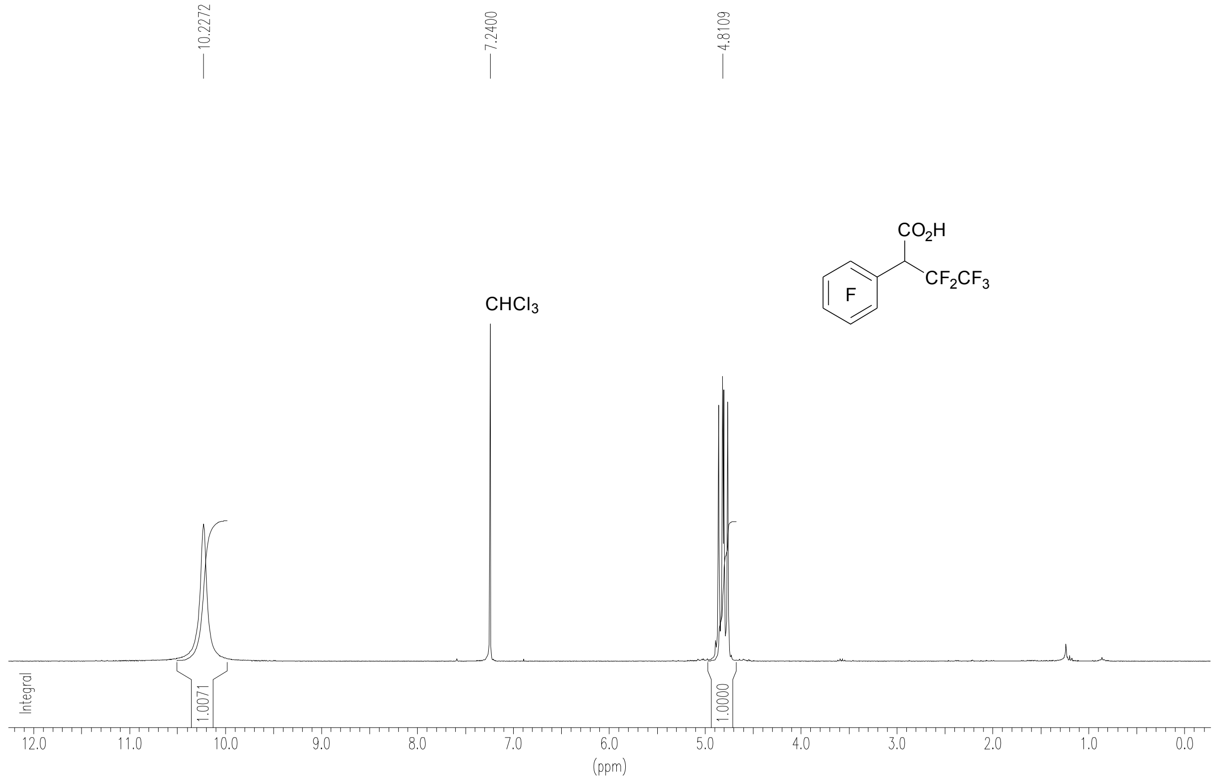

Figure S12: The ^{1}H NMR spectrum of compound **2g** ($CDCl_3$).

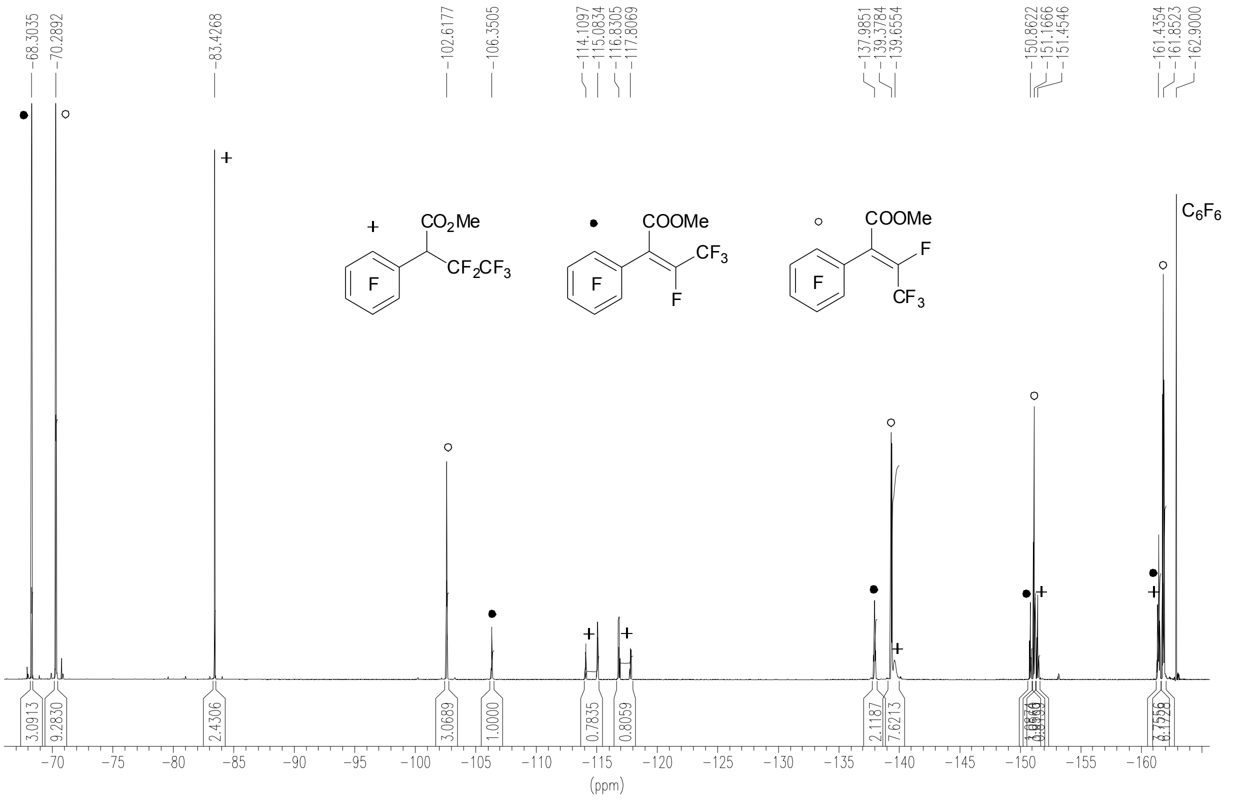

Figure S13: The ^{19}F NMR spectrum of the mixture of compounds **2gMe** and **10gMe** ($CDCl_3$).

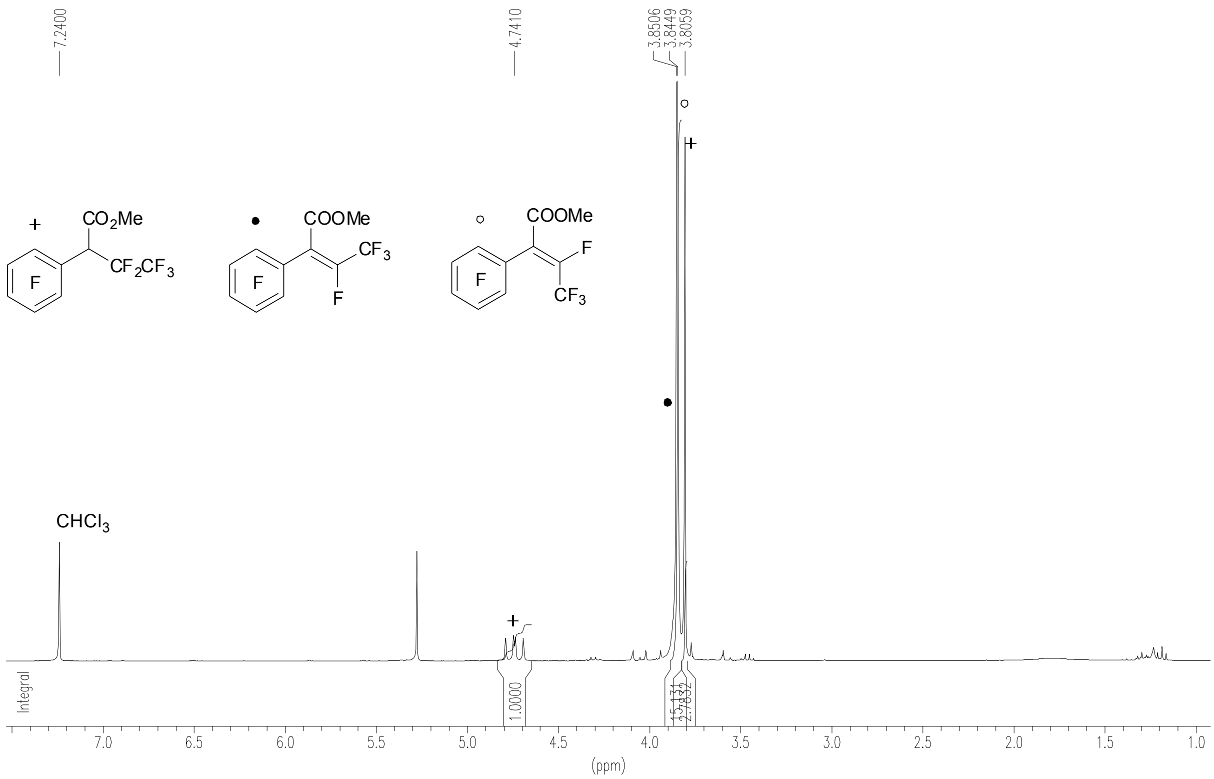

Figure S14: The ^{1}H NMR spectrum of the mixture of compounds **2gMe** and **10gMe** ($CDCl_3$).

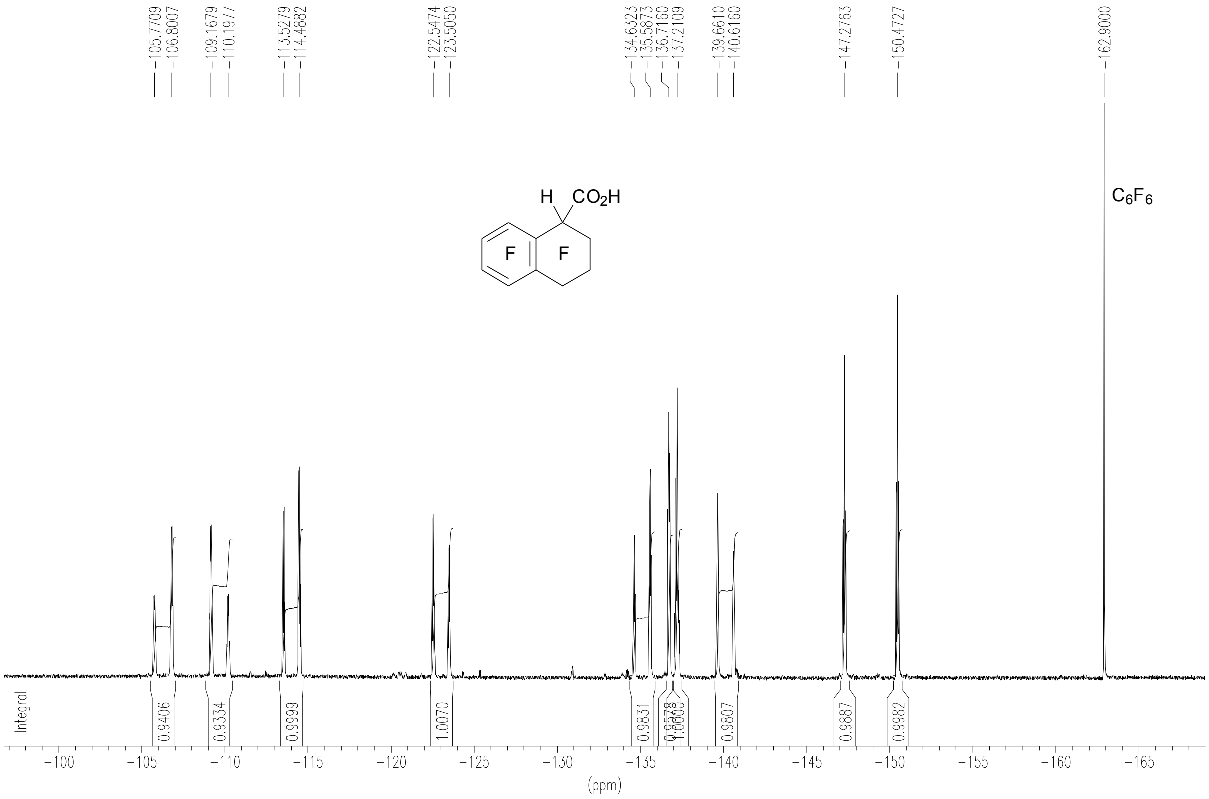

Figure S15: The ^{19}F NMR spectrum of compound **2h** ($CDCl_3$).

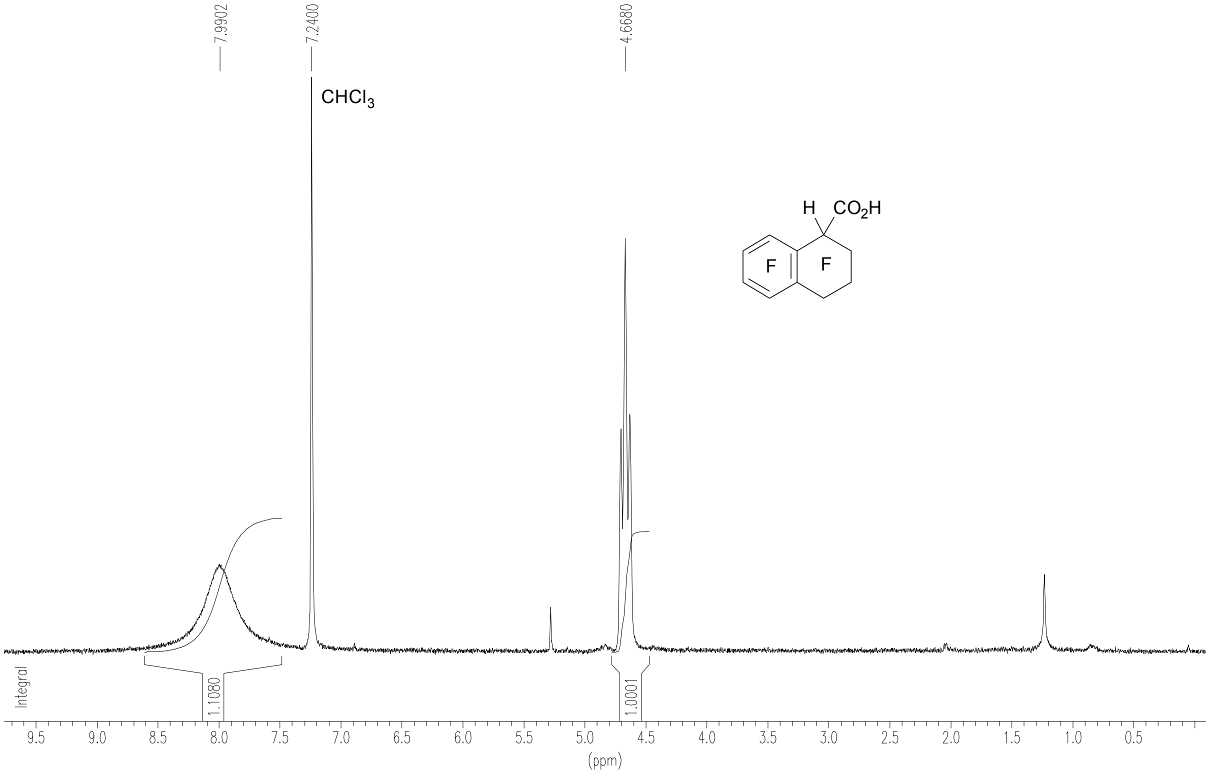

Figure S16: The ^{1}H NMR spectrum of compound **2h** ($CDCl_3$).

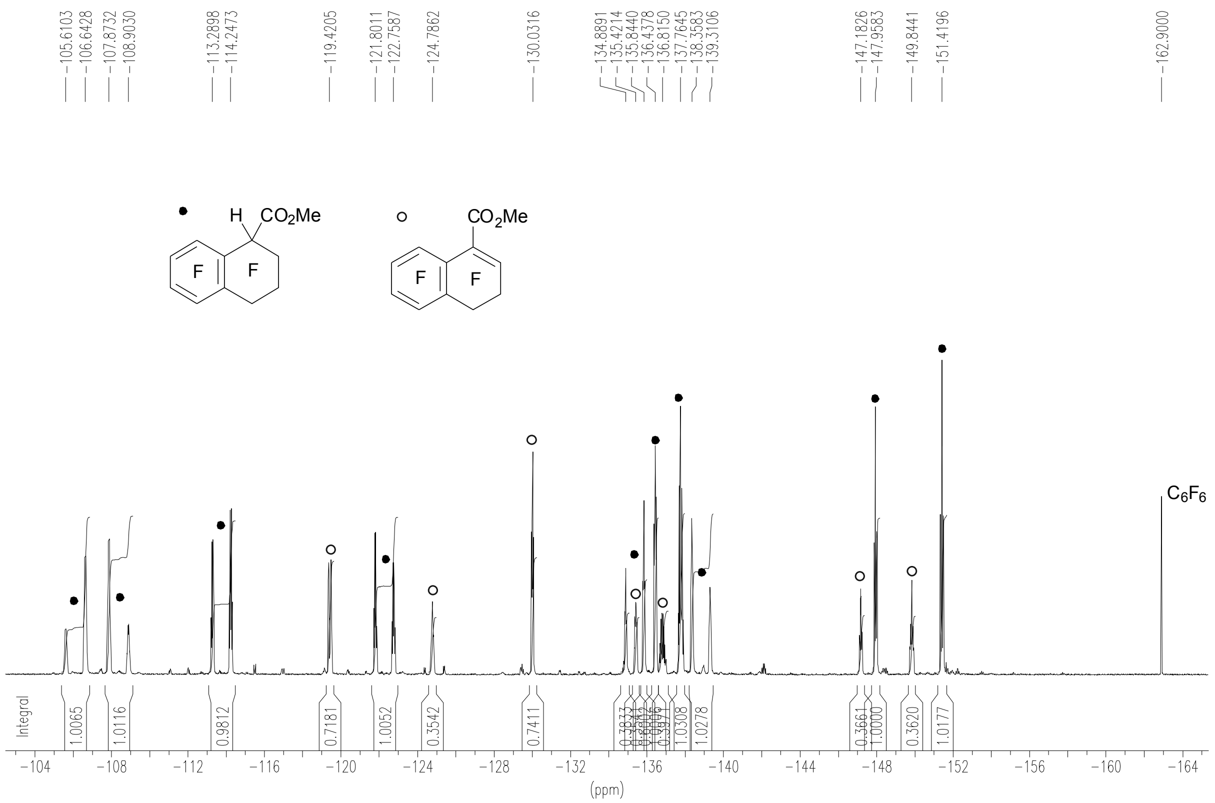

Figure S17: The ^{19}F NMR spectrum of the mixture of compounds **2hMe** and **10hMe** ($CDCl_3$).

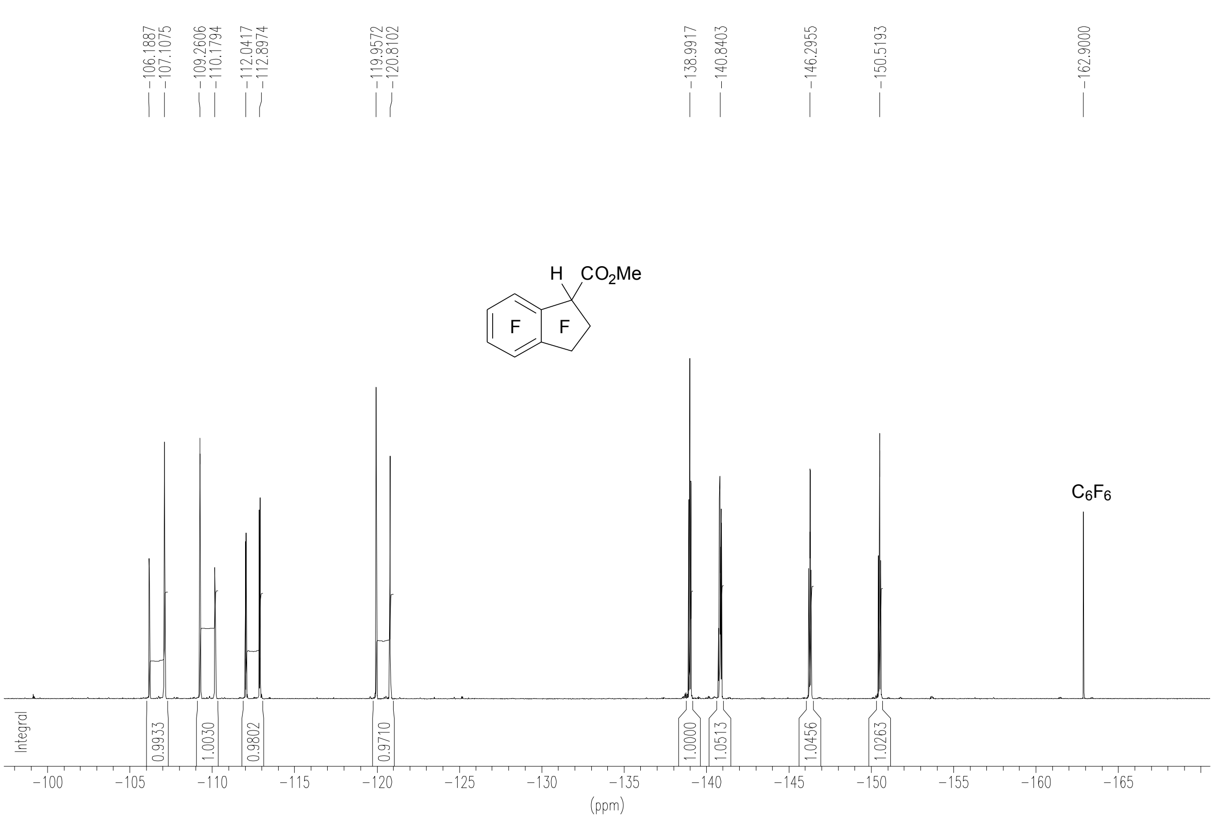

Figure S18: The ^{19}F NMR spectrum of compound **2iMe** ($CDCl_3$).

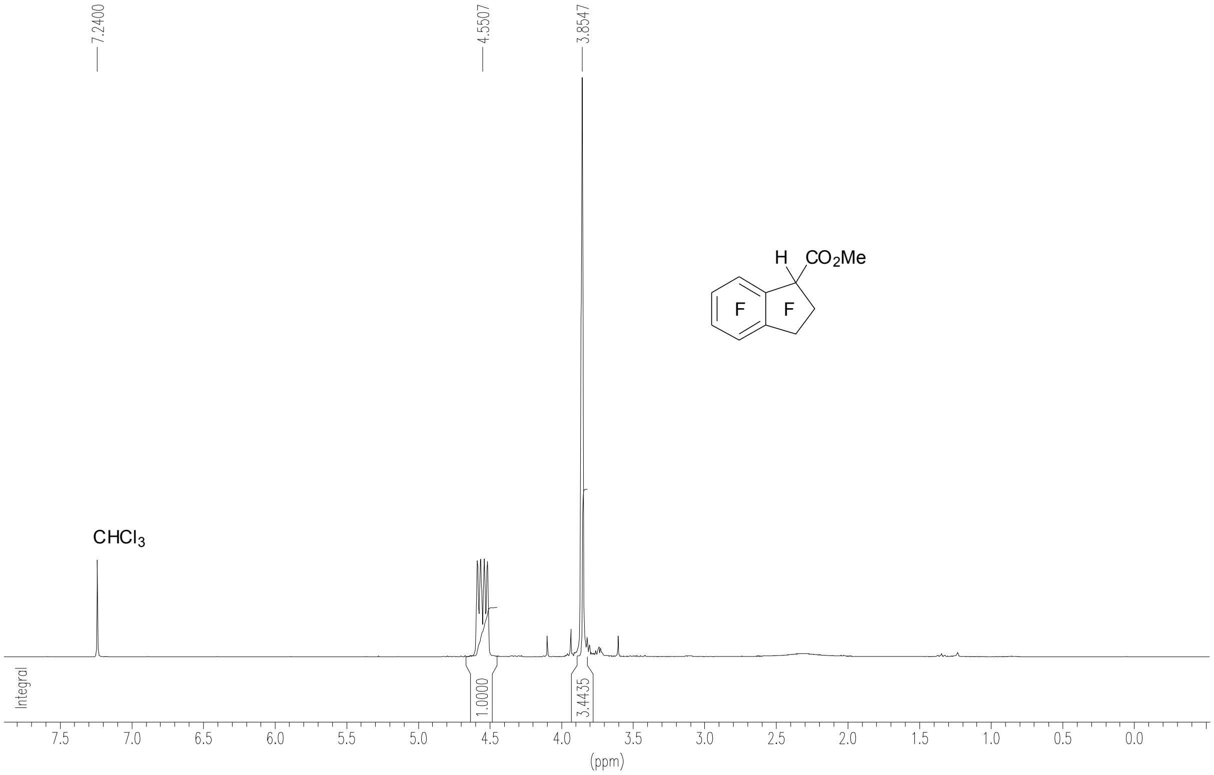

Figure S19: The ^{1}H NMR spectrum of compound **2iMe** ($CDCl_3$).

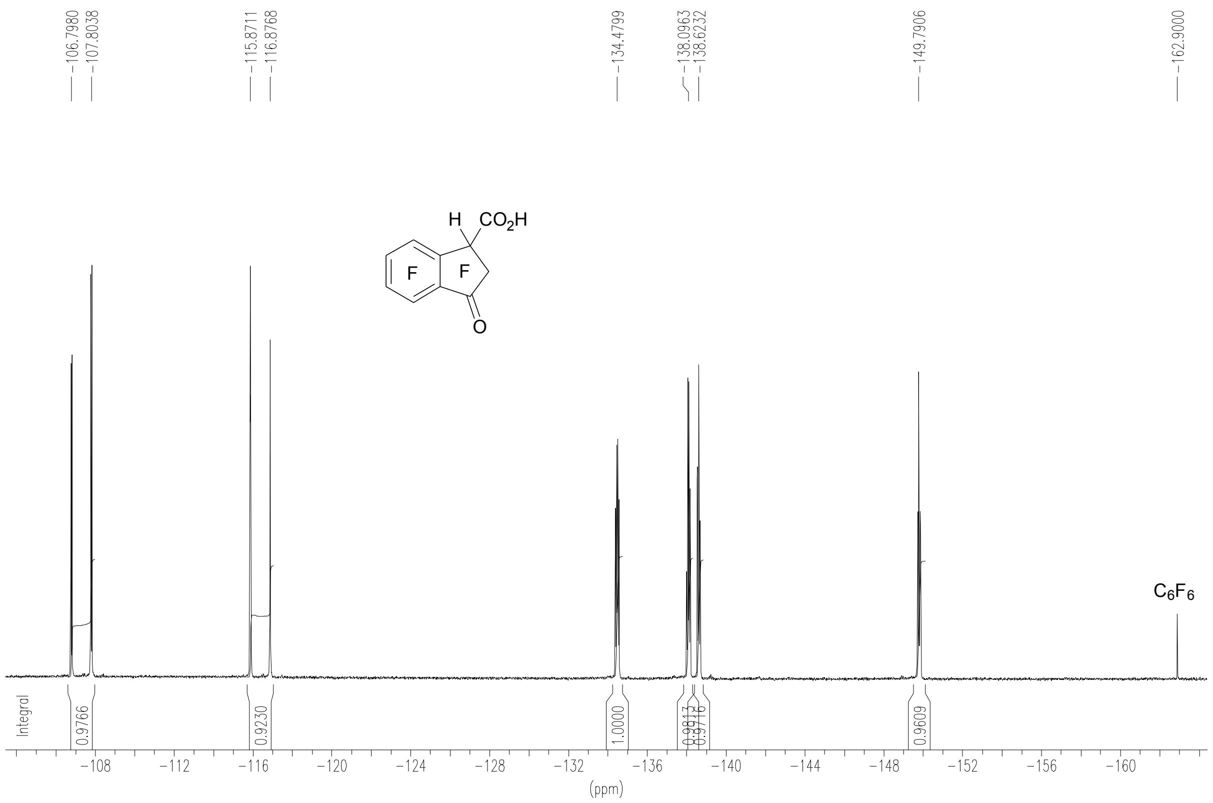

Figure S20: The ^{19}F NMR spectrum of compound **2j** ($CDCl_3$).

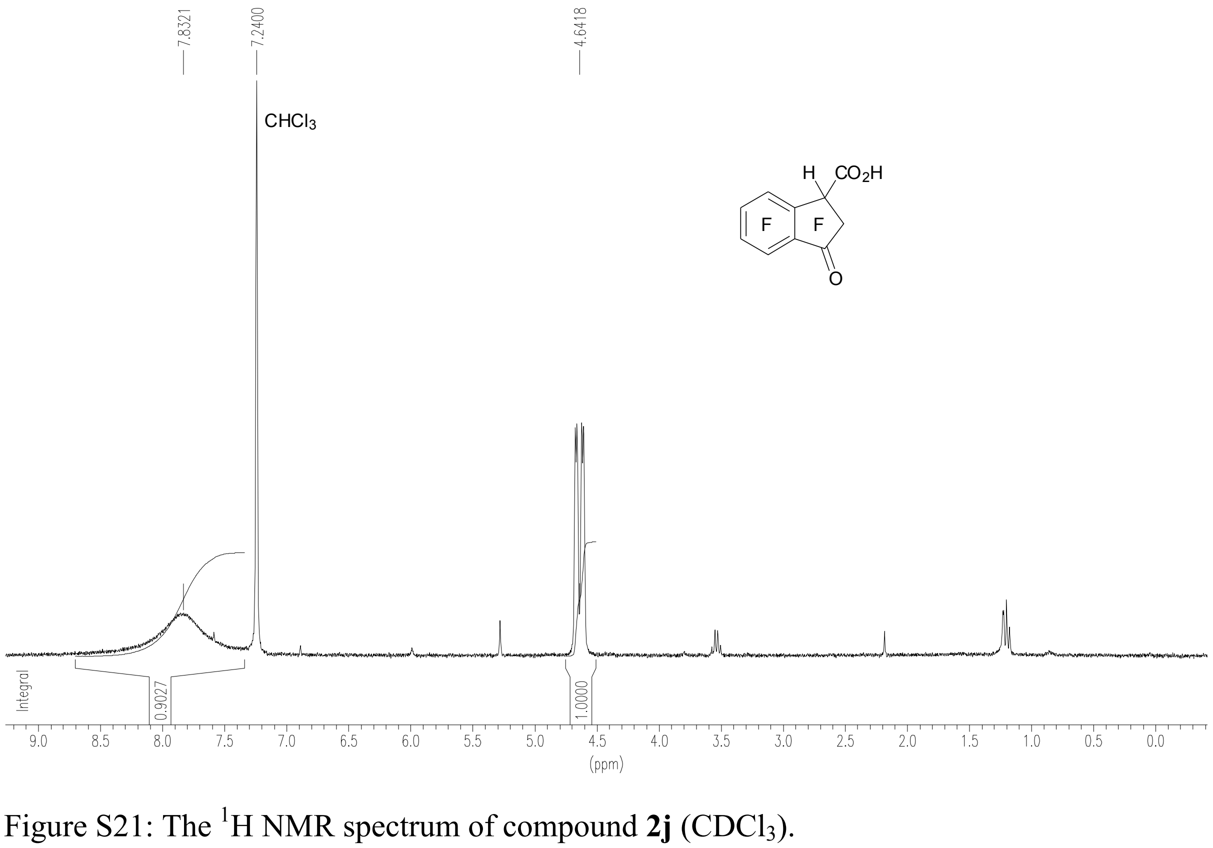

Figure S21: The ^{1}H NMR spectrum of compound **2j** ($CDCl_3$).

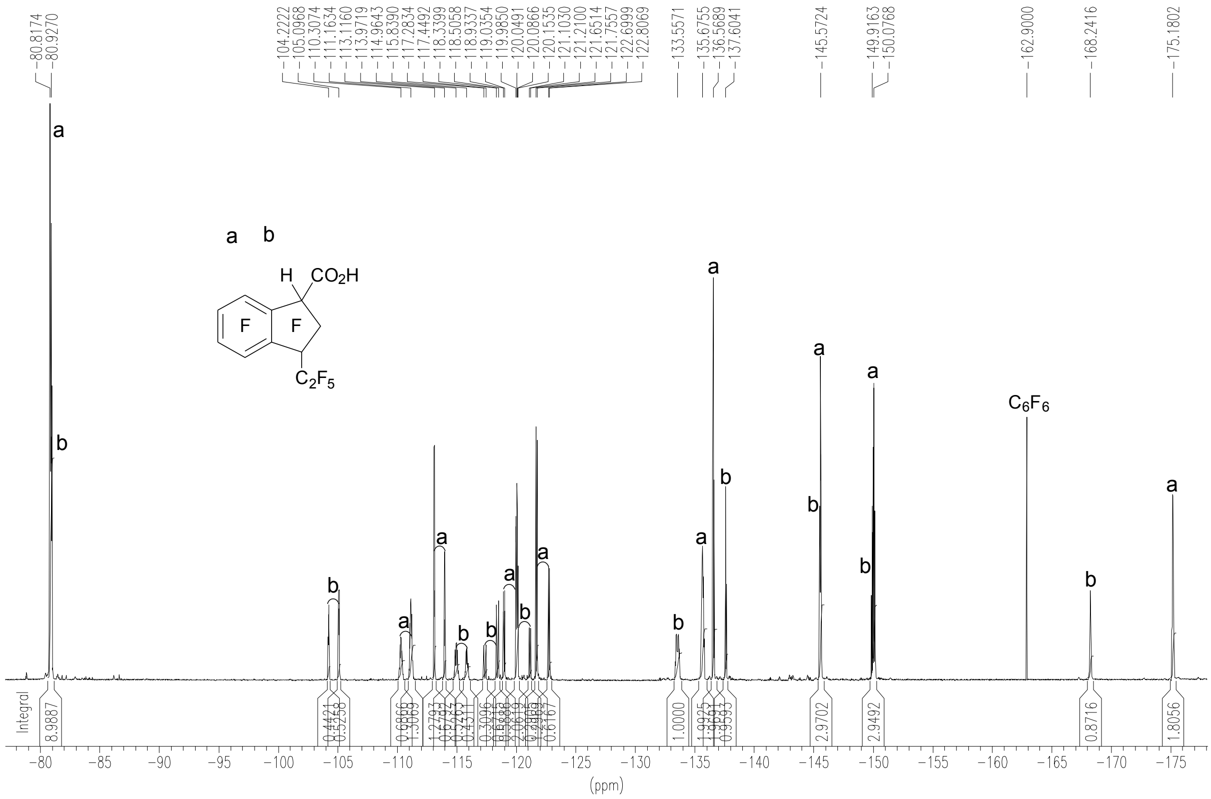

Figure S22: The ^{19}F NMR spectrum of compound **2k** ($CDCl_3$).

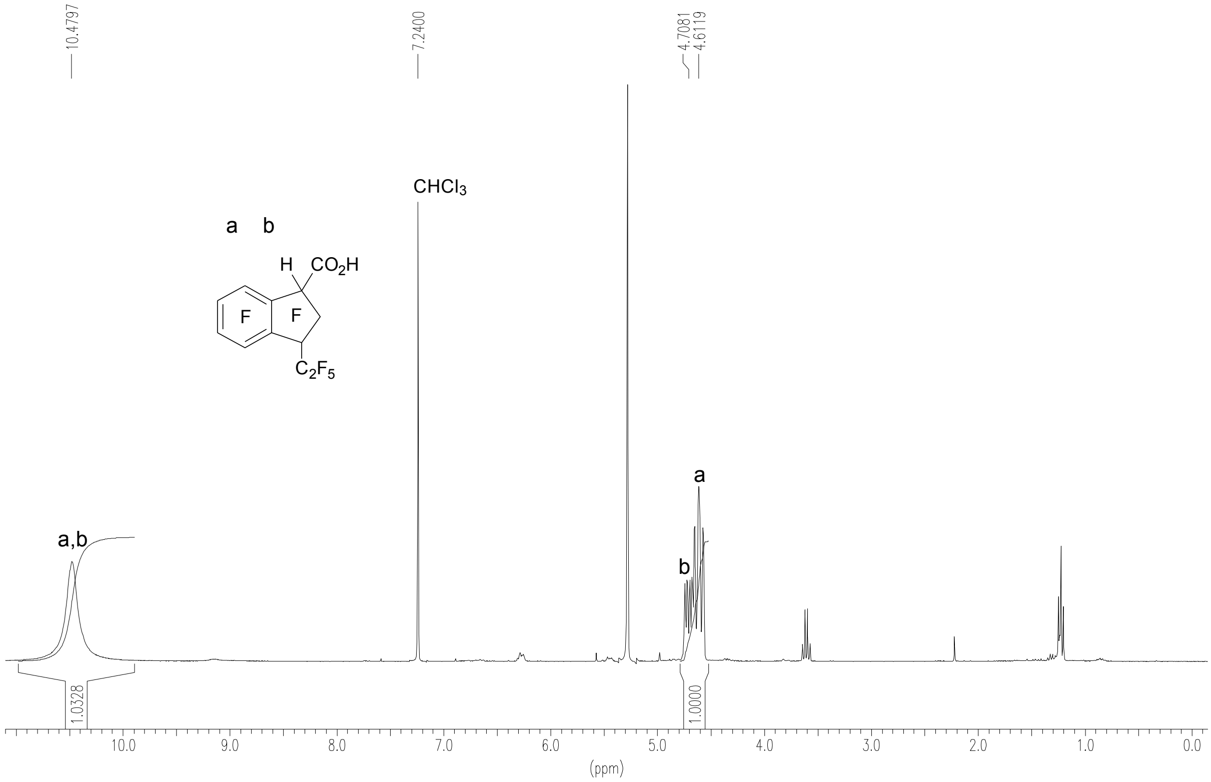

Figure S23: The ^{1}H NMR spectrum of compound **2k** ($CDCl_3$).

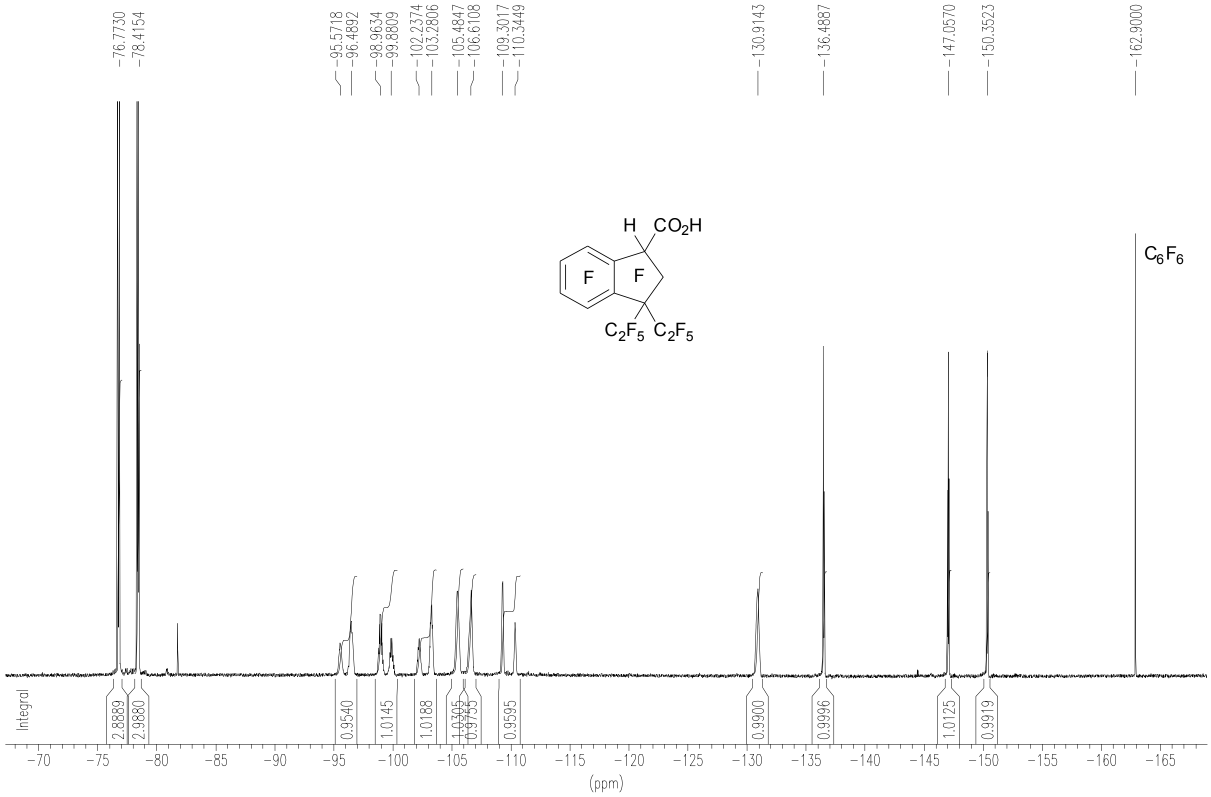

Figure S24: The ^{19}F NMR spectrum of compound **2l** ($CDCl_3$).

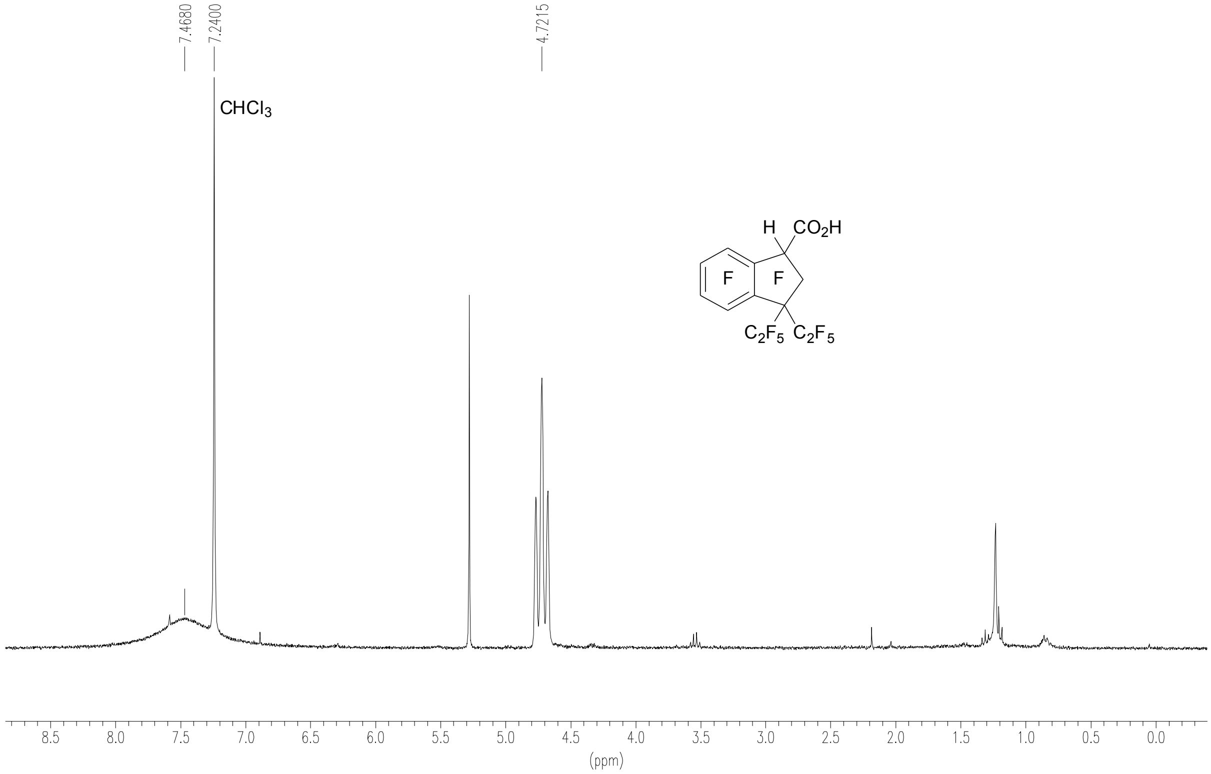

Figure S25: The ^{1}H NMR spectrum of compound **2l** ($CDCl_3$).

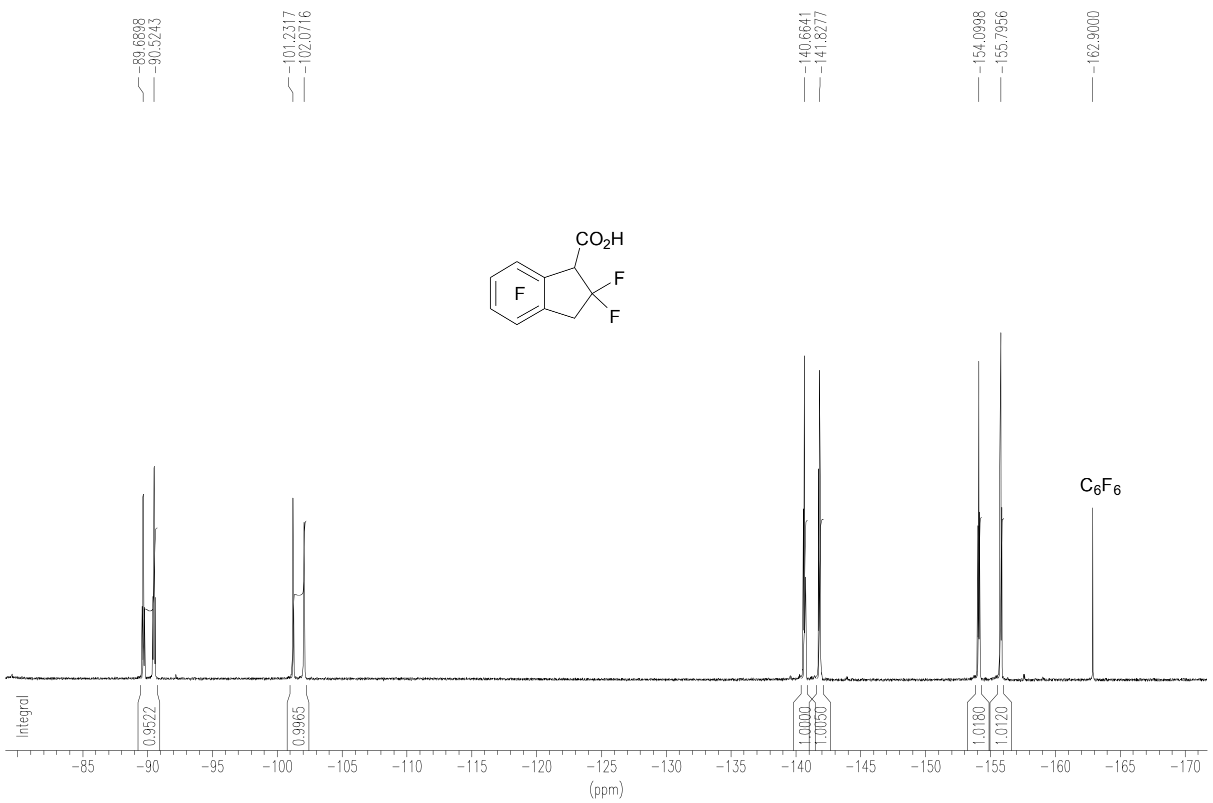

Figure S26: The ^{19}F NMR spectrum of compound **2m** ($CDCl_3$).

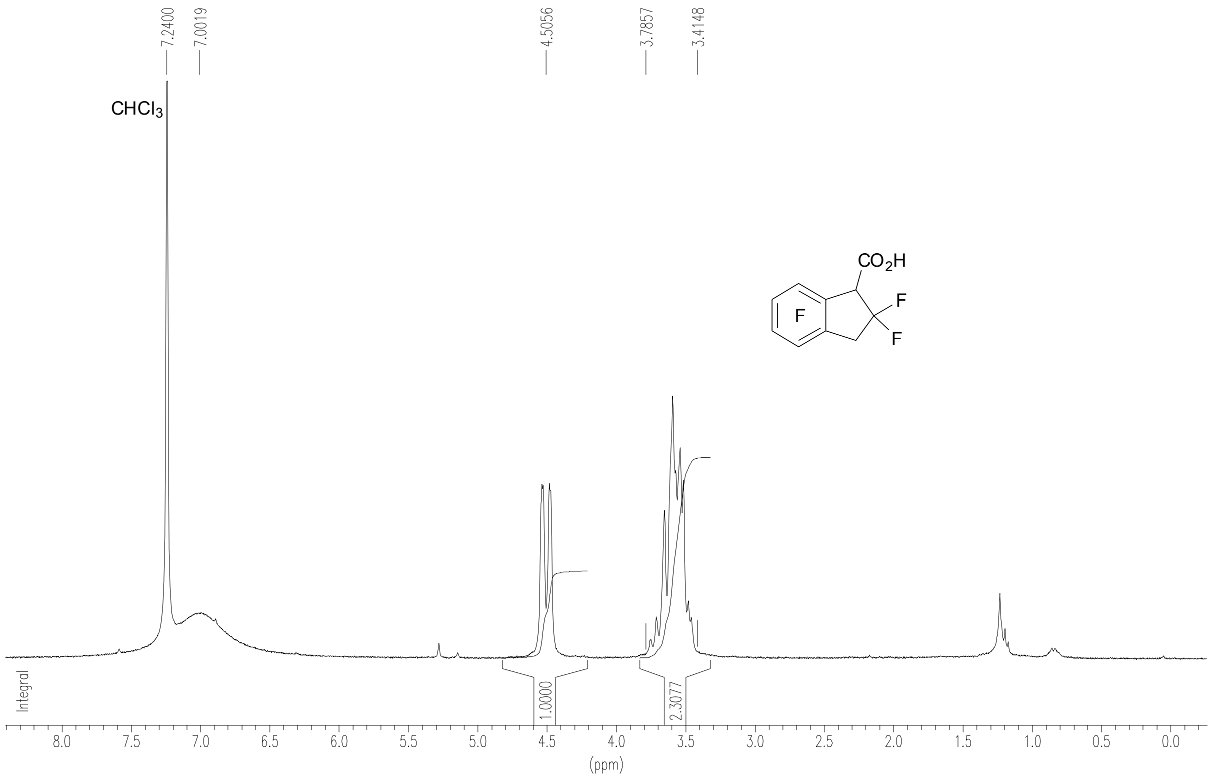

Figure S27: The ^{1}H NMR spectrum of compound **2m** ($CDCl_3$).

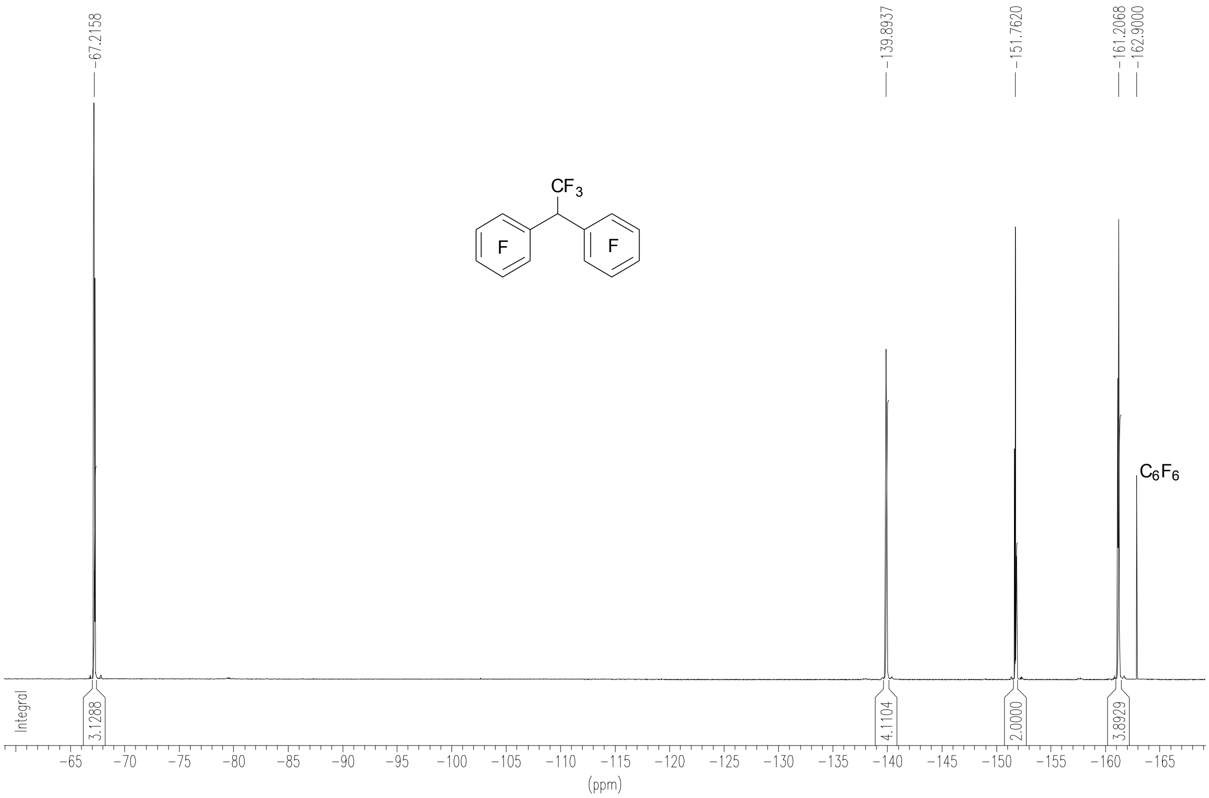

Figure S28: The ^{19}F NMR spectrum of compound **3d** ($CDCl_3$).

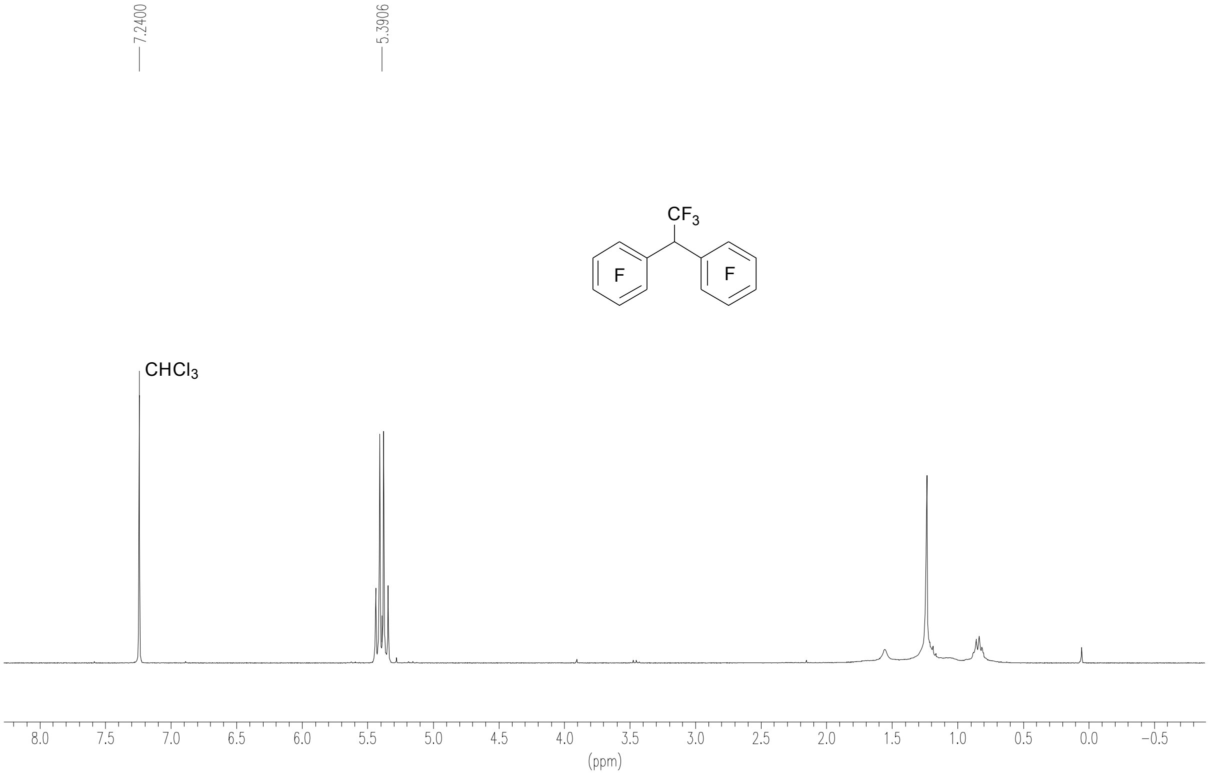

Figure S29: The ^{1}H NMR spectrum of compound **3d** ($CDCl_3$).

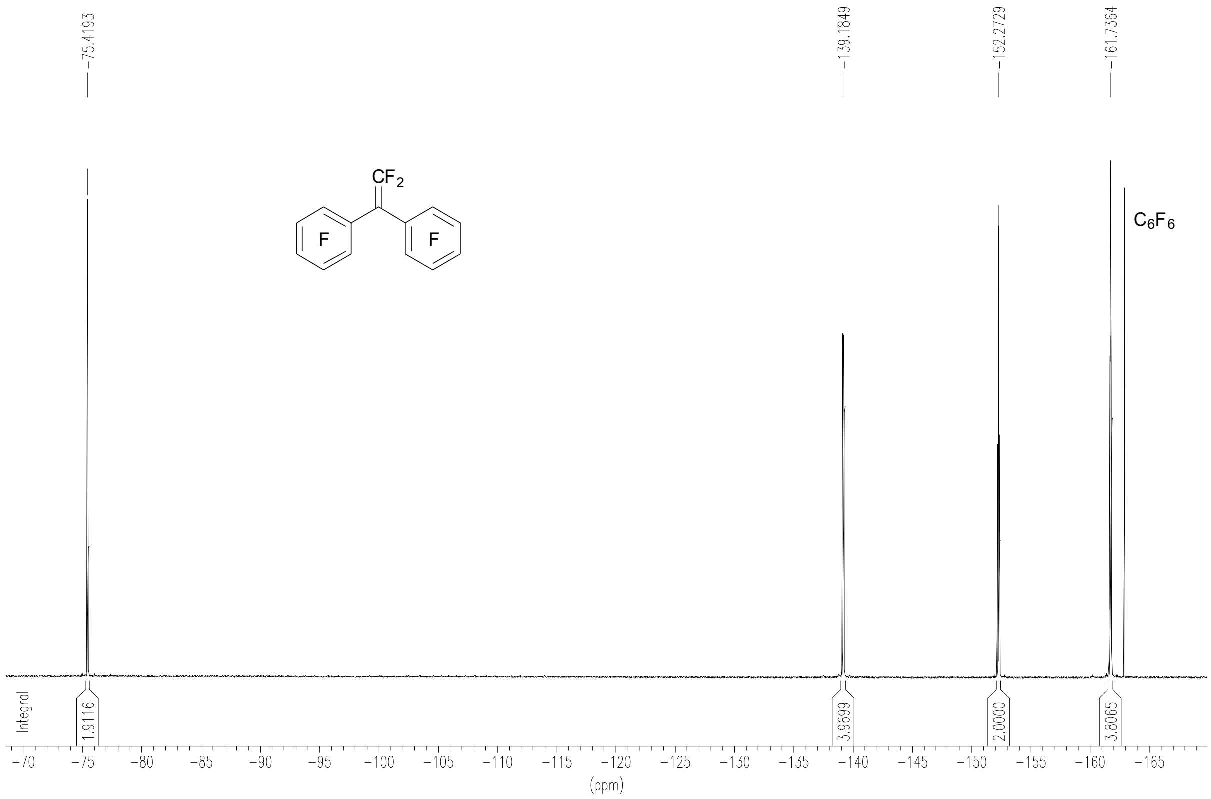

Figure S30: The ^{19}F NMR spectrum of compound **4d** ($(CD_3)_2CO$).

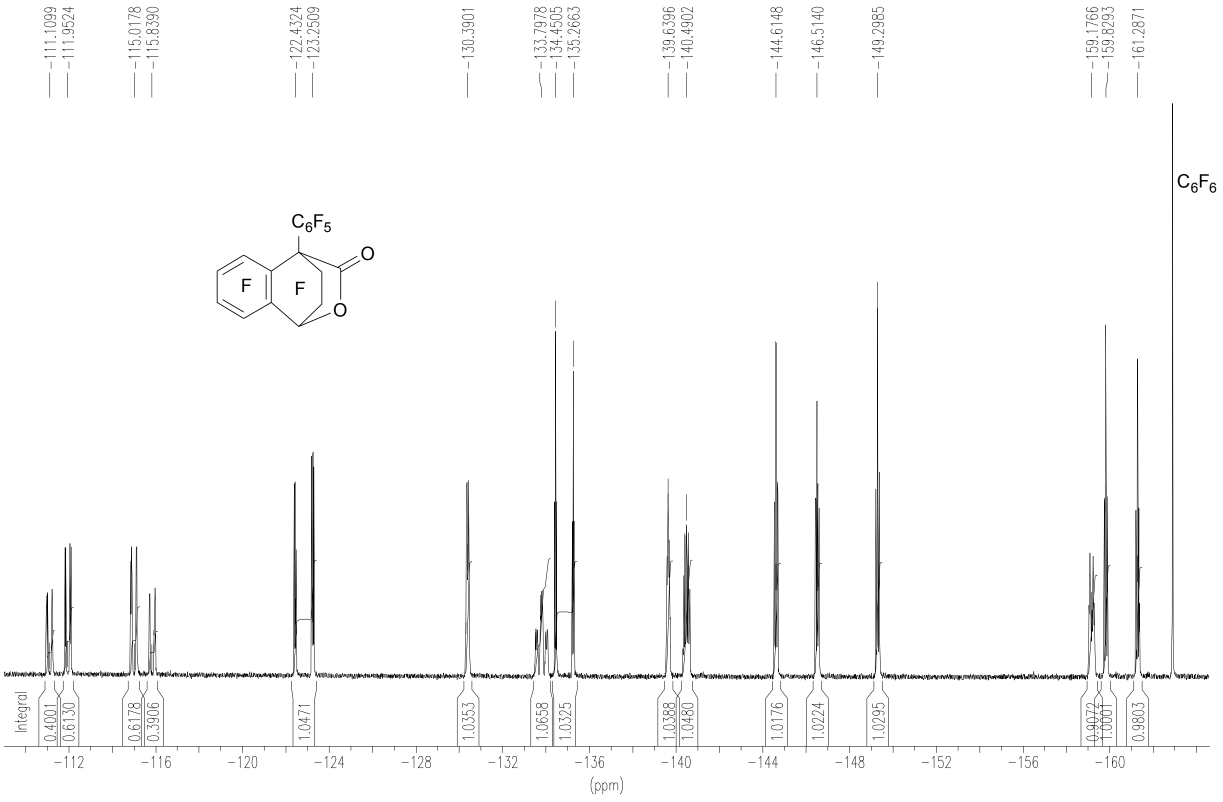

Figure S31: The ^{19}F NMR spectrum of compound **8** ($CDCl_3$).

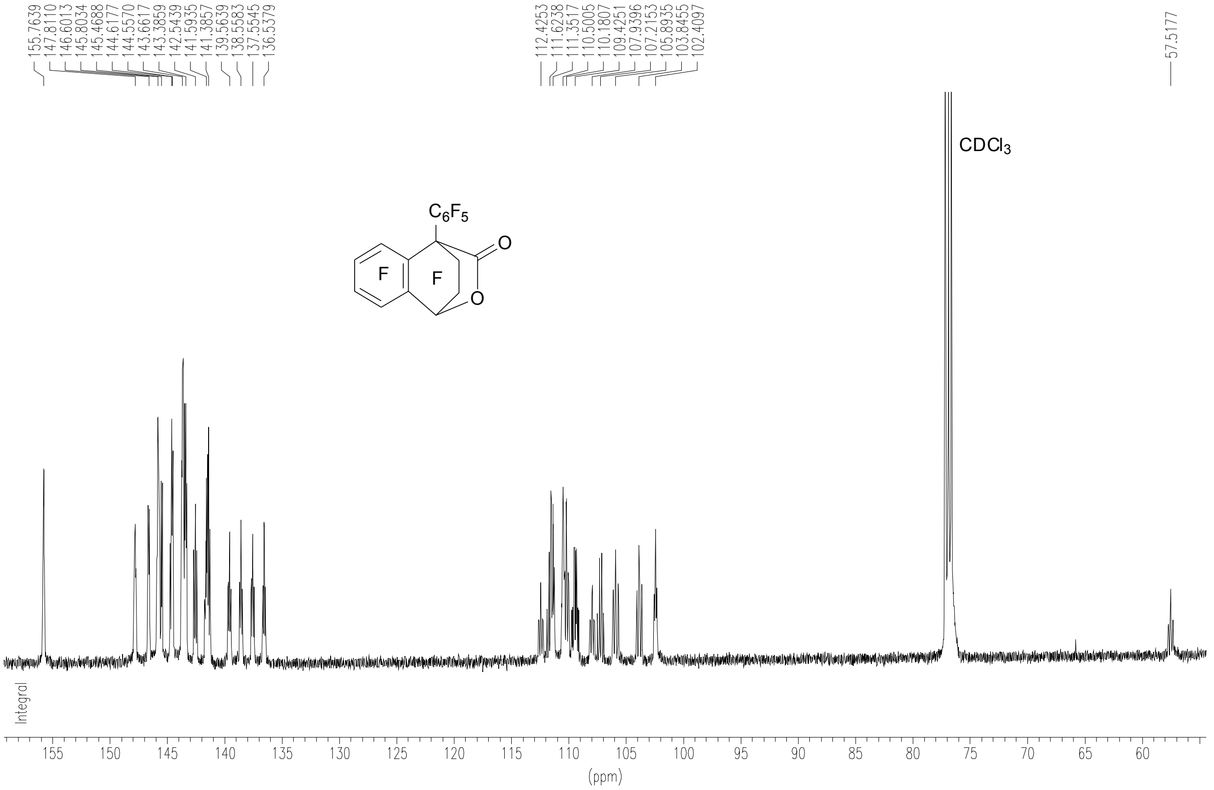

Figure S32: The ^{13}C NMR spectrum of compound **8** ($CDCl_3$).

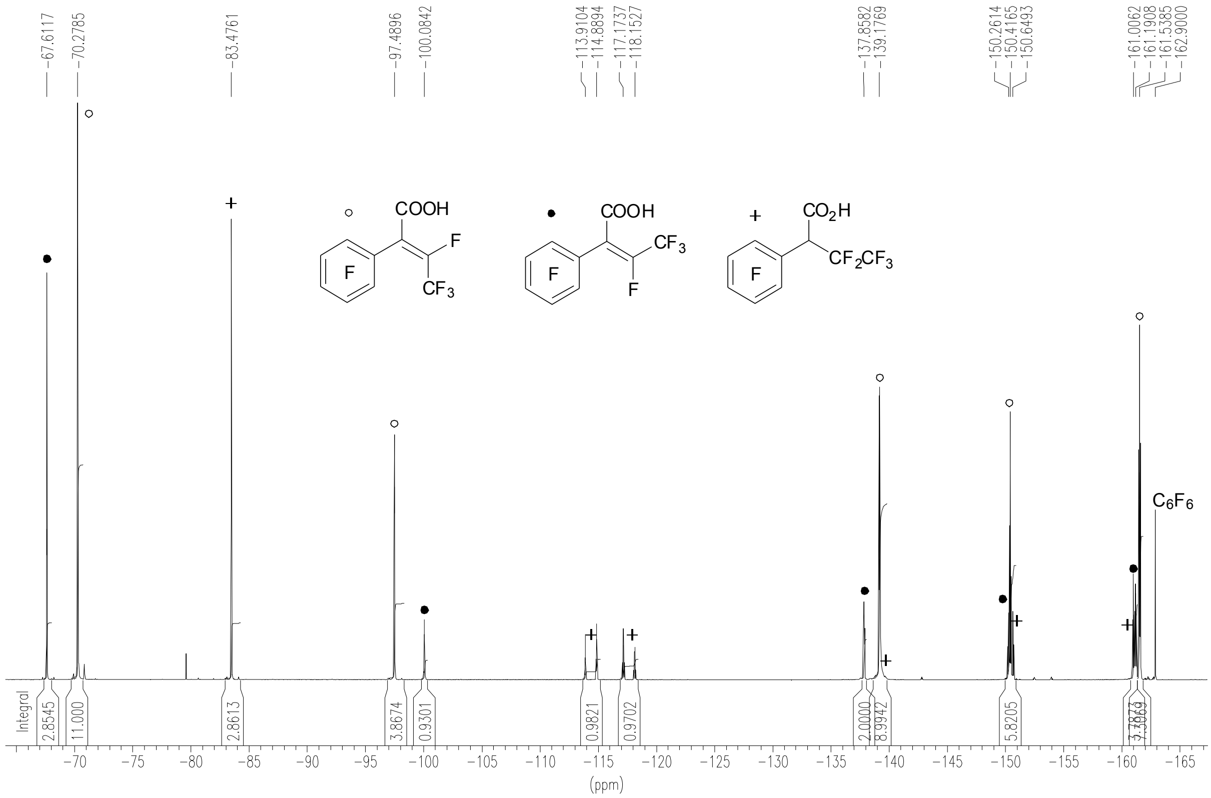

Figure S33: The ^{19}F NMR spectrum of the mixture of compounds **10g** and **2g** ($CDCl_3$).

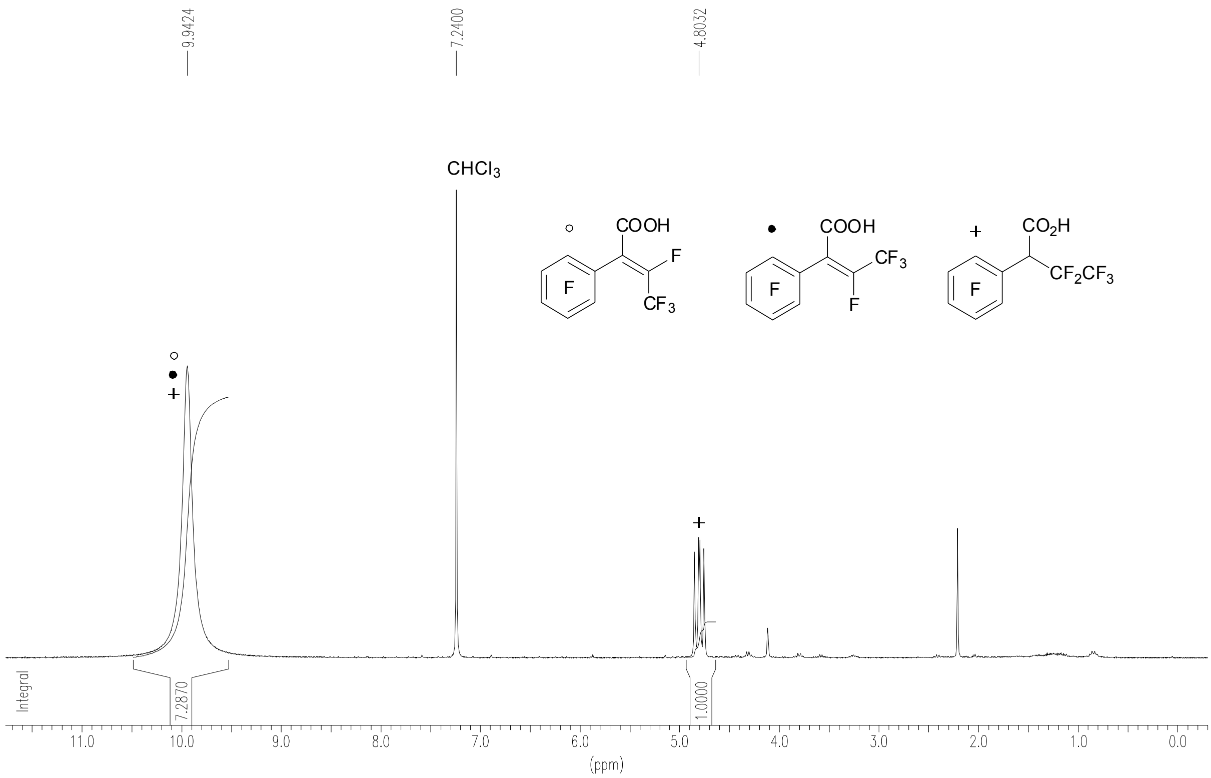

Figure S34: The ^{1}H NMR spectrum of the mixture of compounds **10g** and **2g** ($CDCl_3$).

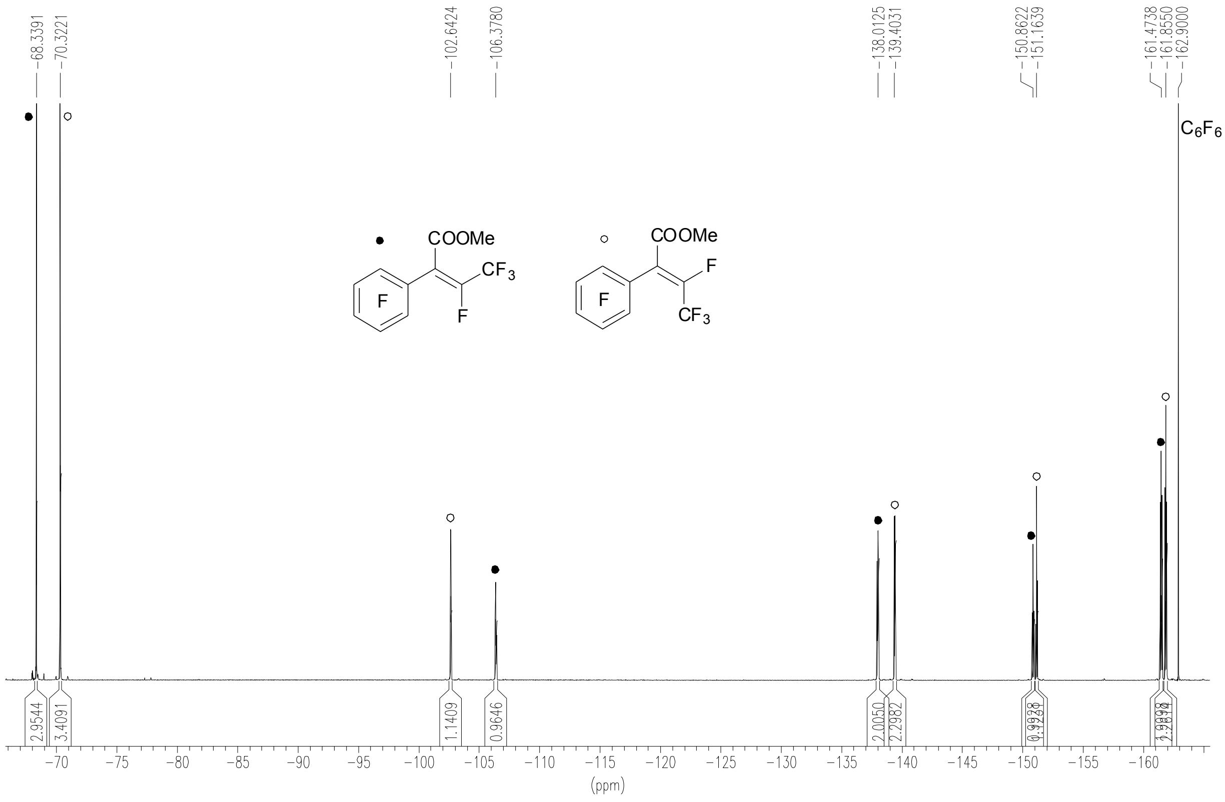

Figure S35: The ^{19}F NMR spectrum of compound **10gMe** ($CDCl_3$).

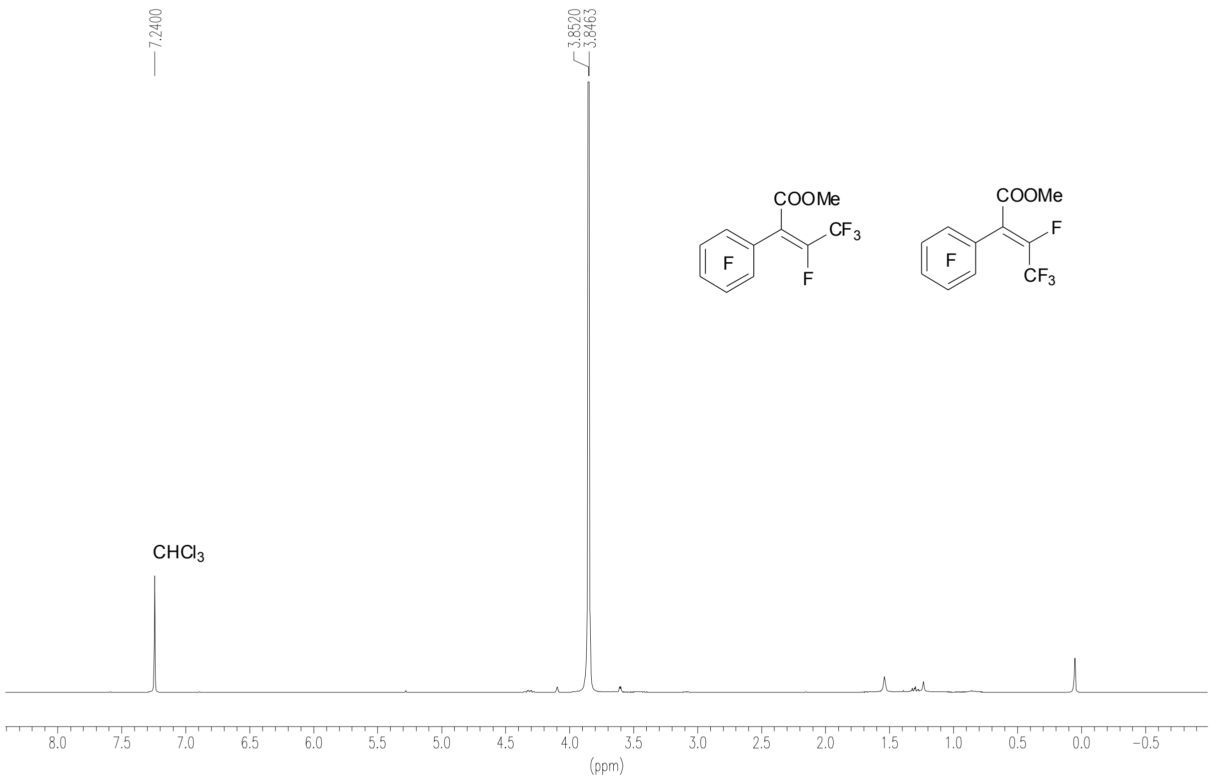

Figure S36: The ^{1}H NMR spectrum of compound **10gMe** ($CDCl_3$).

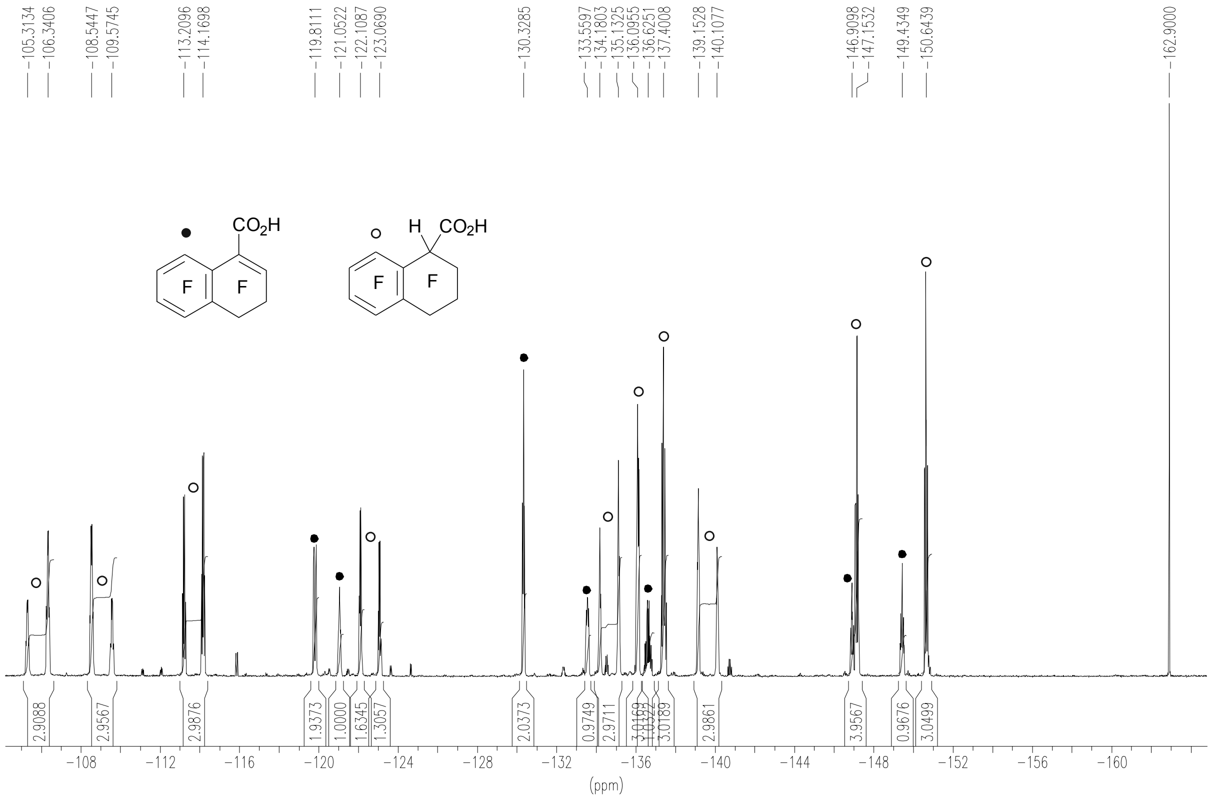

Figure S37: The ^{19}F NMR spectrum of the mixture of compounds **10h** and **2h** ($CDCl_3$).

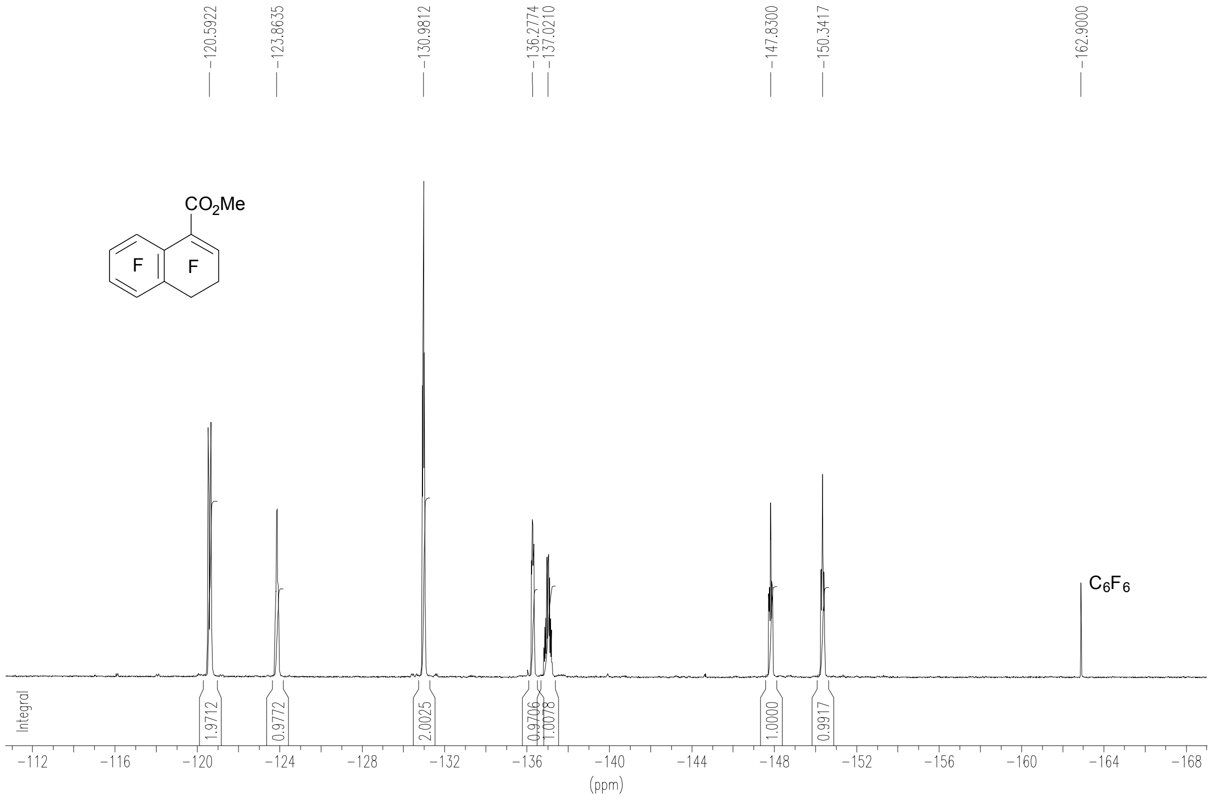

Figure S38: The ^{19}F NMR spectrum of compound **10hMe** ($CDCl_3 + CCl_4$).

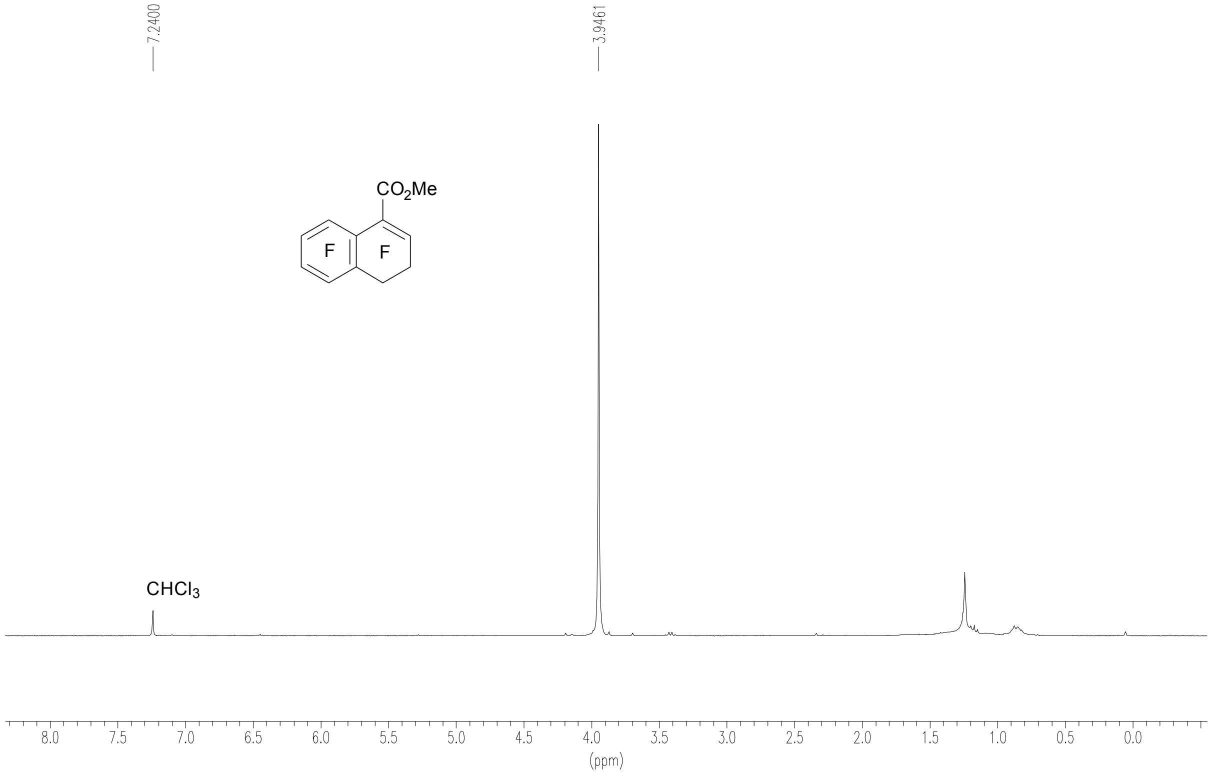

Figure S39: The ^{1}H NMR spectrum of compound **10hMe** ($CDCl_3 + CCl_4$).

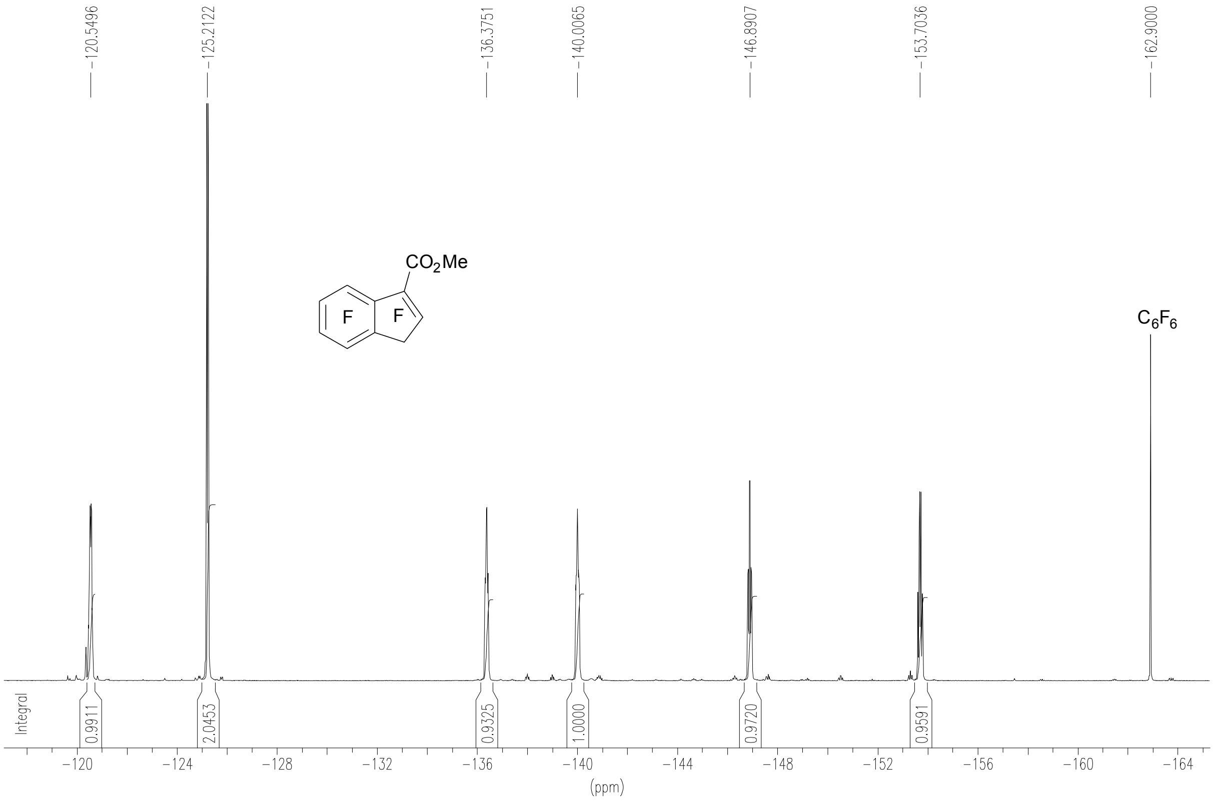

Figure S40: The ^{19}F NMR spectrum of compound **10iMe** ($CDCl_3$).

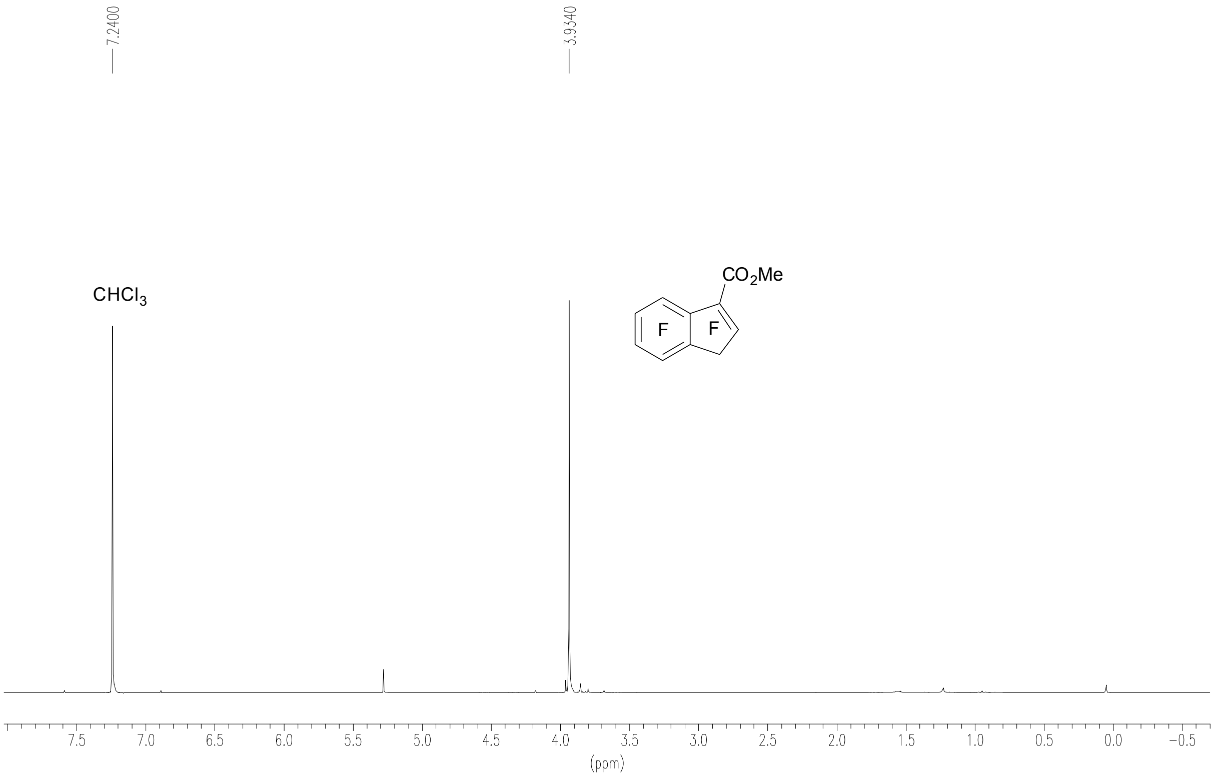

Figure S41: The ^{1}H NMR spectrum of compound **10iMe** ($CDCl_3$).

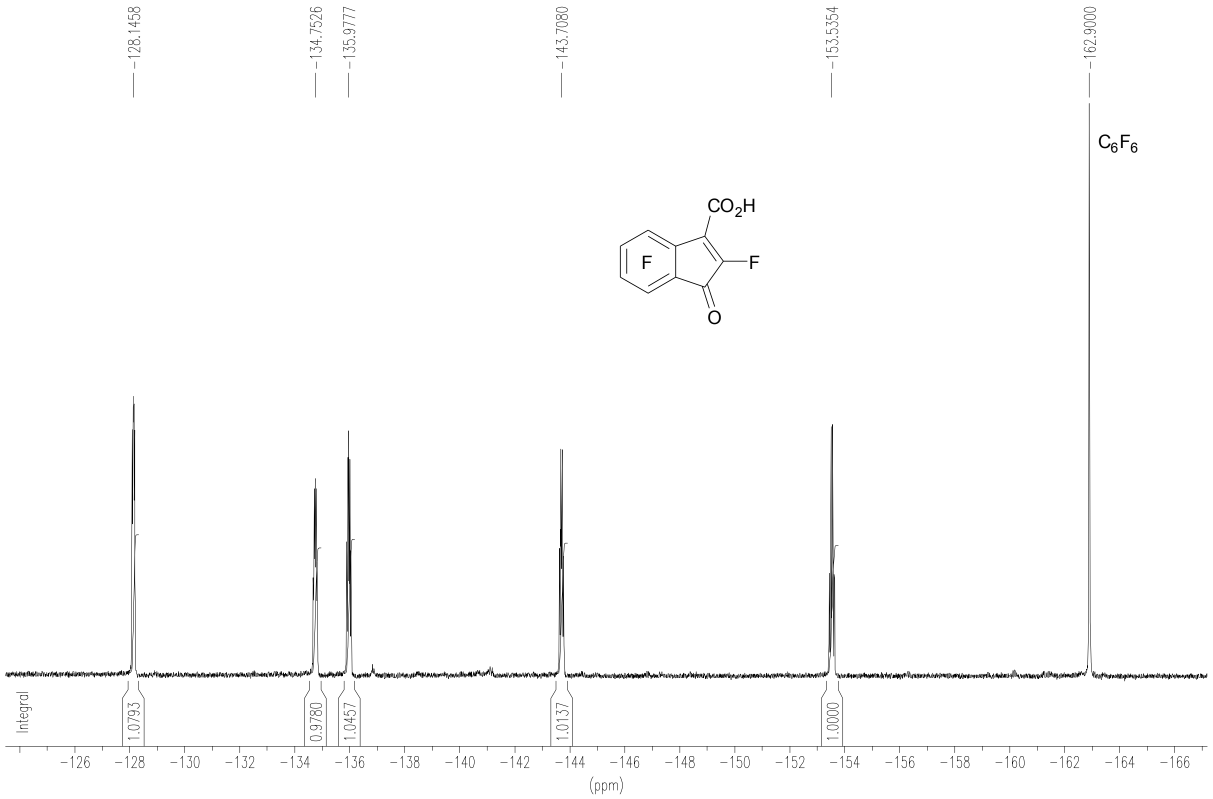

Figure S42: The ^{19}F NMR spectrum of compound **10j** ($(CD_3)_2CO$).

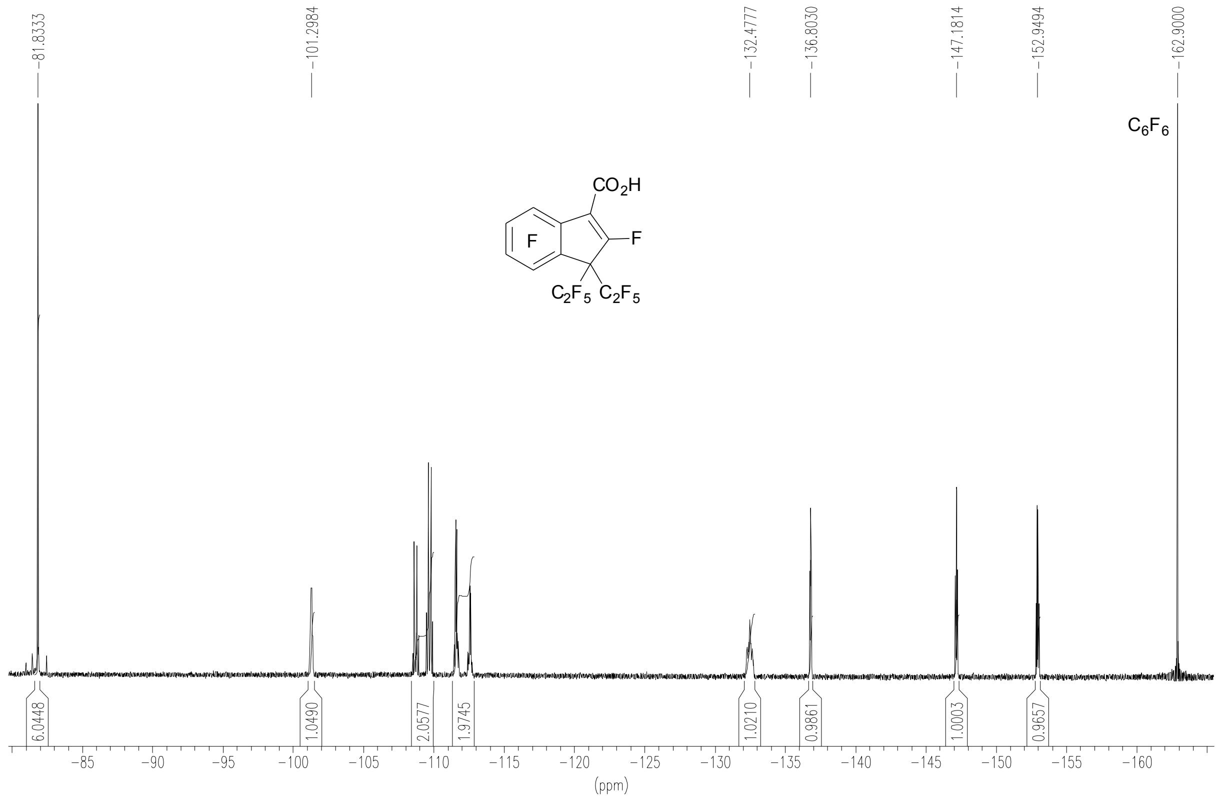

Figure S43: The ^{19}F NMR spectrum of compound **10l** ($CDCl_3$).

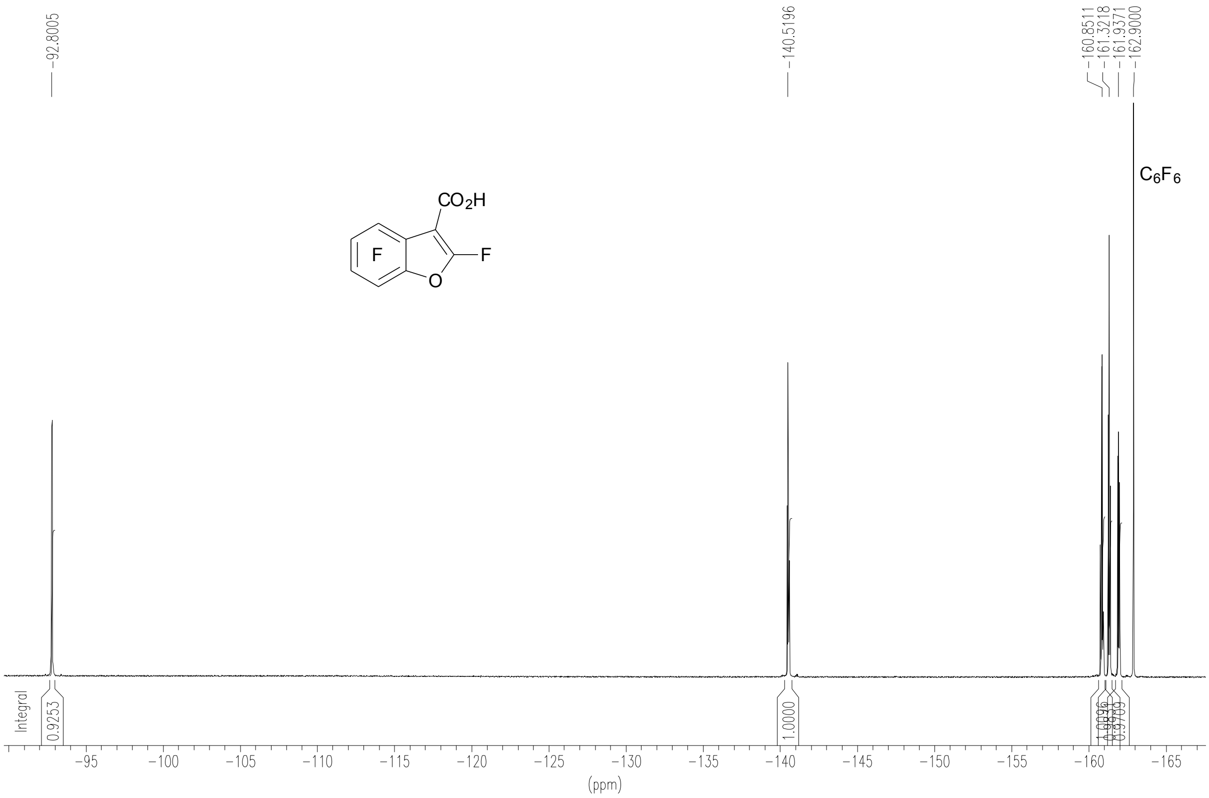

Figure S44: The ^{19}F NMR spectrum of compound **10n** ($(CD_3)_2CO$).

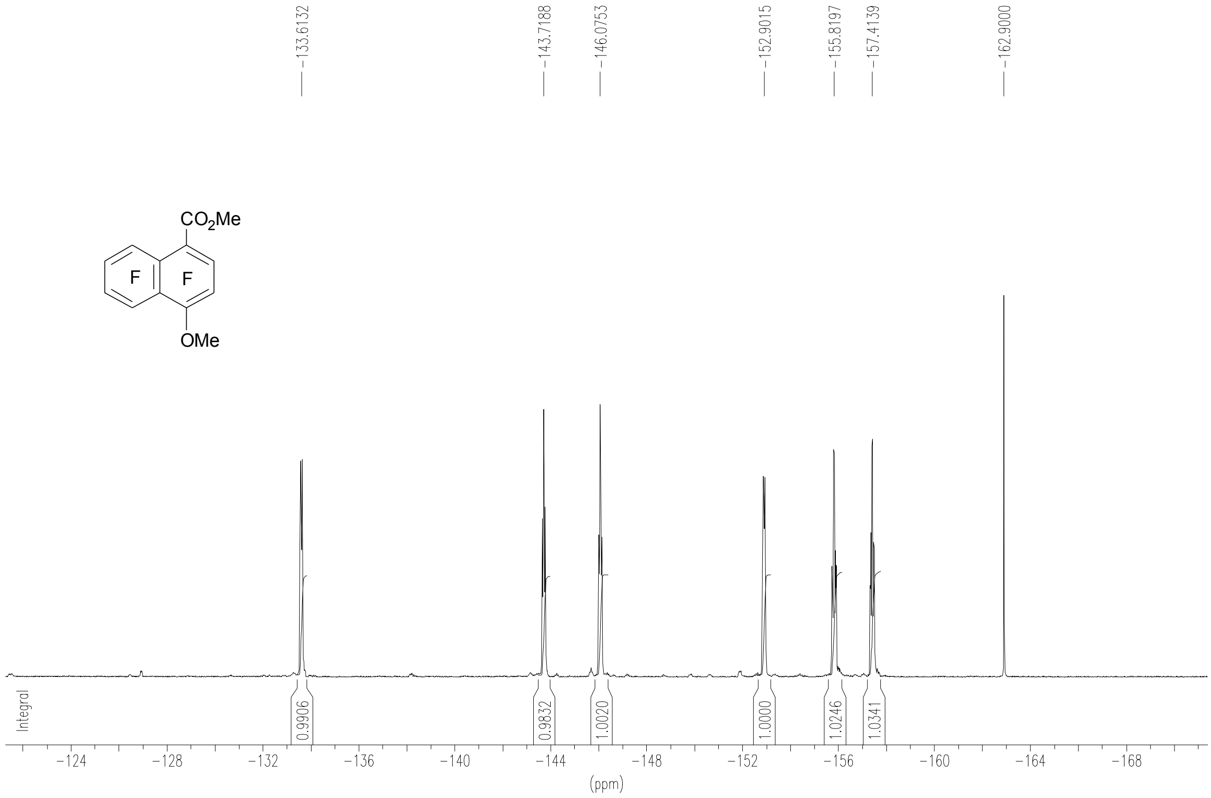

Figure S45: The ^{19}F NMR spectrum of compound **11Me** ($CDCl_3$).

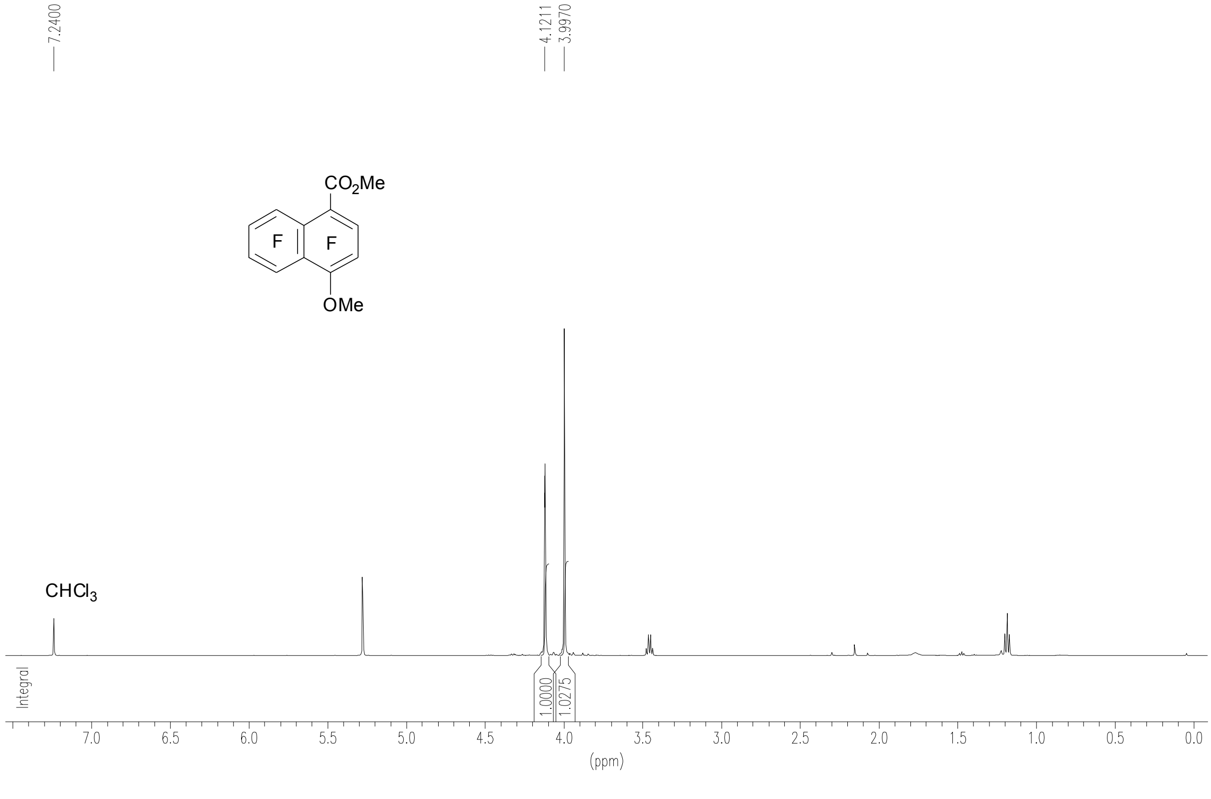

Figure S46: The ^{1}H NMR spectrum of compound **11Me** ($CDCl_3$).

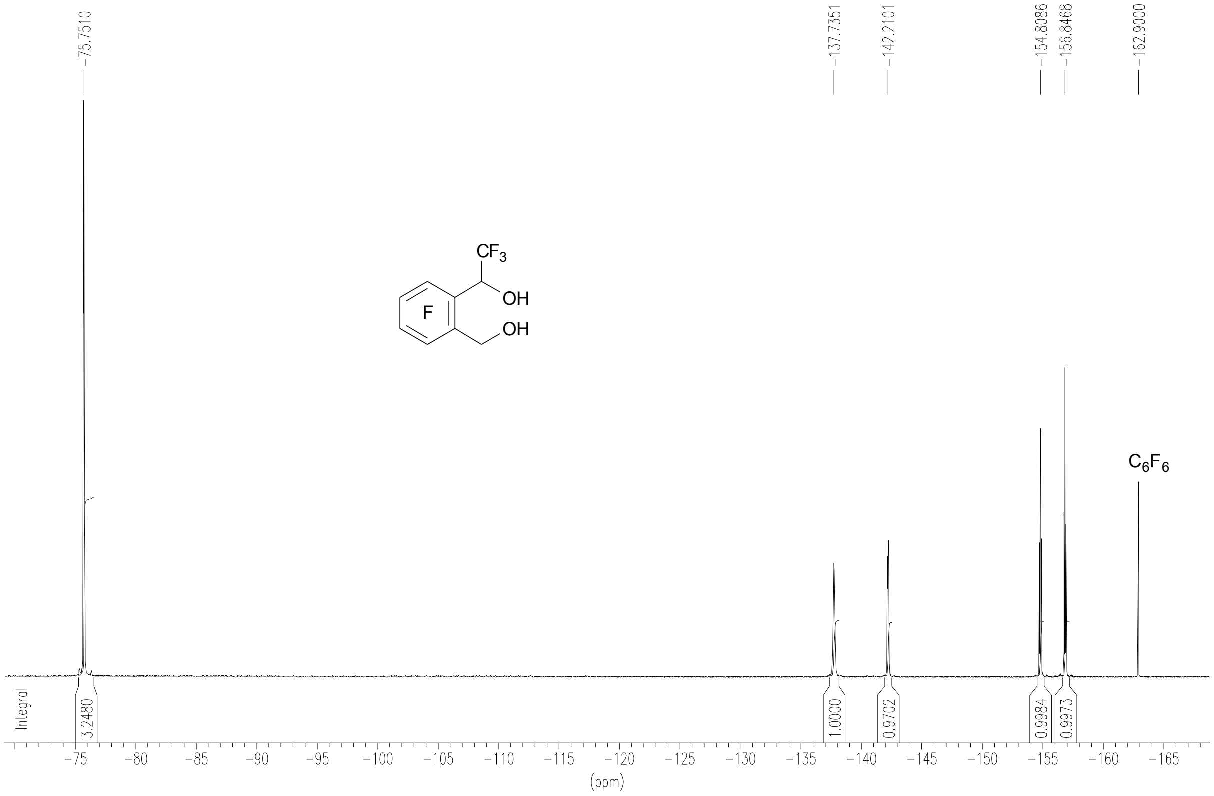

Figure S47: The ^{19}F NMR spectrum of compound **17e** ($CDCl_3$).

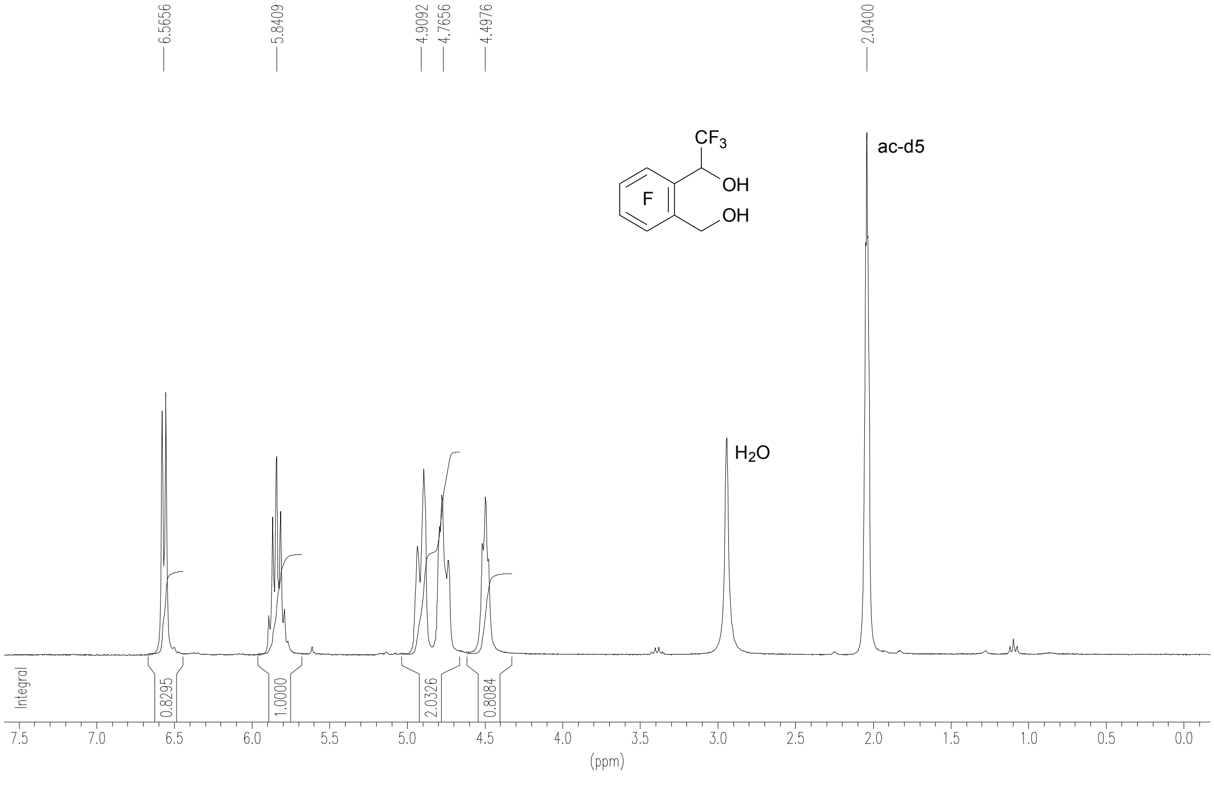

Figure S48: The ^{1}H NMR spectrum of compound **17e** ($CDCl_3$).

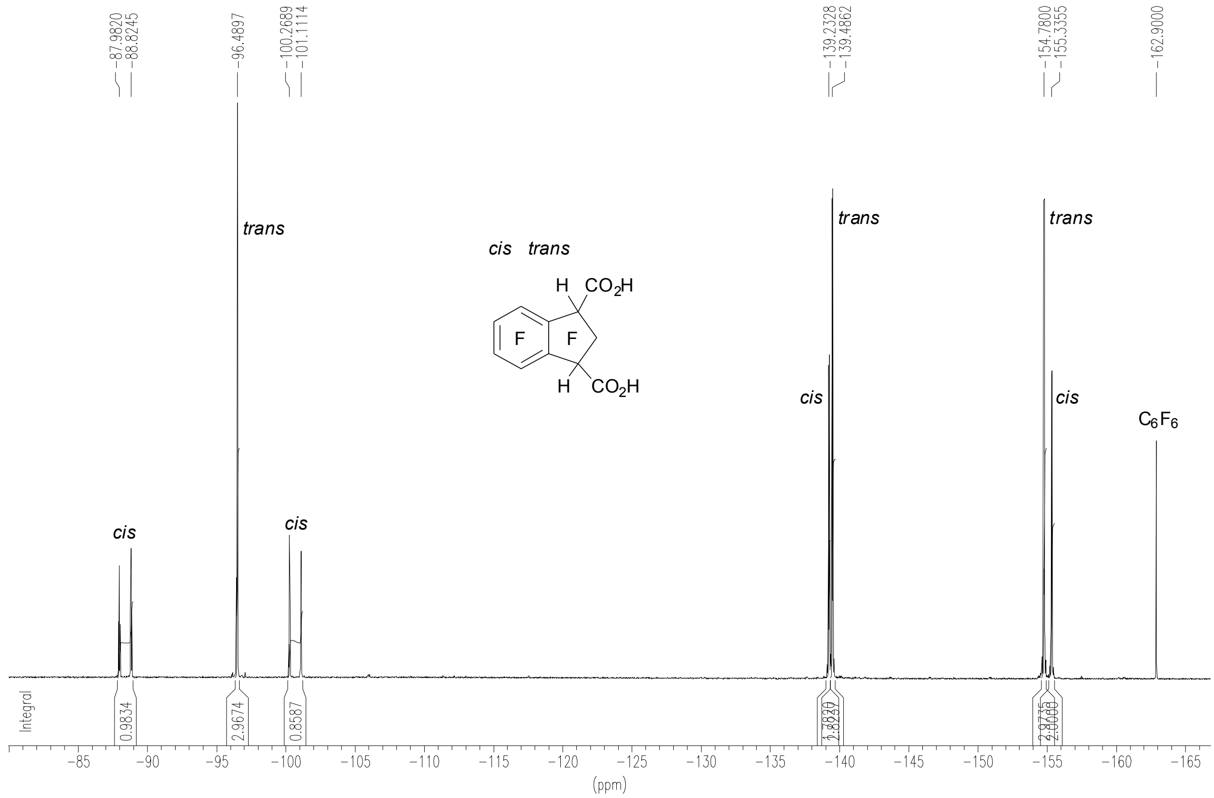

Figure S49: The ^{19}F NMR spectrum of compound **18a** ($(CD_3)_2CO$).

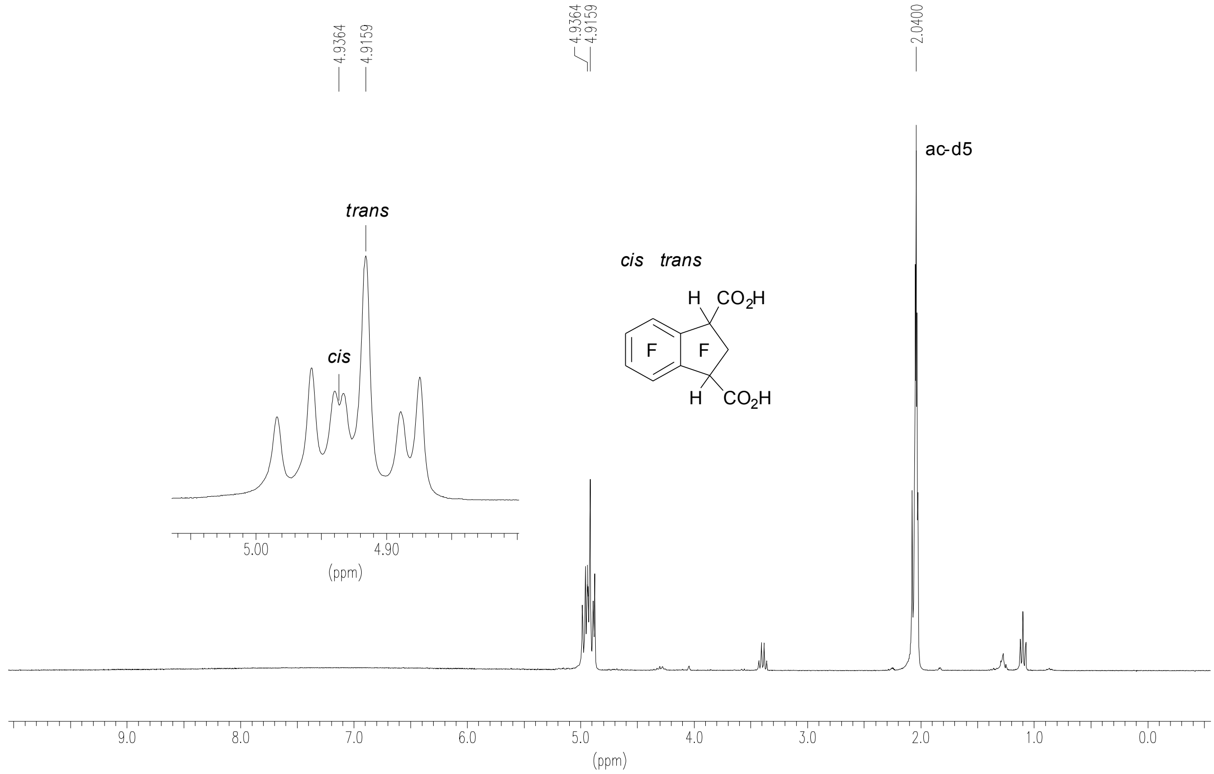

Figure S50: The ^{1}H NMR spectrum of compound **18a** ($(CD_3)_2CO$).

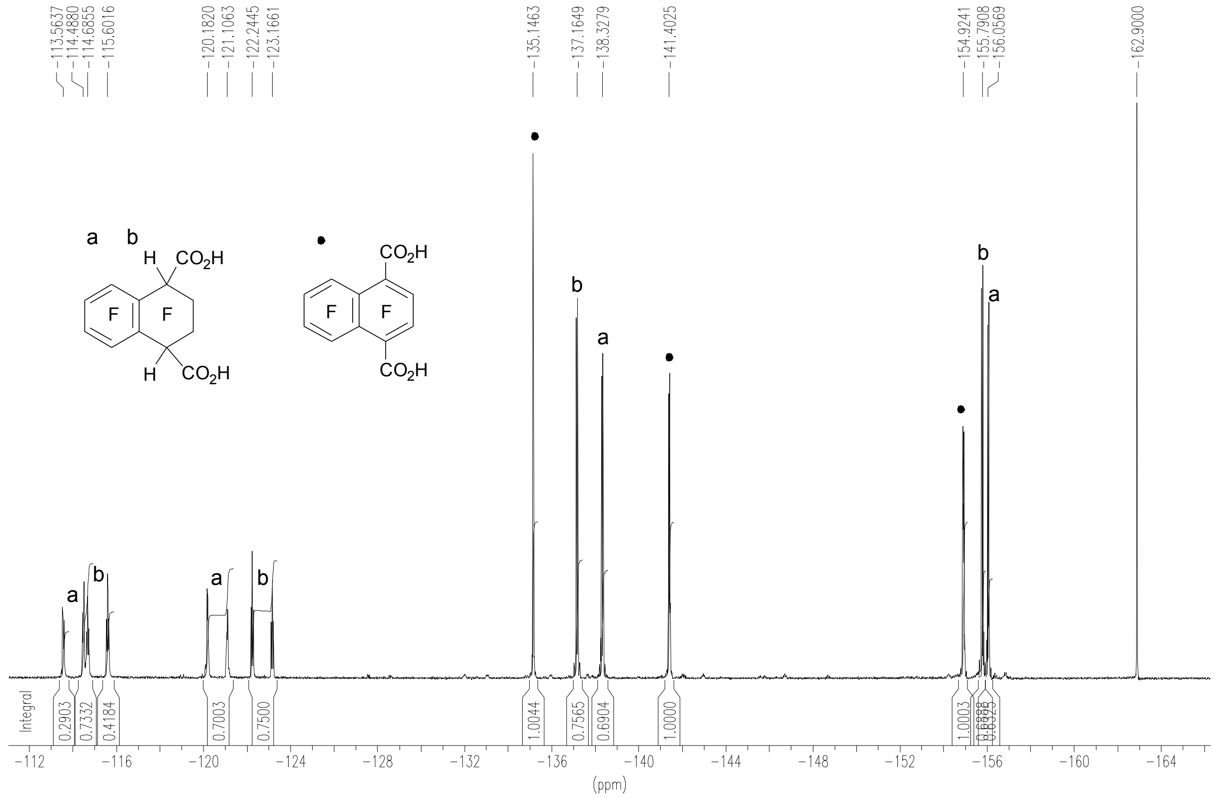

Figure S51: The ^{19}F NMR spectrum of the mixture of compounds **18b** and **19** ($(CD_3)_2CO$).

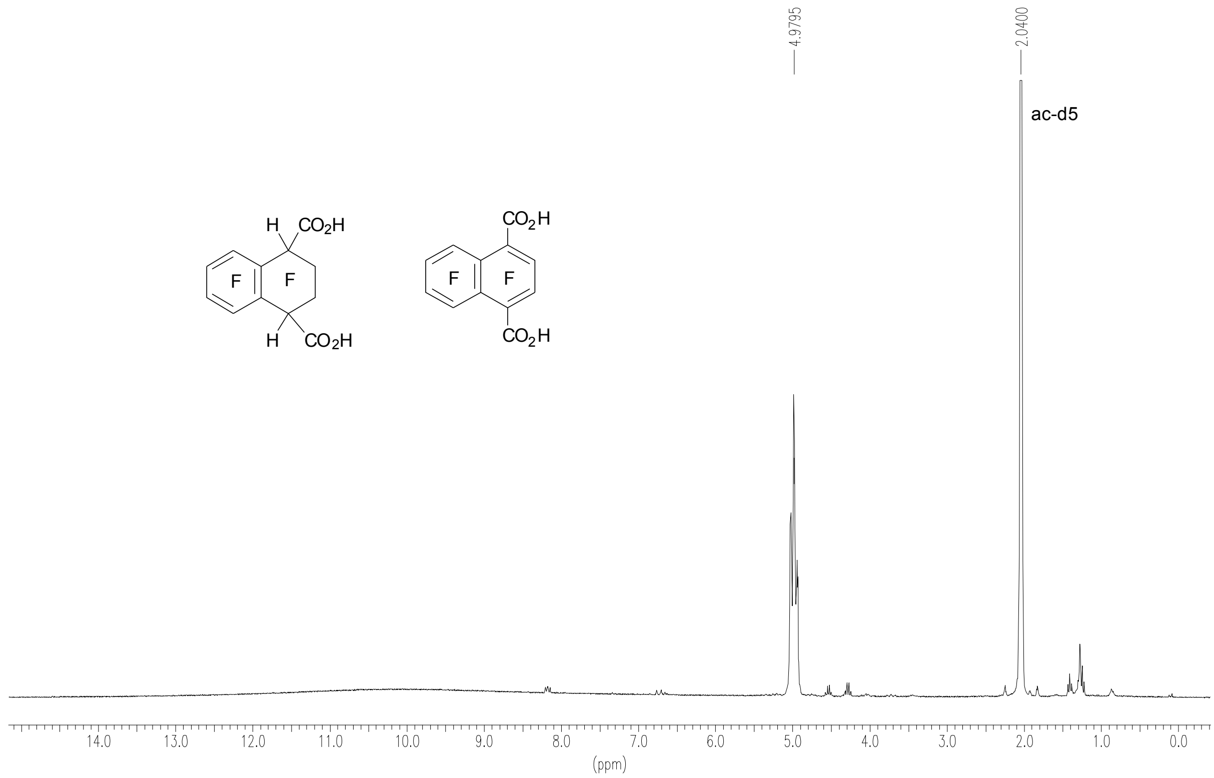

Figure S52: The ^{1}H NMR spectrum of the mixture of compounds **18b** and **19** ($(CD_3)_2CO$).

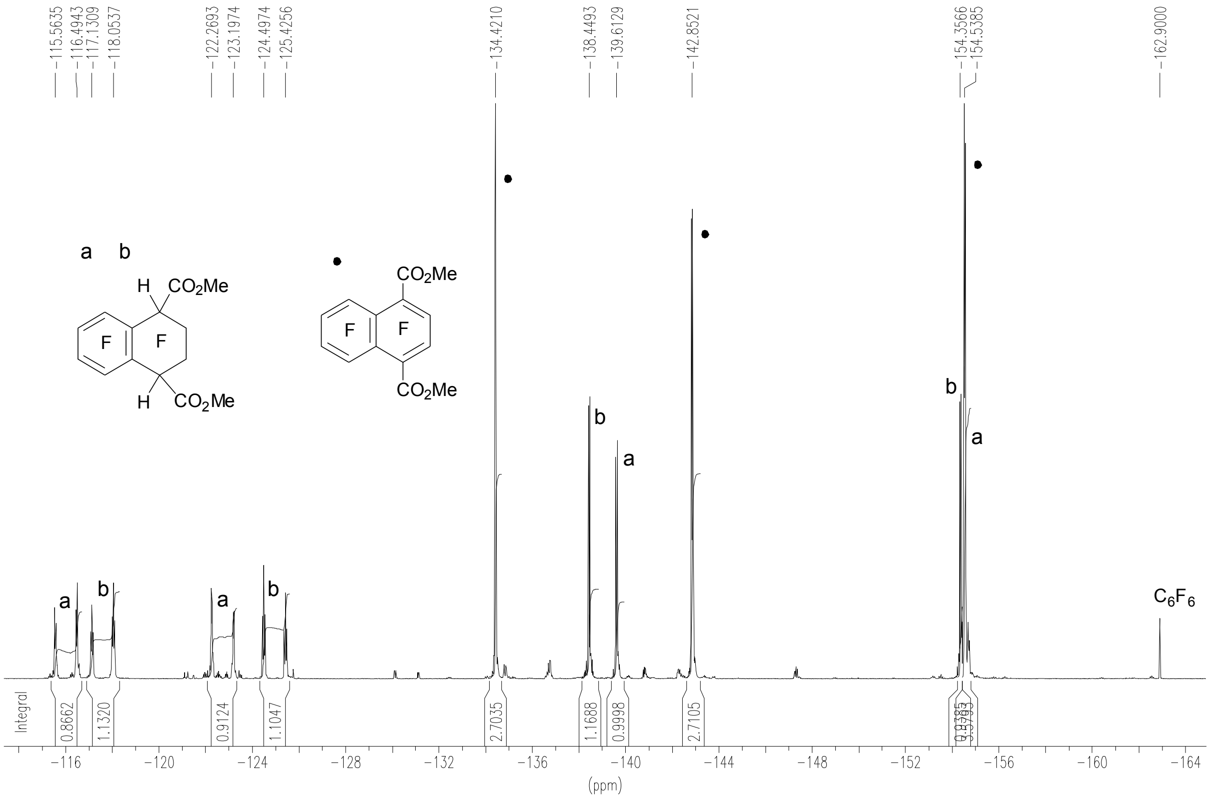

Figure S53: The ^{19}F NMR spectrum of the mixture of compounds **18bMe** and **19Me** ($CDCl_3$).

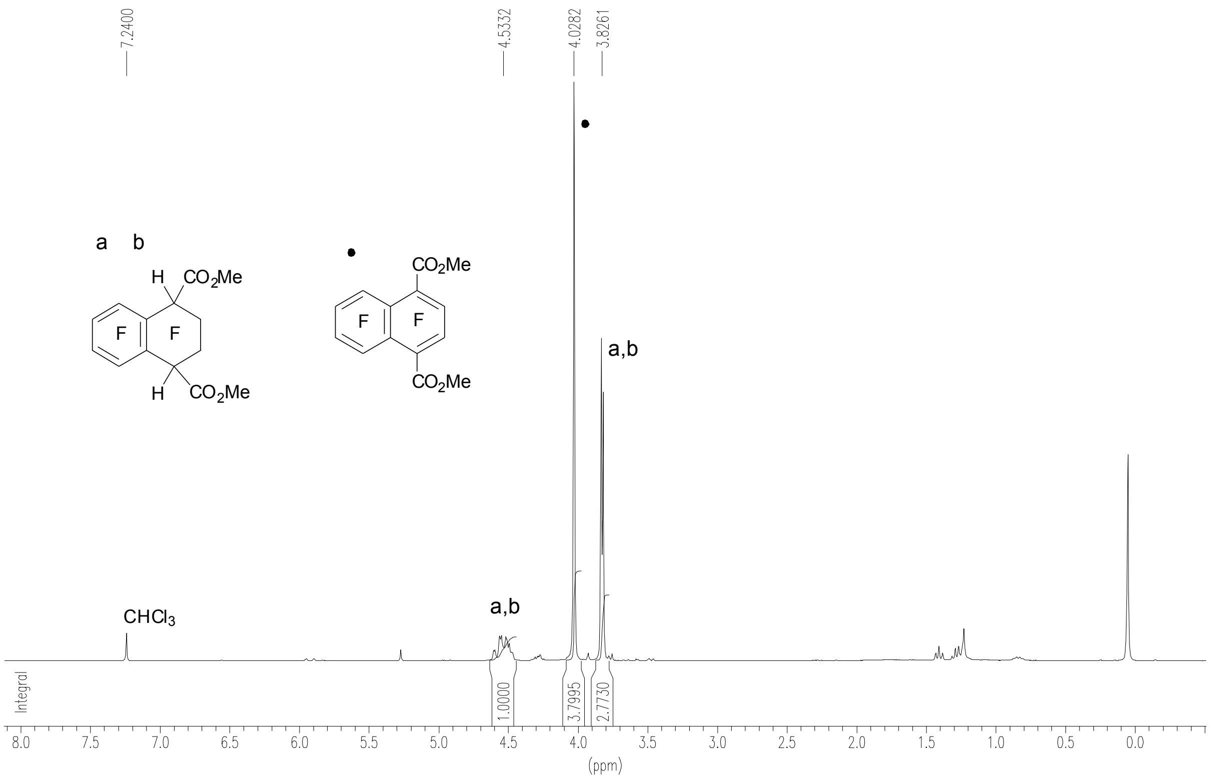

Figure S54: The ^{1}H NMR spectrum of the mixture of compounds **18bMe** and **19Me** ($CDCl_3$).

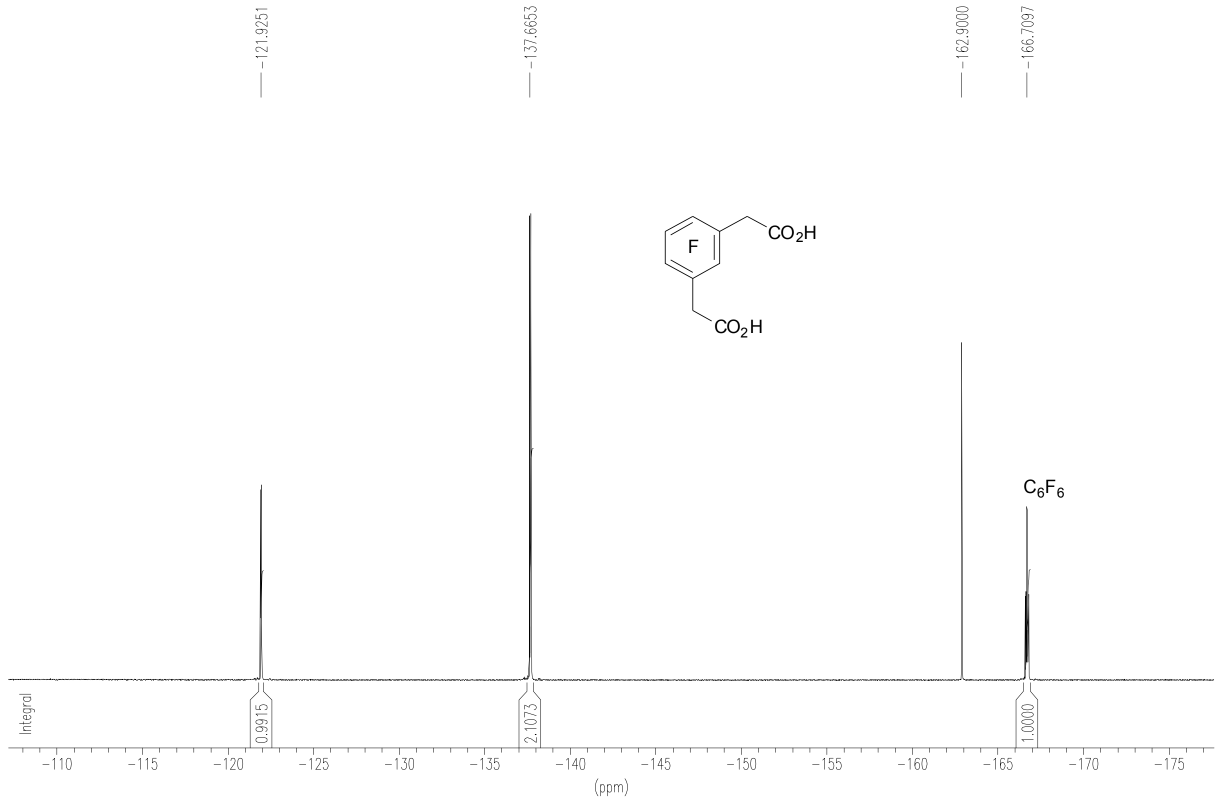

Figure S55: The ^{19}F NMR spectrum of compound **18f** ($(CD_3)_2CO$).

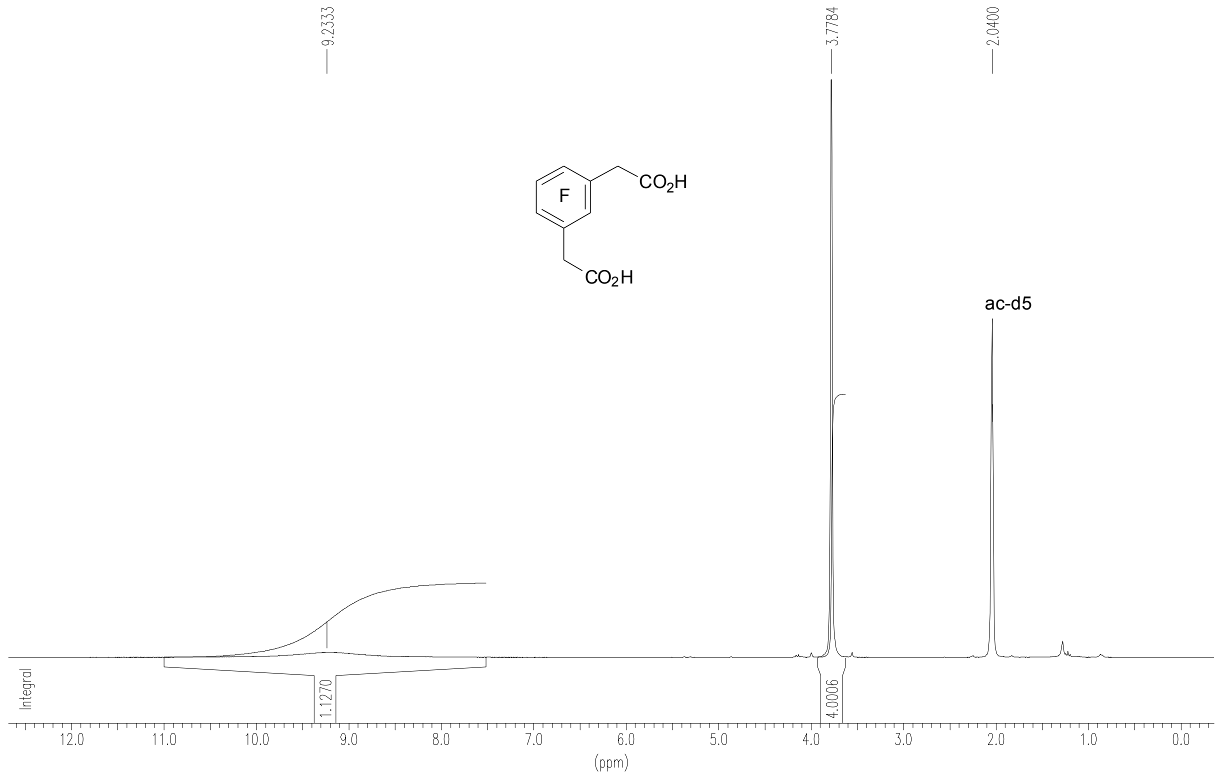

Figure S56: The ^{1}H NMR spectrum of compound **18f** ($(CD_3)_2CO$).

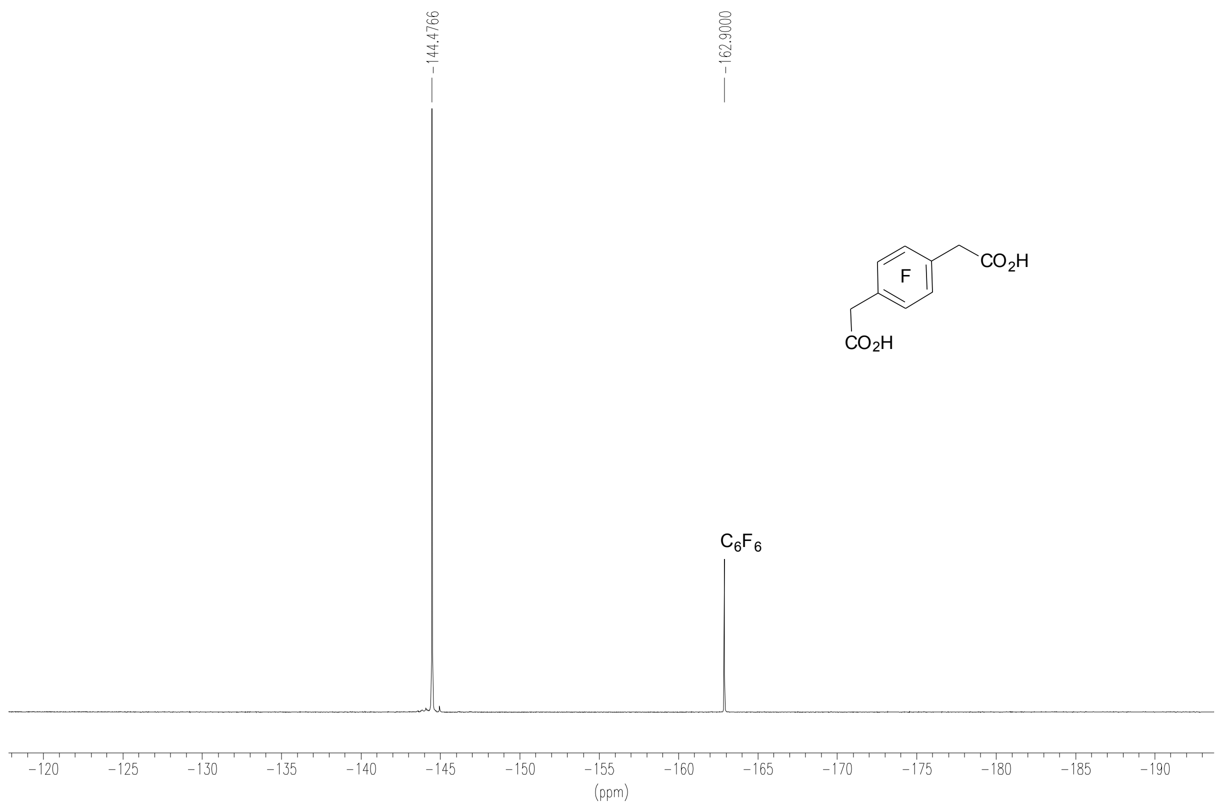

Figure S57: The ^{19}F NMR spectrum of compound **18g** ($(CD_3)_2SO$).

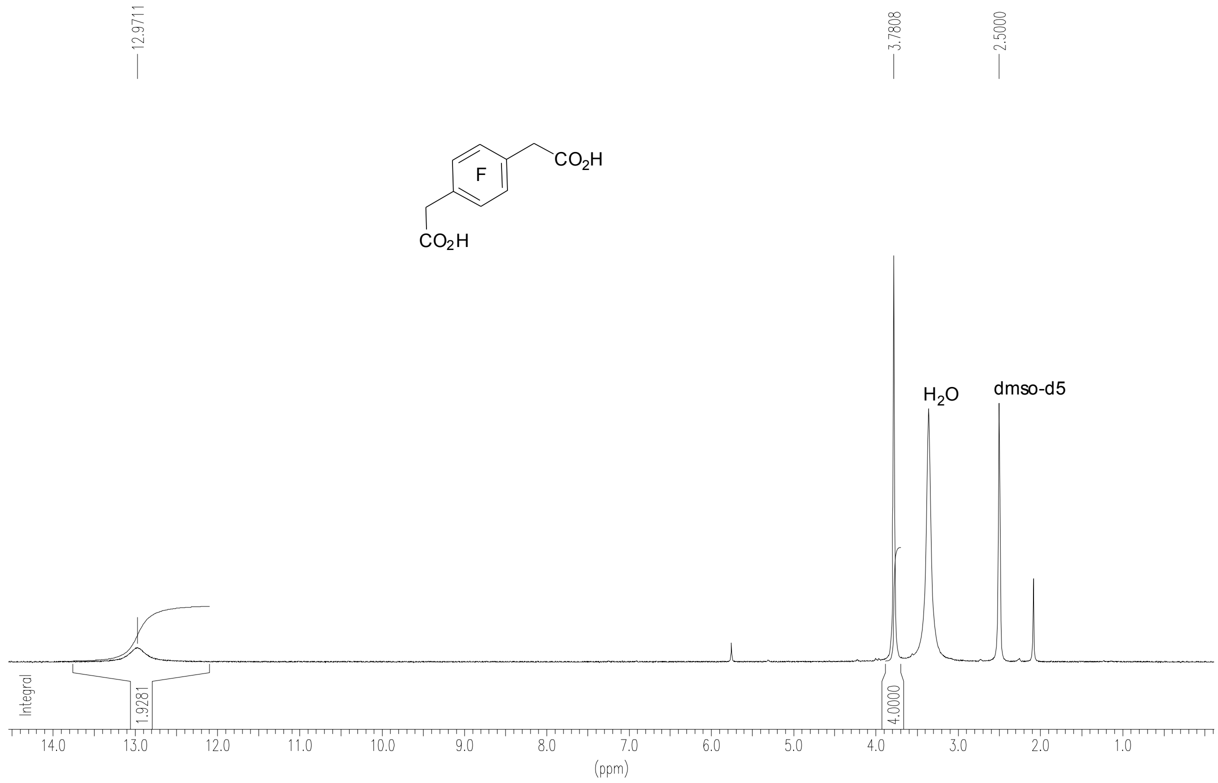

Figure S58: The ^{1}H NMR spectrum of compound **18g** ($(CD_3)_2SO$).

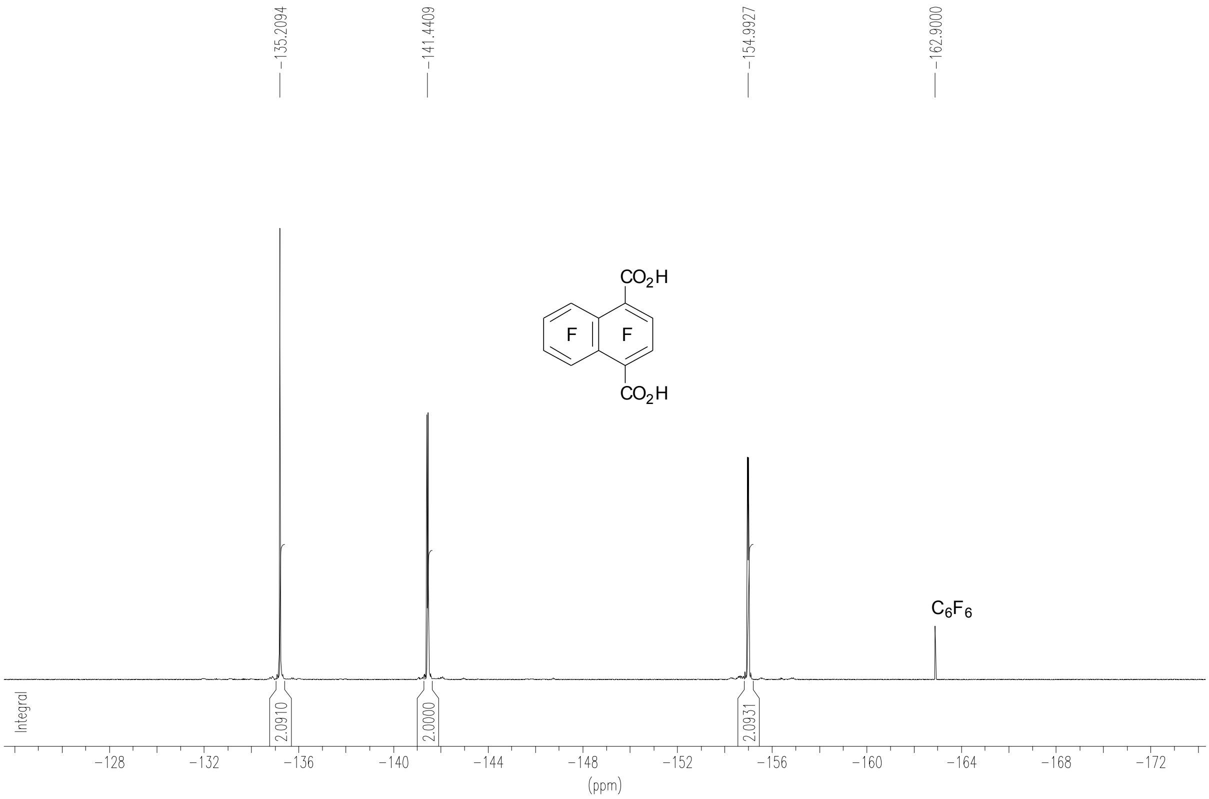

Figure S59: The ^{19}F NMR spectrum of compound **19** ($(CD_3)_2CO$).

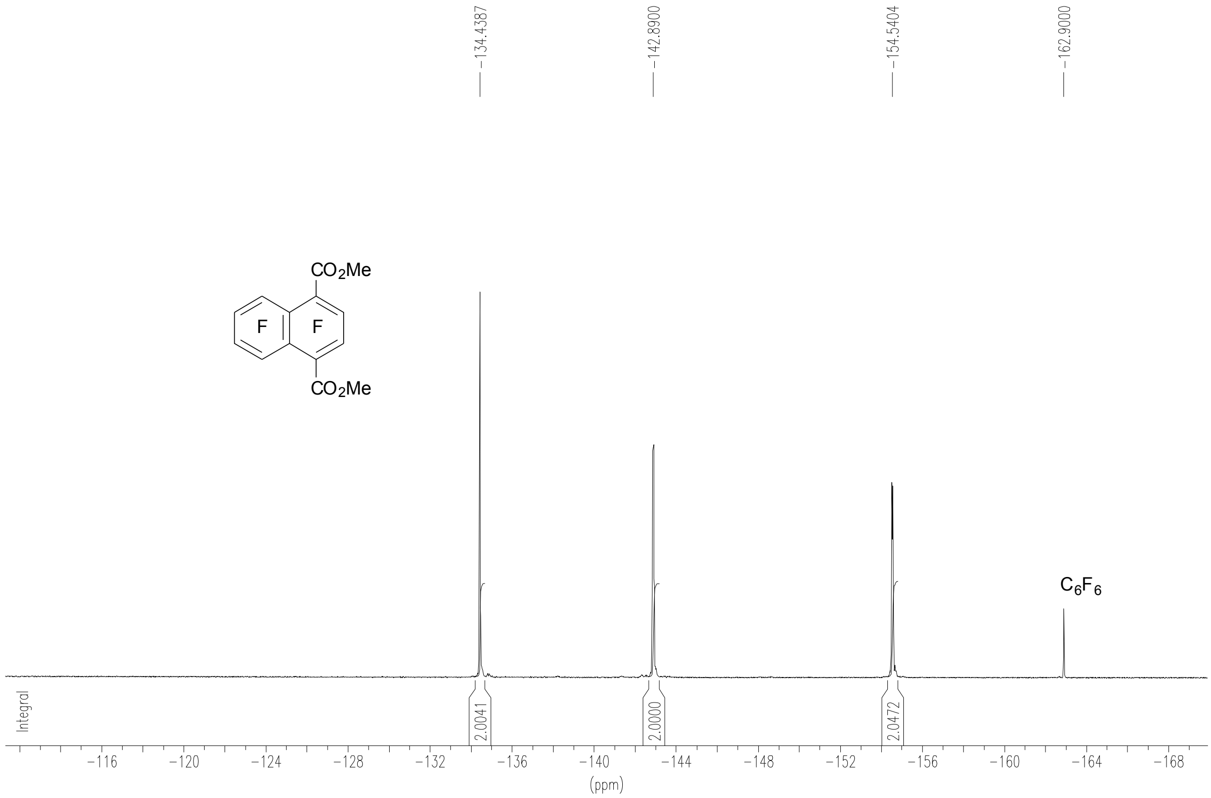

Figure S60: The ^{19}F NMR spectrum of compound **19Me** ($CDCl_3$).

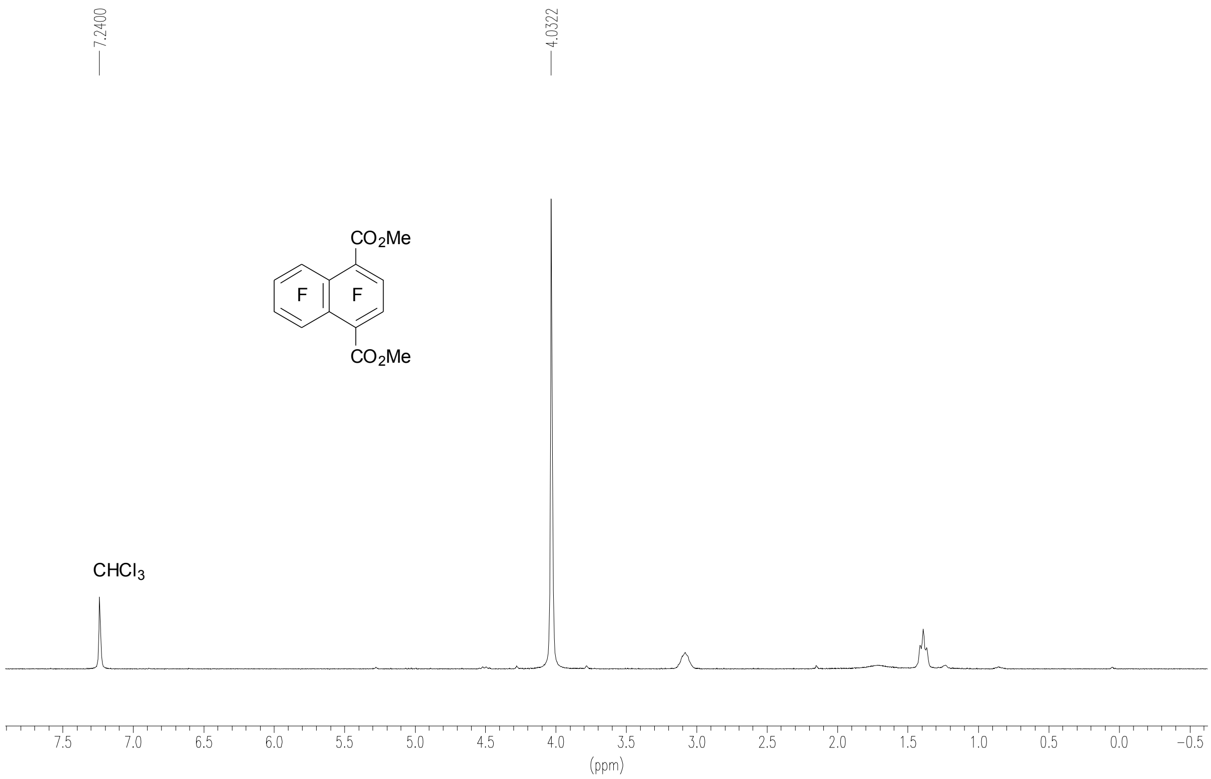

Figure S61: The ^{1}H NMR spectrum of compound **19Me** ($CDCl_3$).

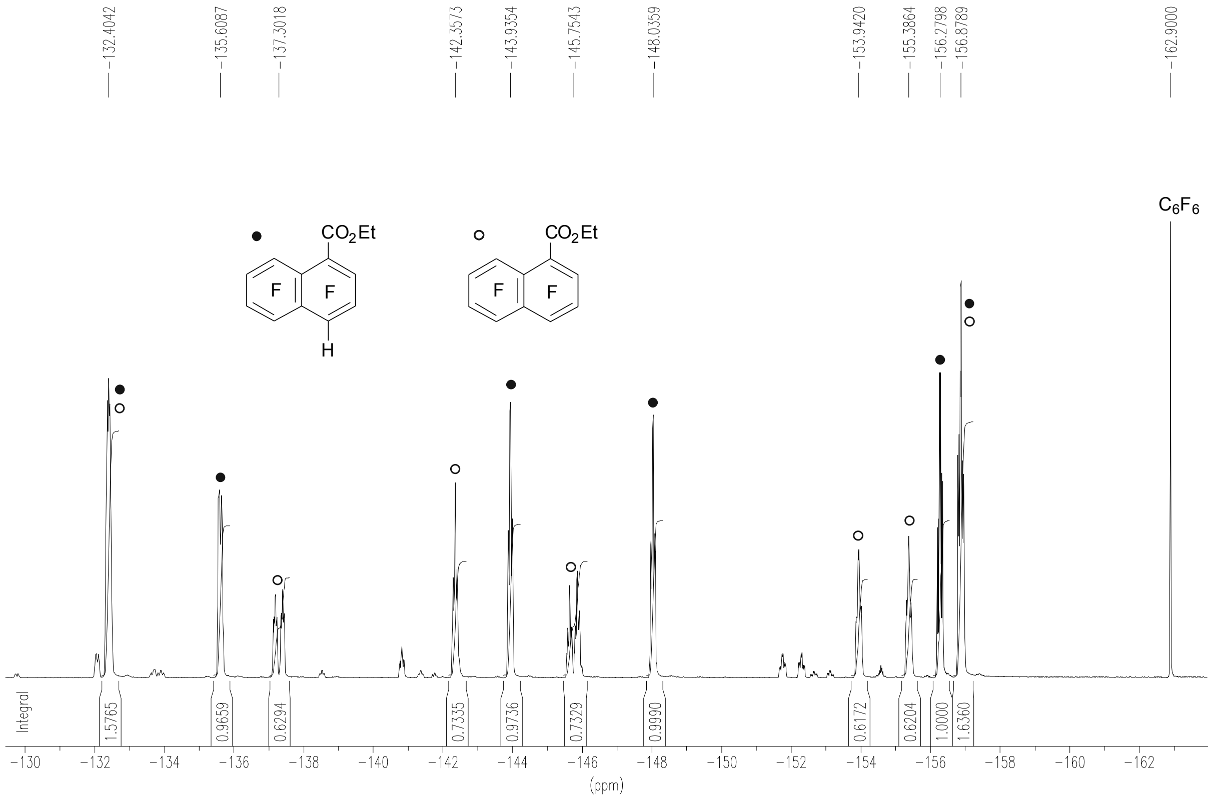

Figure S62: The ^{19}F NMR spectrum of the mixture of compounds **21Et** and **20Et** ($CDCl_3$).

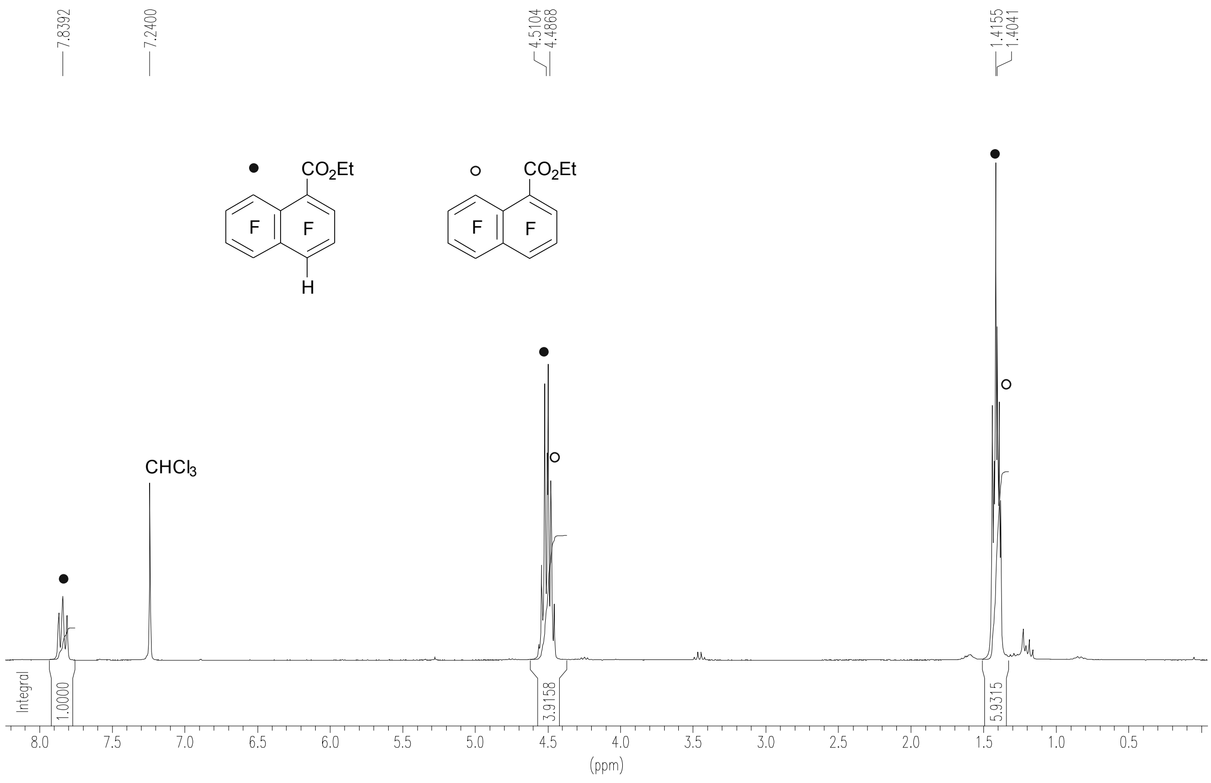

Figure S63: The ^{1}H NMR spectrum of the mixture of compounds **20Et** and **21Et** ($CDCl_3$).

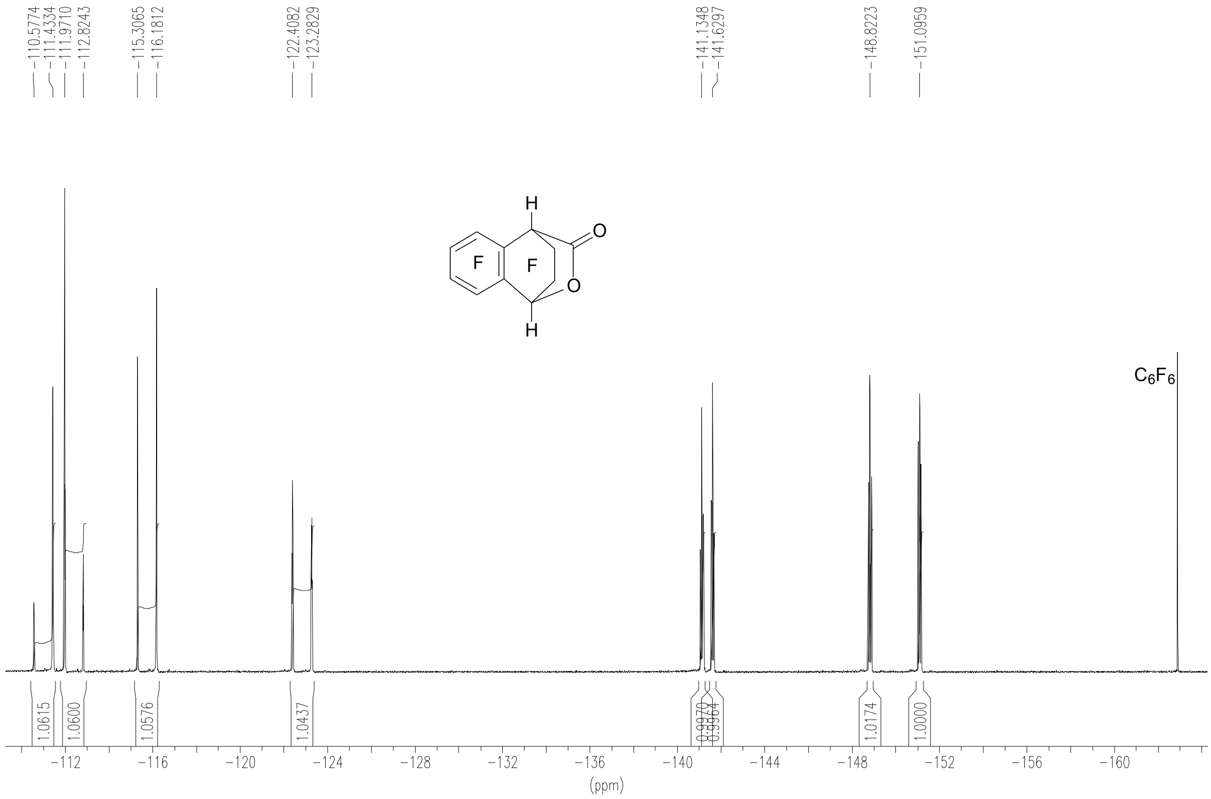

Figure S64: The ^{19}F NMR spectrum of compound **22** ($(CD_3)_2CO$).

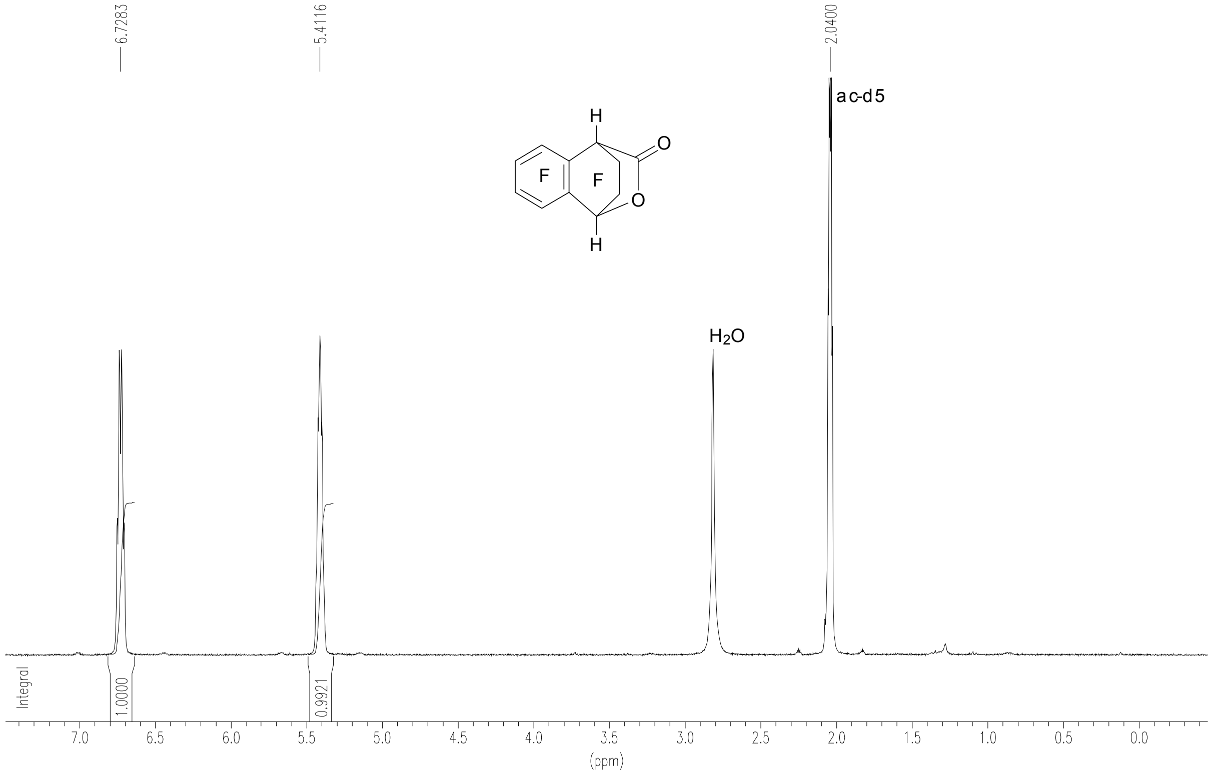

Figure S65: The ^{1}H NMR spectrum of compound **22** ($(CD_3)_2CO$).

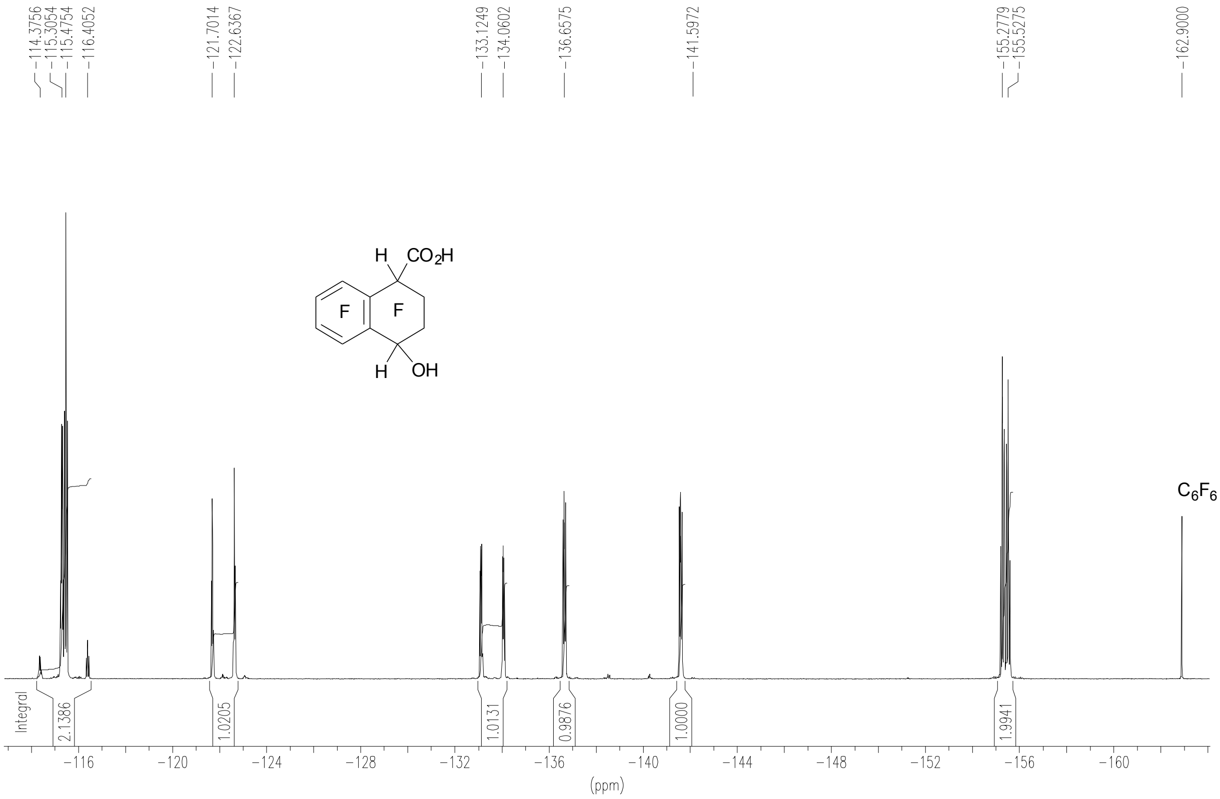

Figure S66: The ^{19}F NMR spectrum of compound **23** ($(CD_3)_2CO$).

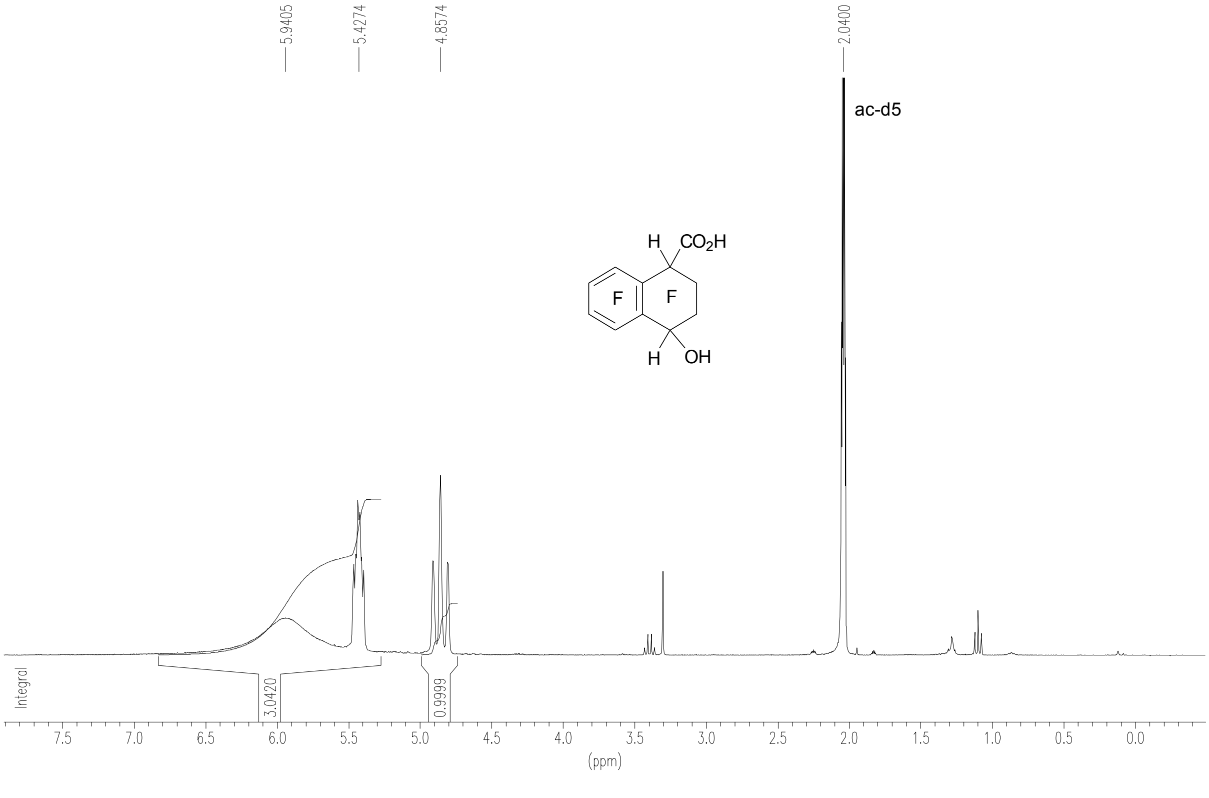

Figure S67: The ^{1}H NMR spectrum of compound **23** ($(CD_3)_2CO$).

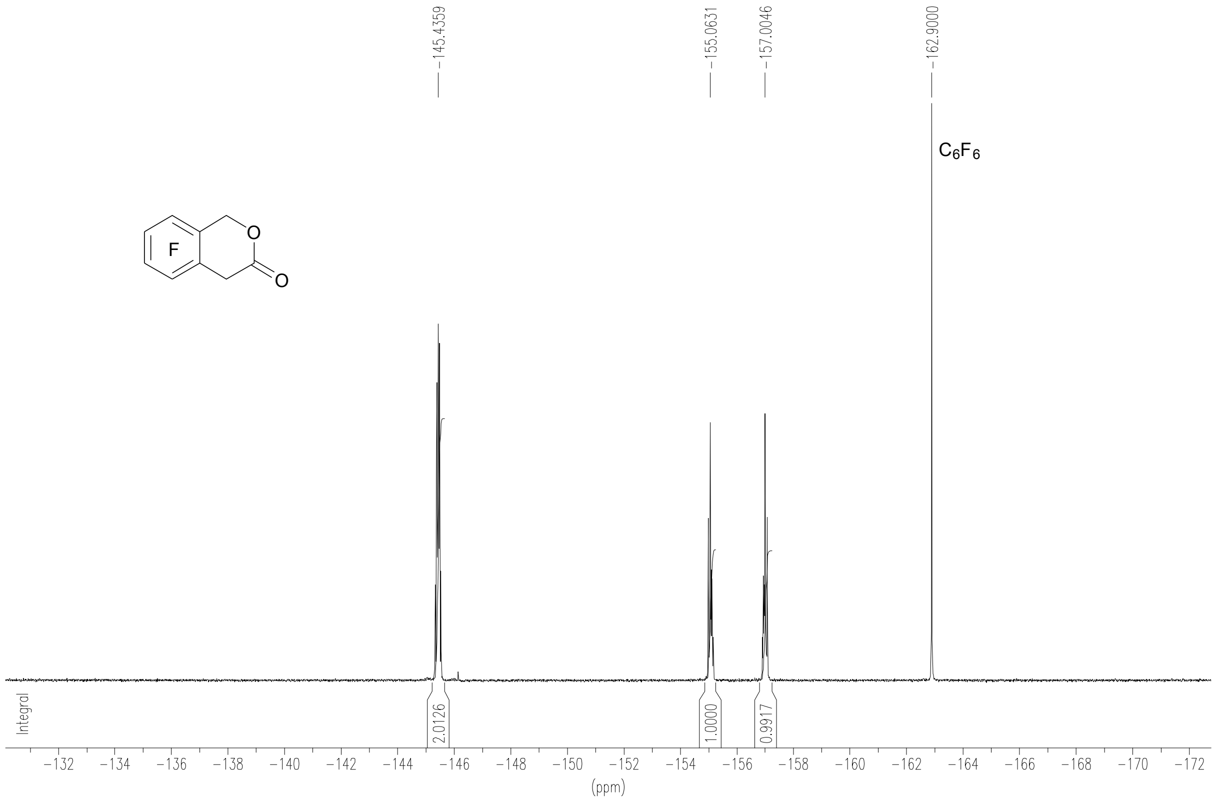

Figure S68: The ^{19}F NMR spectrum of compound **24c** ($CDCl_3$).

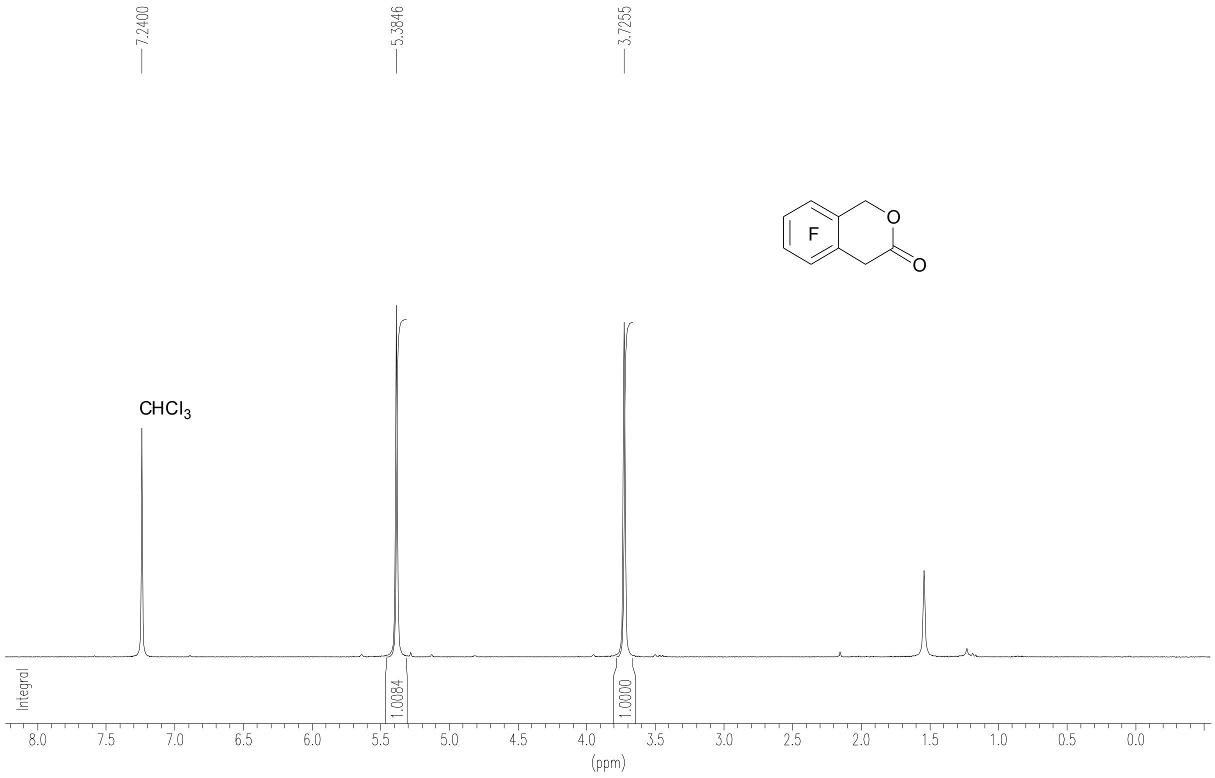

Figure S69: The ^{1}H NMR spectrum of compound **24c** ($CDCl_3$).

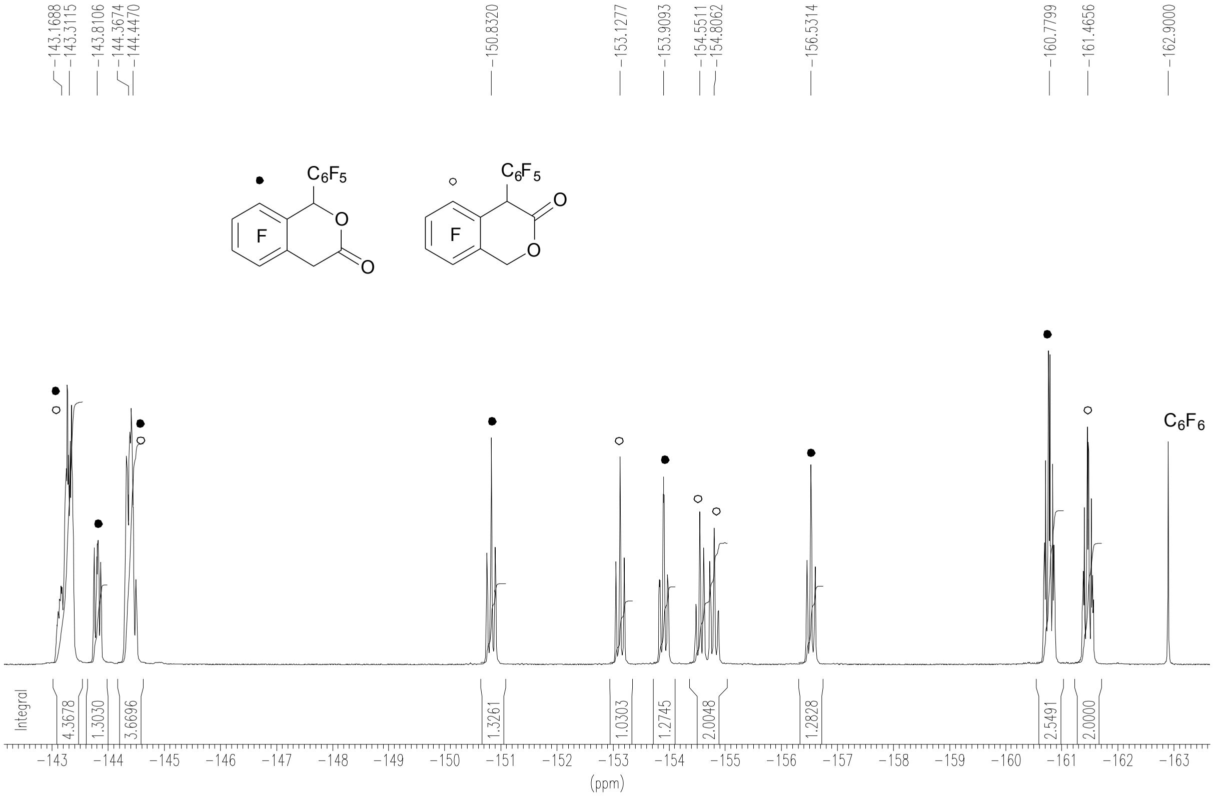

Figure S70: The ^{19}F NMR spectrum of the mixture of compounds **24d** and **25d** ($CDCl_3$).

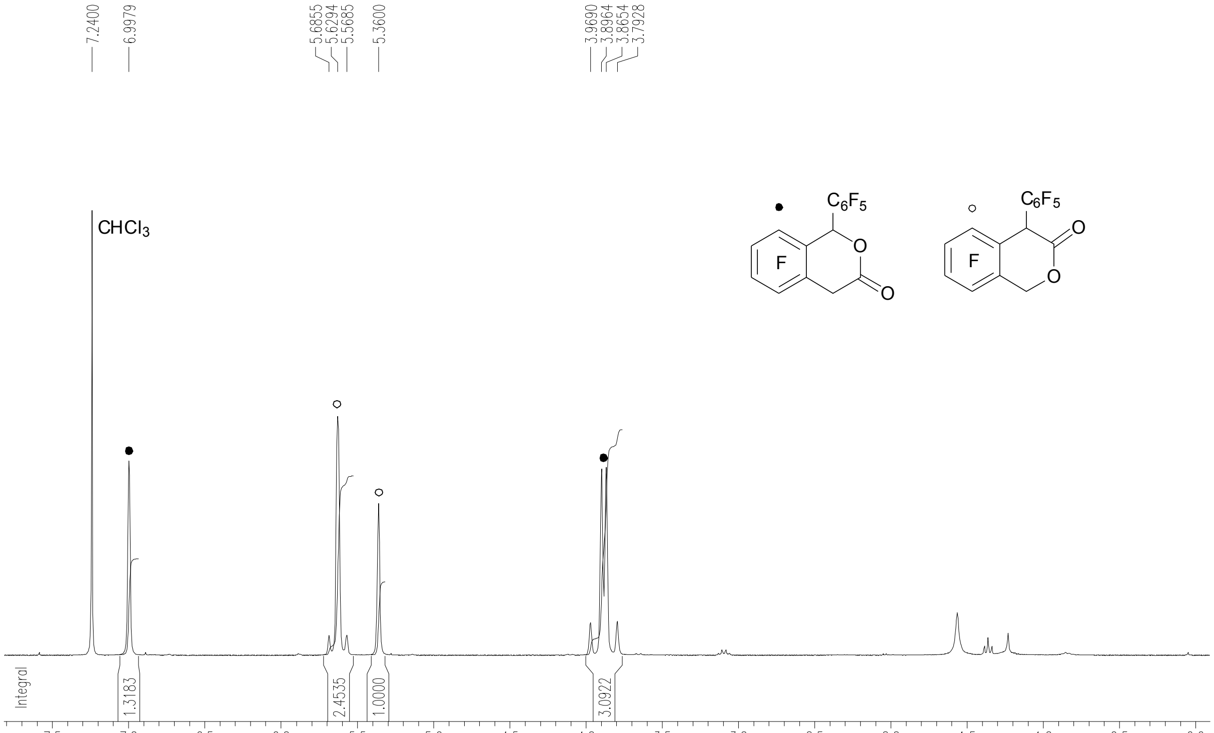

Figure S71: The ^{1}H NMR spectrum of the mixture of compounds **24d** and **25d** ($CDCl_3$).

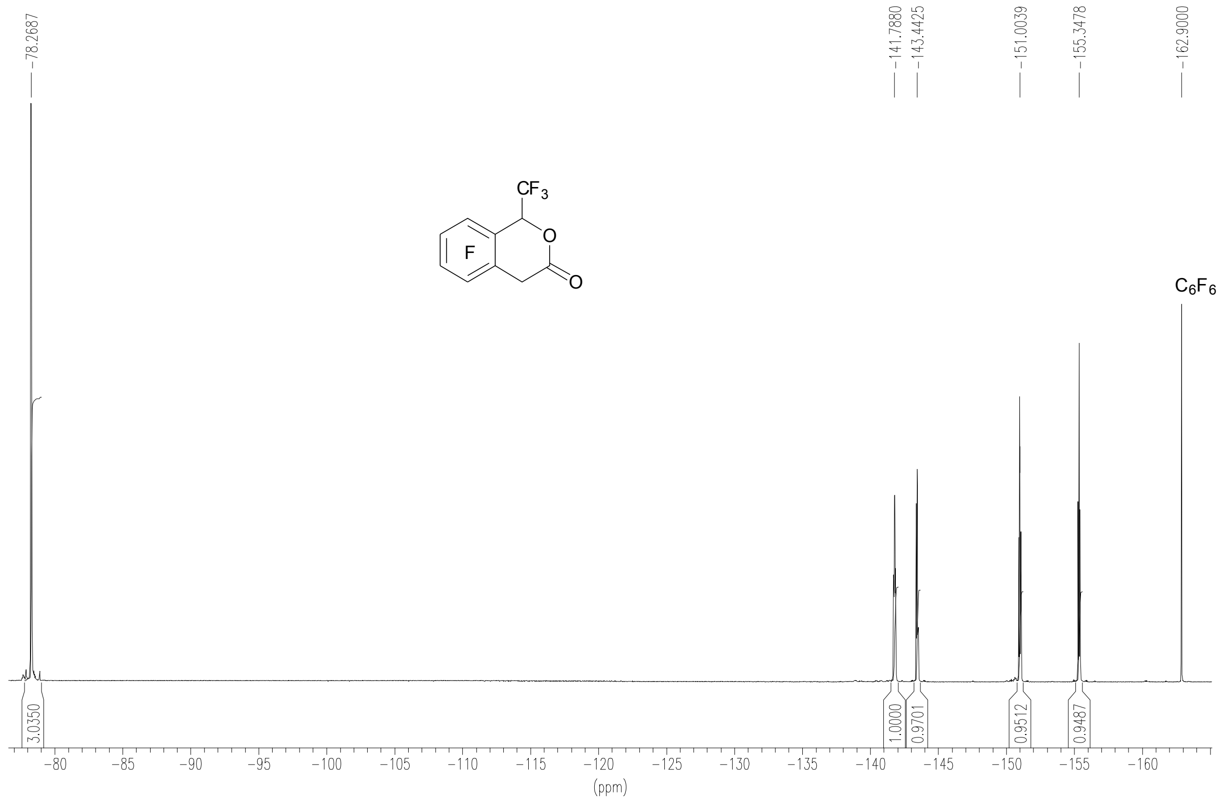

Figure S72: The ^{19}F NMR spectrum of compound **24e** ($CDCl_3$).

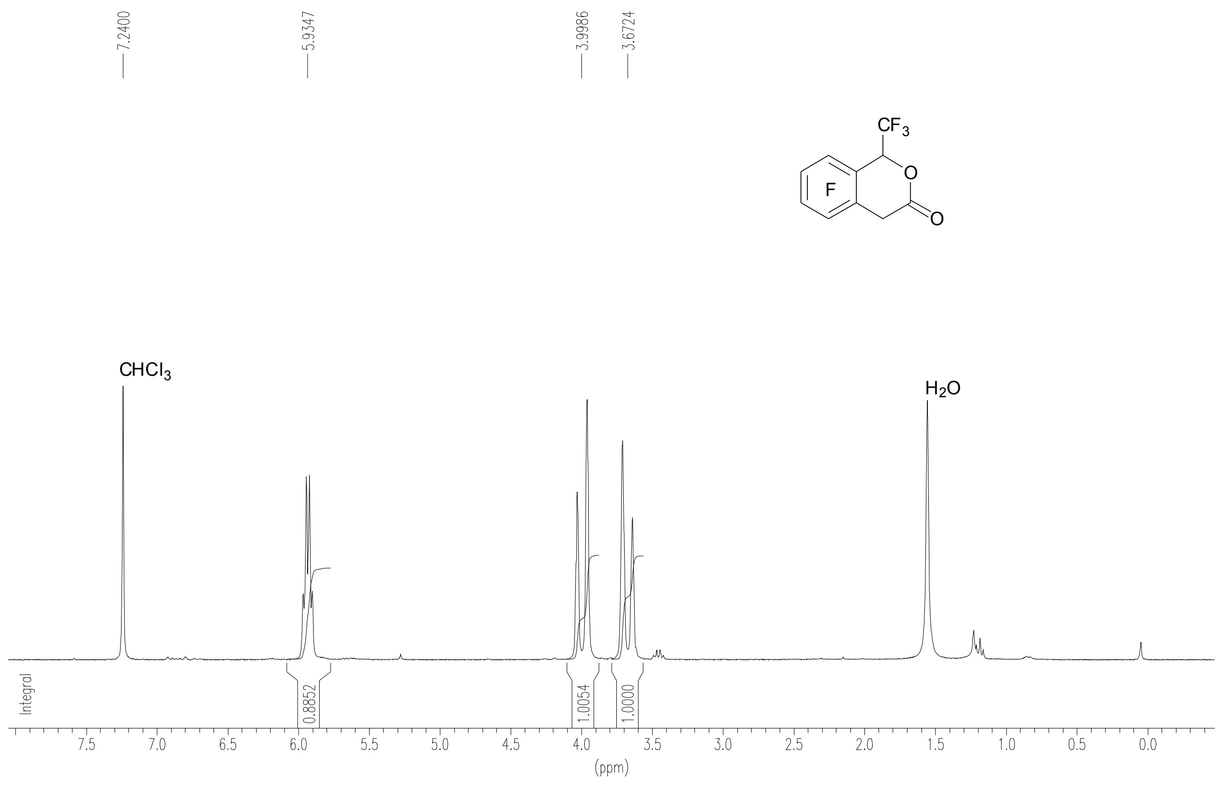

Figure S73: The ^{1}H NMR spectrum of compound **24e** ($CDCl_3$).
